# Supplementary material for: Generation of a μ-1,2-hydroperoxo FeIIIFeIII and a μ-1,2-peroxo FeIVFeIII Complex
Source: Nat Commun. 2022 Mar 16;13:1376. doi: 10.1038/s41467-022-28894-5 (PMC8927127; doi:10.1038/s41467-022-28894-5)
Supplement: Supplementary file 1 — Supplementary Information [file 41467_2022_28894_MOESM1_ESM.pdf]

Generation of a μ-1,2-Hydroperoxo $Fe^{III}Fe^{III}$ and a μ-1,2-Peroxo $Fe^{IV}Fe^{III}$ Complex

Stephan Walleck[1], Thomas Philipp Zimmermann[1], Henning Hachmeister[2], Christian Pilger[2], Thomas Huser[2], Sagie Katz[3], Peter Hildebrandt[3], Anja Stammler,[1] Hartmut Bögge[1], Eckhard Bill[4], and Thorsten Glaser[1*]

[1] Lehrstuhl für Anorganische Chemie I, Fakultät für Chemie, Universität Bielefeld, Universitätsstr. 25, D-33615 Bielefeld, Germany, [2] Biomolekulare Photonik, Fakultät für Physik, Universität Bielefeld, Universitätsstr. 25, D-33615 Bielefeld, Germany, [3] Institut für Chemie, Technische Universität Berlin, Straße des 17. Juni 135, D-10623 Berlin, Germany, [4] Max-Planck-Institut für Chemische Energiekonversion, Stiftstr. 34-36, D-45470 Mülheim an der Ruhr, Germany, * To whom correspondence should be addressed. E-mail: thorsten.glaser@uni-bielefeld.de

Supplementary Methods

Synthesis of Compounds

All syntheses were carried out under a nitrogen atmosphere using standard Schlenk techniques. Starting materials were of the highest commercially available purity and used as received. The solvents were refluxed for one day while bubbling a N_2 stream through them to remove dissolved oxygen. The ligand susan$^{6\text{-Me}}$ (= 4,7-dimethyl-1,1,10,10-tetra(6-methyl-2-pyridylmethyl)-1,4,7,10-tetraazadecane),[1] the oxidant thianthrenium perchlorate,[2] and the acid [$HPPh_3$](BF_4) [3] were synthesized according to known literature procedures, which are given below (in all cases analytical data agree with those reported in the given references). WARNING: Although we experienced no difficulties, perchlorate salts are potentially hazardous and should only be handled in small quantities and with adequate precautions. This advice has to be strictly followed in experiments with thianthrenium perchlorate which is known to cause violent explosions spontaneously.

susan$^{6\text{-Me}}$ (= 4,7-dimethyl-1,1,10,10-tetra(6-methyl-2-pyridylmethyl)-1,4,7,10-tetra-azadecane)

$NaBH(OAc)_3$ (15.1 g, 71.3 mmol) was added to a solution of 4,7-dimethyl-1,4,7,10-tetraazadecane (2.05 g, 11.8 mmol) and 6-methylpyridine-2-carboxaldehyde (6.13 g, 50.6 mmol) in 1,2-dichloroethane (70 mL). The reaction mixture was stirred for 2 hours at 60 °C and for 12 hours at room temperature. Then the reaction was quenched by addition of aqueous NaOH solution (6 M, 100 mL). The layers were separated and the aqueous phase was extracted with CH_2Cl_2 (2 × 100 mL). The combined organic fractions were washed with brine (170 mL) and dried over Na_2SO_4. Removal of solvent under reduced pressure afforded the crude product, which was purified by column chromatography (basic aluminium oxide, THF) providing the pure ligand as a yellow oil. Yield: 5.83 g (83%).

Thianthrenium perchlorate

A solution of thianthrene (0.50 g, 2 mmol) in CCl_4 (90 mL) was added to a solution of 70% perchloric acid (0.8 mL) in acetic anhydride (50 mL) and left overnight at room temperature. Dark, purple crystals deposited, that were filtered off, washed three times with CCl_4, and dried under vacuum. Yield: 0.34 g (54%).

Triphenylphosphonium tetrafluoroborate

Etheral HBF_4 (1.0 mL, 7.6 mmol) was added dropwise to a solution of PPh_3 (2.0 g, 7.6 mmol) in dry Et_2O (50 ml) resulting in precipitation of $[HPPh_3]BF_4$ as a white powder, that were filtered off, washed three times with Et_2O, and dried under vacuum. Yield: 2.4 g (90%).

Synthesis of Isotopically Labeled Complexes

The isotopic labeling experiments were carried out in a glove-box. The MeOH used for these experiment was refluxed over Mg for 18 h, distilled under a nitrogen atmosphere, and was kept over 10 Volume % of freshly activated 3 Å molecular sieve prior use.

$[(susan^{6\text{-}Me})\{Fe(\mu\text{-}^{18}OH)_2Fe\}](ClO_4)_2$. $[(susan^{6\text{-}Me})\{Fe(\mu\text{-}OH)_2Fe\}](ClO_4)_2 \cdot H_2O$ (20 mg, $2.1 \cdot 10^{-5}$ mol) was recrystallized in a 1:20 mixture of $H_2{}^{18}O$ and MeCN (2mL) by Et_2O diffusion. The yellow crystals were filtered off and washed with Et_2O. IR (KBr): $\tilde{\nu}/cm^{-1}$ = 3629 w, 2976 w, 2856 m, 1604 s, 1577 m, 1464 s, 1093 vs, 953 m, 848 m, 775 s, 623 s, 468 m. ESI-MS (+) (DCM): *m/z* = 361.2 $[(susan^{6\text{-}Me})\{Fe(\mu\text{-}O)Fe\}]^{2+}$.

$[(susan^{6\text{-}Me})\{Fe(\mu\text{-}O)(\mu\text{-}^{18}O_2)Fe\}](ClO_4)_2$. A solution of $[(susan^{6\text{-}Me})\{Fe(\mu\text{-}OH)_2Fe\}]$-$(ClO_4)_2 \cdot H_2O$ (15 mg, $1.6 \cdot 10^{-2}$mmol) in MeOH (5 mL) was transferred to a 5 mL vial, that had been cooled to -40 °C and purged with $^{18}O_2$ for 5 minutes. The resulting brown solution was kept for 2 days at -40 °C. During this time, black crystals of $[(susan^{6\text{-}Me})\{Fe(\mu\text{-}O)(\mu\text{-}^{18}O_2)Fe\}](ClO_4)_2$ deposited, that were filtered off. IR (KBr): $\tilde{\nu}/cm^{-1}$ = 2919 m, 1605s, 1573 m, 1466 s, 1454 s, 1090 vs, 1002 m, 955 m, 932 w, 843 m, 786 s, 686 m, 624 s.

ESI-MS (+) (MeCN): *m/z* = 379.2 [(susan$^{6\text{-Me}}$){Fe(μ-O)(μ-$^{18}O_2$)Fe}]$^{2+}$.

[(susan$^{6\text{-Me}}$){Fe(μ-^{18}O)(μ-$^{18}O_2$)Fe}](ClO_4)$_2$. A solution of [(susan$^{6\text{-Me}}$){Fe(μ-^{18}OH)$_2$Fe}]-(ClO_4)$_2$ (2 mg, $2\cdot10^{-6}$ mol) in MeOH (2 mL) and $H_2{}^{18}O$ ($5\cdot10^{-5}$ L) was transferred to a 1.5 mL vial , that had been cooled to -40 °C and purged with $^{18}O_2$ for 5 minutes. The resulting brown solution was kept for 2 days at -40 °C. During this time black crystals of [(susan$^{6\text{-Me}}$){Fe(μ-O)(μ-$^{18}O_2$)Fe}](ClO_4)$_2$ deposited, that were filtered off. IR (KBr): $\tilde{\nu}$/cm^{-1} = 3078 w, 2914 w, 2881 w, 2811w, 1605s, 1573 w, 1466 s, 1454 s, 1090 vs, 1002 m, 954 m, 932 w, 842 m, 785 s, 652 m, 624 s, 488 w, 475 w, 428 w. ESI-MS (+) (MeCN): *m/z* = 380.2 [(susan$^{6\text{-Me}}$){Fe(μ-^{18}O)(μ-$^{18}O_2$)Fe}]$^{2+}$.

[(susan$^{6\text{-Me}}$){Fe(μ-^{18}O)(μ-O_2)Fe}](ClO_4)$_2$. A solution of [(susan$^{6\text{-Me}}$){Fe(μ-^{18}OH)$_2$Fe}]-(ClO_4)$_2$ (5 mg, $5\cdot10^{-6}$ mol) in MeOH (2.5 mL) and $H_2{}^{18}O$ ($5\cdot10^{-5}$ L) was transferred to a vial, that had been cooled to -40 °C and purged with O_2 for 5 minutes. The resulting brown solution was kept for 6 weeks at -40 °C. During this time, black crystals of [(susan$^{6\text{-Me}}$){Fe(μ-O)(μ-$^{18}O_2$)Fe}](ClO_4)$_2$ deposited, that were filtered off. IR (KBr): $\tilde{\nu}$/cm^{-1} = 3078 w, 2918 w, 1605s, 1573 w, 1466 s, 1454 s, 1090 vs, 1002 m, 955 m, 933 w, 842 m, 832 m, 788 m, 654 m, 623 s, 491 w, 447 w. ESI-MS (+) (MeCN): *m/z* = 380.2 [(susan$^{6\text{-Me}}$){Fe(μ-^{18}O)(μ-O_2)Fe}]$^{2+}$.

Reactivity Studies

Oxidation of [(susan$^{6\text{-Me}}$){Fe^{III}(μ-O)(μ-1,2-O_2)Fe^{III}}]$^{2+}$

In a UV-Vis cuvette under N_2 protecting atmosphere, a solution of 0.99 equivalents thianthrenium perchlorate in CH_3CN (18 mM) was added to a dark purple solution of [(susan$^{6\text{-Me}}$){Fe(μ-O)(μ-O_2)Fe}](ClO_4)$_2$ (0.65 mM) at -60 °C in CH_3CN:CH_2Cl_2 (1:1). For tests of stability a similar procedure was performed at -40 °C using pure CH_3CN as solvent.

The preparation of a Mössbauer sample was performed in a Mössbauer sample holder

for liquids. A pre-cooled solution of thianthrenium perchlorate in CH_3CN (0.25 mL of a $2.0\cdot10^{-2}$ M solution, 1.7 eq.) was added dropwise at -45 °C to a dark purple solution of ~25% ^{57}Fe-enriched [(susan$^{6\text{-}Me}$){Fe(μ-O)(μ-O_2)Fe}](ClO_4)$_2$ (3.0 mg, $3.1\cdot10^{-6}$ mol) in CH_3CN (0.5 mL). The resulting dark green solution was stirred at -45 °C for 30 s before freezing in liquid nitrogen. After measuring the Mössbauer spectrum at 80 K, the reductant NEt_3 (10 μL, $7\cdot10^{-5}$ mol) was added to the dark green solution at -45 °C resulting in an immediate color change back to dark purple. The Mössbauer spectrum of this solution at 80 K shows the known quadrupole doublet of [(susan$^{6\text{-}Me}$){Fe(μ-O)(μ-O_2)Fe}](ClO_4)$_2$ in frozen CH_3CN solution.

Protonation of [(susan$^{6\text{-}Me}$){Fe^{III}(μ-O)(μ-1,2-O_2)Fe^{III}}]$^{2+}$

In a UV-Vis cuvette under N_2 protecting atmosphere, 1.0 equivalents of a solution of perchloric acid in CH_3CN:CH_2Cl_2 (typically ~10 mM) was added to a dark purple black solution of [(susan$^{6\text{-}Me}$){Fe(μ-O)(μ-O_2)Fe}](ClO_4)$_2$ (of typically ~0.8 mM) at -60 °C in CH_3CN:CH_2Cl_2 (1:1). The job plot analysis was performed under similar conditions adding portions of 0.5 equivalents of perchloric acid. For tests of stability in CH_3CN, 1.0 equivalent of a solution of perchloric acid in CH_3CN was added at -40 °C.

The preparation of a Mössbauer sample was performed in a Mössbauer sample for liquids. A pre-cooled solution of perchloric acid in CH_3CN (50 μL of a $7.4\cdot10^{-4}$ M solution, 1.5 eq.) was added to a dark purple solution of ~25% ^{57}Fe enriched [(susan$^{6\text{-}Me}$){Fe(μ-O)(μ-O_2)Fe}](ClO_4)$_2$ (2.4 mg, $2.6\cdot10^{-6}$ mol) in CH_3CN (0.7 mL) at -45 °C. The resulting deep red solution was stirred at -45 °C for 30 s before freezing in liquid nitrogen.

Nucleophilic Reactivity of [(susan$^{6\text{-}Me}$){Fe^{III}(μ-O)(μ-1,2-O_2)Fe^{III}}]$^{2+}$

DL-2-Phenylpropionaldehyde (20 μL, 9.5 eq.) was added to a solution of [(susan$^{6\text{-}Me}$){Fe(μ-O)(μ-O_2)Fe}](ClO_4)$_2$ (15.7 mM) in MeCN at -5 °C. The solution was stirred at -5

°C for 5 days resulting in brownish suspension, which was diluted with the fourfold volume of CH_2Cl_2. This suspension was filtered over a short silica column with CH_2Cl_2 as eluent. Volatiles were removed at reduced pressure resulting in a colorless liquid (18 mg). ^{1}H NMR spectroscopy revealed acetaldehyde and DL-2-phenylpropionaldehyde as the major ingredients with a ratio of 1 : 8±2.

Electrophilic Reactivity of [(susan$^{6\text{-Me}}$){Fe^{III}(μ-O)(μ-1,2-O$_2$)Fe^{III}}]$^{2+}$

A solution of the substrate in CH_3CN was added to a solution of [(susan$^{6\text{-Me}}$){Fe(μ-O)(μ-O$_2$)Fe}](ClO_4)$_2$ (typical concentration of 0.60 – 0.95 mM) in CH_3CN at -40 °C and the reactions were followed by UV-Vis-NIR spectroscopy. Substrates used were PPh_3 (7 equivalents), 9,10-dihydroanthracene (DHA, 100 equivalents), and 1-hydroxy-2,2,6,6-tetramethylpiperidine (TEMPOH, 100 equivalents).

Electrophilic Reactivity of [(susan$^{6\text{-Me}}$){Fe^{IV}(μ-O)(μ-1,2-O$_2$)Fe^{III}}]$^{3+}$

A solution of thianthrenium perchlorate (1.0 eq.) in CH_3CN/CH_2Cl_2 (1:1) was added to a solution of [(susan$^{6\text{-Me}}$){Fe^{III}(μ-O)(μ-O$_2$)Fe^{III}}](ClO_4)$_2$ (typical concentration of 0.50 – 0.95 mM) in CH_3CN/CH_2Cl_2 (1:1) at -60 °C. After the formation of [(susan$^{6\text{-Me}}$){Fe^{IV}(μ-O)(μ-O$_2$)Fe^{III}}]$^{3+}$ was complete, a solution of the substrate in CH_3CN/CH_2Cl_2 (1:1) was added at -60 °C. The reactions were followed by UV-Vis-NIR spectroscopy. Substrates used were PPh_3 (20 equivalents) and 1-hydroxy-2,2,6,6-tetramethylpiperidine (TEMPOH, 100 equivalents).

Due to the relatively low solubility of DHA in CH_3CN/CH_2Cl_2 (1:1) at -60 °C and the restricted stability of [(susan$^{6\text{-Me}}$){Fe^{IV}(μ-O)(μ-O$_2$)Fe^{III}}]$^{3+}$, the experiments using 9,10-dihydroanthracene (DHA) were modified: a solution of thianthrenium perchlorate (1.0 eq.) in CH_3CN/CH_2Cl_2 (1:1) was added to a solution of [(susan$^{6\text{-Me}}$){Fe^{III}(μ-O)(μ-O$_2$)Fe^{III}}](ClO_4)$_2$ (0.5 mM) and DHA (100 eq.) in CH_3CN/CH_2Cl_2 (1:1) at -60 °C. In separate experiments, thianthrenium perchlorate showed no reactivity towards DHA.

Electrophilic Reactivity of [(susan^{6-Me}){Fe^{III}(μ-O)(μ-1,2-OOH)Fe^{III}}]$^{3+}$

A solution of $HClO_4$ (1.0 eq.) in CH_3CN/CH_2Cl_2 (1:1) was added to a solution of [(susan^{6-Me}){Fe^{III}(μ-O)(μ-O_2)Fe^{III}}](ClO_4)$_2$ (typical concentration of 0.50 – 0.95 mM) in CH_3CN/CH_2Cl_2 (1:2) at -60 °C. After the formation of [(susan^{6-Me}){Fe^{III}(μ-O)(μ-OOH)Fe^{III}}]$^{3+}$ was complete, a solution of the substrate in CH_3CN/CH_2Cl_2 (1:2) was added at -60 °C. The reactions were followed by UV-Vis-NIR spectroscopy. Substrates used were PPh_3 (20 equivalents) and 1-hydroxy-2,2,6,6-tetramethylpiperidine (TEMPOH, 100 equivalents).

Due to the relatively low solubility of DHA in CH_3CN/CH_2Cl_2 (1:2) at -60 °C and the restricted stability of [(susan^{6-Me}){Fe^{III}(μ-O)(μ-OOH)Fe^{III}}]$^{3+}$, the experiments using 9,10-dihydroanthracene (DHA) were modified: a solution of $HClO_4$ (1.0 eq.) in CH_3CN/CH_2Cl_2 (1:1) was added to a solution of [(susan^{6-Me}){Fe^{III}(μ-O)(μ-O_2)Fe^{III}}](ClO_4)$_2$ (0.5 mM) and DHA (100 eq.) in CH_3CN/CH_2Cl_2 (1:2) at -60 °C. In separate experiments, $HClO_4$ showed no reactivity towards DHA.

Determination of pK_a [4] of [(susan^{6-Me}){Fe^{III}(μ-O)(μ-1,2-OOH)Fe^{III}}]$^{3+}$

One equivalent of a solution of a given acid in MeCN (30 – 60 mM) was added to a solution of [(susan^{6-Me}){Fe^{III}(μ-O)(μ-1,2-O_2)Fe^{III}}](ClO_4)$_2$ in MeCN (typical concentration of 0.5 – 0.9 mM) at – 40 °C. The change of the UV/vis spectrum within the first 20 seconds was measured. Plotting the change of the normalized absorbance at 15400 cm^{-1} *vs* the pK_a of the acid added[5–8] results in a S-shaped curve, which has been fitted with a sigmoidal Boltzmann function with a Levenberg Marquardt iteration algorithm in Origin 2016.

Crystal Structure Determination

Single crystals of [(susan$^{6\text{-Me}}$){Fe^{II}(μ-OH)$_2$$Fe^{II}$}]($ClO_4$)$_2$•$CH_3OH$, [(susan$^{6\text{-Me}}$){$Fe^{III}$($\mu$-O)($\mu$-1,2-$O_2$)$Fe^{III}$}]($ClO_4$)$_2$•0.85$CH_3CN$•0.7$H_2O$, and [(susan$^{6\text{-Me,ox}}$){$Fe^{III}(OH)_{0.2}$($\mu$-O)$Fe^{III}$-(OH)$_{0.65}$}($ClO_4$)$_2$ were removed from the mother liquor, coated with oil, and measured at 100(2) K on a Bruker KAPPA APEX II (four circle diffractometer with 4K CCD detector, CuKα radiation, Quazar™ Montel multilayer optics). Empirical absorption corrections using equivalent reflections were performed with the program SADABS-2016/2.[9] The structures were solved and refined with the programs SHELXT/L[10–12] using OLEX2.[13] Crystal data and details concerning data collections and structure refinements are given in Table S1.

In [(susan$^{6\text{-Me}}$){Fe^{II}(μ-OH)$_2$$Fe^{II}$}]($ClO_4$)$_2$•$CH_3OH$ the hydrogen atoms on the coordinated hydroxides were found and refined. Three oxygen atoms of one perchlorate counter ion show disorder. In [(susan$^{6\text{-Me,ox}}$){Fe^{III}(OH)$_{0.2}$(μ-O)Fe^{III}(OH)$_{0.65}$}](ClO_4)$_2$ a disorder of a CH_3CN solvent molecule and one perchlorate counter ion with four water molecules is observed. The hydrogen atoms on these disordered water molecules were not found and not generated. Crystals of [(susan$^{6\text{-Me,ox}}$){Fe^{III}(OH)$_{0.2}$(μ-O)Fe^{III}(OH)$_{0.65}$}](ClO_4)$_2$ show a disorder in the ligand framework. Separated electron density maxima were observed for some carbon atoms of the pyridine ring coordinated by N44 to Fe2 and next to the methyl group C76. We interpreted these positions as resulting from a partly oxidation of the methyl group to an alkoxide, which partially replaces the coordinated hydroxide O54. Best results of the refinement were obtained with 35 % occupation for the positions of the oxidized species and 65 % for the original configuration. Anisotropic displacement parameters of the disordered positions were refined with ISOR constraints. Another disorder was resolved for the methyl group C36 bound to the pyridine ring containing N4 coordinated to Fe1. Here we interpreted the residual electron density around C36 as resulting from an oxidation of the methyl group to a carboxylate, which partly replaces the

coordinated hydroxide O53. Best results of the refinement were obtained with 80 % occupation for the positions of the oxidized species and 20 % for the original configuration. For this disorder only the anisotropic displacement of the minority species (C36 and O53) were refined with ISOR constraints. Due to the disorder, hydrogen atoms of partially occupied coordinated hydroxides were not found and not generated.

Supplementary Table 1. Crystal data and refinement parameters.

	$[(susan^{6\text{-}Me})\{Fe^{II}(\mu\text{-}OH)_2Fe^{II}\}](ClO_4)_2{\bullet}CH_3OH$	$[(susan^{6\text{-}Me})\{Fe^{III}(\mu\text{-}O)(\mu\text{-}1,2\text{-}O_2)Fe^{III}\}](ClO_4)_2{\bullet}0.85CH_3CN{\bullet}0.7H_2O$	$[(susan^{6\text{-}Me,ox})\text{-}\{Fe^{III}(OH)_{0.2}(\mu\text{-}O)Fe^{III}\text{-}(OH)_{0.65}\}](ClO_4)_2$
Empirical formula	$C_{37}H_{56}Cl_2Fe_2N_8O_{11}$	$C_{37.70}H_{53.95}Cl_2Fe_2N_{8.85}O_{11.70}$	$C_{36}H_{48.10}Cl_2Fe_2N_8O_{11.80}$
Formula weight	971.49	1000.94	964.32
Temperature /K	100(2)	100(2)	100(2)
Crystal system	monoclinic	monoclinic	monoclinic
Space group	$P2_1/c$	$P2_1/c$	*Cc*
a / Å	10.0588(4)	11.9892(3)	19.425(2)
b / Å	32.3908(14)	16.5184(4)	14.3243(16)
c / Å	13.8402(6)	21.5713(6)	14.7450(14)
α / °	90	90	90
β / °	110.875(2)	92.5190(10)	98.579(4)
γ /°	90	90	90
V / Å^3	4213.3(3)	4267.90(19)	4056.9(8)
Z	4	4	4
ρ / g cm^{-3}	1.532	1.558	1.579
μ / mm^{-1}	7.269	7.216	7.564
F(000)	2032.0	2087.0	2002.0
Crystal size / mm^3	0.33 × 0.24 × 0.16	0.19 × 0.11 × 0.04	0.20 × 0.07 × 0.03
Radiation	CuKα	CuKα	CuKα
2Θ range / °	5.46 to 136.66	6.74 to 136.86	7.70 to 136.47
hkl ranges	$-12 \leq h \leq 12$<br>$-39 \leq k \leq 36$<br>$-16 \leq l \leq 16$	$-14 \leq h \leq 14$<br>$-19 \leq k \leq 19$<br>$-25 \leq l \leq 25$	$-23 \leq h \leq 23$<br>$-17 \leq k \leq 17$<br>$-16 \leq l \leq 17$
Collected refl.	79133	65620	21431
Unique refl., R_{int}	7712, 0.0315	7834, 0.0379	6337, 0.0335
Observed refl. ($I > 2\sigma(I)$)	7615	7515	5988
Completeness	0.998	0.998	0.997
Absorption correction	multi-scan	multi-scan	multi-scan
Data/restraints/parameters	7712/39/585	7834/33/630	6337/58/637
Goodness-of-fit on F^2	1.052	1.050	1.035
R_1, wR_2 ($I > 2\sigma(I)$)	0.0253, 0.0655	0.0271, 0.0715	0.0431, 0.1149
R_1, wR_2 (all data)	0.0257, 0.0657	0.0283, 0.0723	0.0459, 0.1176
Largest peak/hole / e Å^{-3}	0.40/-0.53	0.41/-0.45	0.61/-0.45
CCDC numbers	2072804	2072806	2072805

Supplementary Fig. 1. Thermal ellipsoid plots of a) $[(\mathrm{susan}^{6\text{-}Me})\{Fe^{II}(\mu\text{-}OH)_2Fe^{II}\}]^{2+}$ in single-crystals of $[(\mathrm{susan}^{6\text{-}Me})\{Fe^{II}(\mu\text{-}OH)_2Fe^{II}\}](ClO_4)_2{\bullet}CH_3OH$ and b) $[(\mathrm{susan}^{6\text{-}Me})\{Fe^{III}(\mu\text{-}O)(\mu\text{-}O_2)Fe^{III}\}]^{2+}$ in single-crystals of $[(\mathrm{susan}^{6\text{-}Me})\{Fe^{III}(\mu\text{-}O)(\mu\text{-}O_2)Fe^{III}\}](ClO_4)_2$-$\bullet 0.85CH_3CN{\bullet}0.7H_2O$. c) Two different thermal ellipsoid plots are provided for the decay product $[(\mathrm{susan}^{6\text{-}Me,ox})\{Fe^{III}(OH)_{0.2}(\mu\text{-}O)Fe^{III}(OH)_{0.65}\}](ClO_4)_2$ to illustrate the disorder in the ligand framework. Top: majority species of 80% carboxylate at Fe1 and 65% hydroxide at Fe2. Bottom: minority species of 20% hydroxide at Fe1 and 35% benzylalcoholato at Fe2. Thermal ellipsoids are drawn at the 50% probability level.

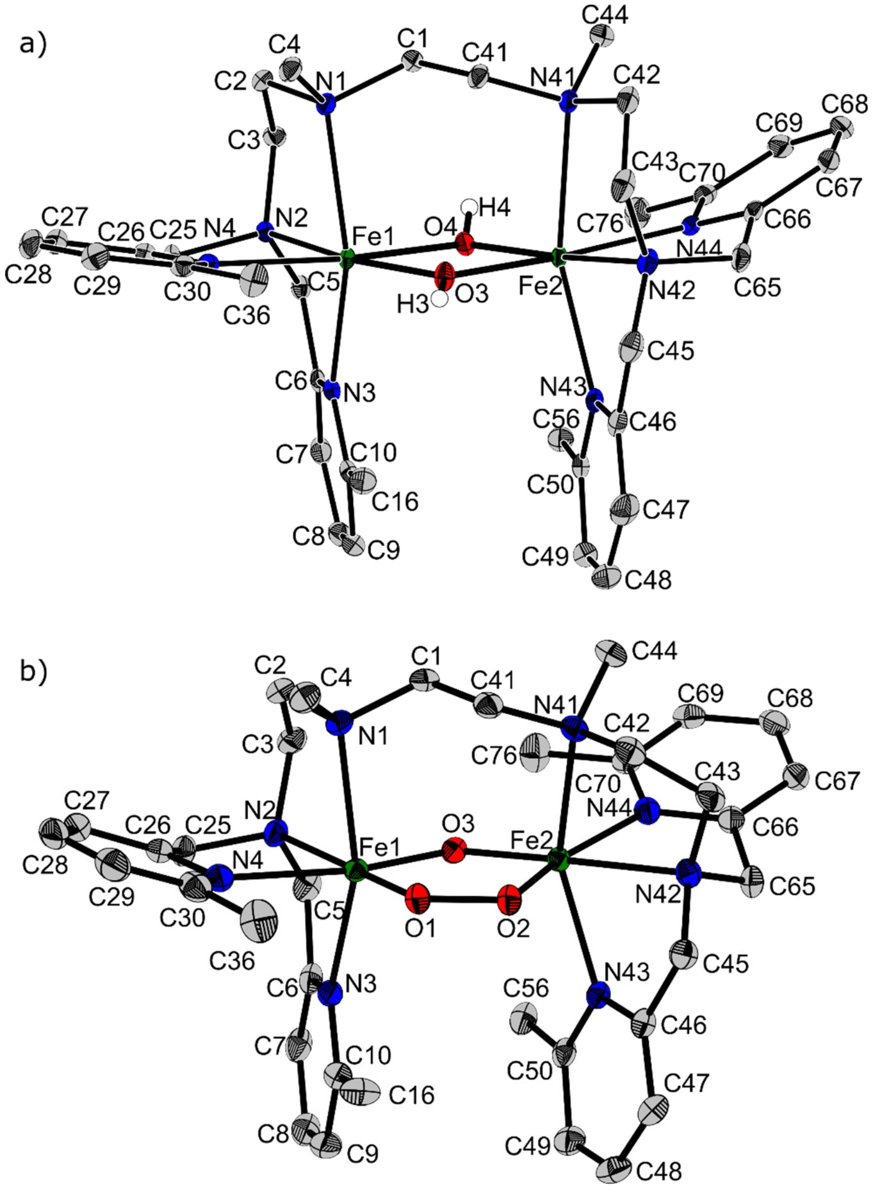

c)

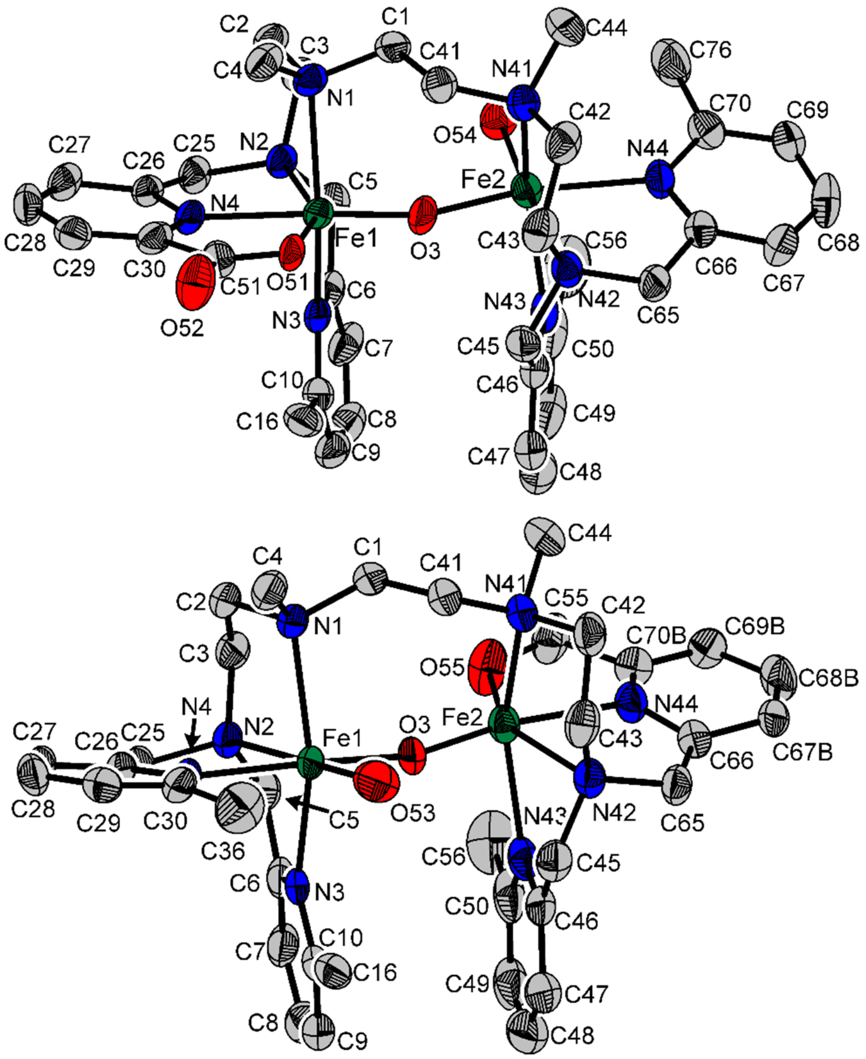
C2
C1
C44
C76
C3
C41
C4
N41
N1
C70
C69
C42
C27
C25
O54
N2
N44
C26
C5
Fe2
N4
Fe1
O3
C43
C56
C68
C28
C29
C30
C66
C51
O51
C6
N43
N42
C67
C65
O52
N3
C7
C45
C50
C10
C46
C16
C8
C49
C9
C47
C48
C44
C4
C1
C41
N41
C55
C42
C2
N1
C69B
C70B
C3
O55
C68B
N4
N44
C27
C26
C25
N2
Fe1
O3
Fe2
C43
C67B
C66
C28
C29
C30
O53
N42
C65
C5
N43
C36
C56
C45
C6
N3
C50
C46
C10
C7
C16
C49
C47
C8
C9
C48

Other Physical Measurements

Infrared spectra (400-4000 cm^{-1}) of solid samples were recorded on a Bruker Vertex 70 as KBr disks. ESI mass spectra were recorded on a Bruker Esquire 3000 ion trap mass spectrometer equipped with a standard ESI source. UV-Vis-NIR absorption spectra were measured on a JASCO V770 spectrophotometer at -10 °C or on a Agilent 8453 diode array spectrometer coupled to an Unisoko USP-203-A cryostat for temperature control at the temperatures provided.

Cyclic and square-wave voltammograms (CVs and SWs) were measured by use of an EG&G potentiostat/galvanostat 273A on Ar-flushed solutions containing 0.2 M $TBAPF_6$ as supporting electrolyte in a conventional electrochemical cell. The working electrode was a GC electrode, the counter electrode was a platinum wire, and the reference electrode was Ag/0.01 M $AgNO_3/CH_3CN$. Controlled potential coulometric measurements were performed on $2.5 \cdot 10^{-4}$ M solutions in CH_3CN using temperature-controlled 50 mL Schlenk flasks under a nitrogen atmosphere, which were cooled to -40 °C by an in-line circulation cryostat (Huber unistat 830). The working electrode was a platinum net electrode, the counter electrode was a coiled platinum wire, and has been separated from the rest of the solution by a glass frit, and the reference electrode was Ag/0.01 M $AgNO_3/CH_3CN$. The coulometric redox processes were followed by UV-vis-NIR absorption spectoscopy on a J&M TIDAS II spectrometer equipped via fibre optics with a dip probe (Hellma 661.087-UVS with a slit width of 1 cm). The potentials were referenced versus the ferrocenium/ferrocene (Fc^+/Fc) couple used as an internal standard.

Magnetic susceptibility data were measured on powdered samples in the temperature range 2-300 K by using a SQUID magnetometer (Quantum Design MPMS XL-7 EC) with a field of 1.0 T. For calculations of the molar magnetic susceptibilities, χ_m, the measured susceptibilities were corrected for the underlying diamagnetism of the sample holder and the sample by using tabulated Pascal's constants. The susceptibility data were analyzed on the basis of the usual spin-Hamiltonian description for the electronic ground state of

exchange coupled systems. The program package JulX was used for spin-Hamiltonian simulations and fittings of the data by a full-matrix diagonalization approach (E. Bill, in-house program). The spin-Hamiltonian employed was:

$$\hat{H} = -2J\hat{\vec{\mathbf{S}}}_1\hat{\vec{\mathbf{S}}}_2 + \sum_{i=1,2} g\mu_B\hat{\vec{\mathbf{S}}}_i\vec{\mathbf{B}} \quad (1)$$

where J is the exchange coupling constant and g is the average electronic g value. Magnetic moments were obtained from numerically generated derivatives of the eigenvalues of eq. 1, and summed up over 16 field orientations along a 16-point Lebedev grid to account for the powder distribution of the sample.

^{57}Fe Mössbauer spectra were recorded on an alternating constant-acceleration spectrometer. The minimal line-width was 0.24 mm s^{-1} full-width at half-height. The sample temperature was maintained constant in a bath cryostat (Wissel MBBC-HE0106). ^{57}Co/Rh was used as the radiation source. Isomer shifts were determined relative to α-iron at room temperature.

Resonance Raman spectroscopy was performed with a confocal Raman microscope (Horiba, LabRAM Aramis) utilizing the Helium-Neon-laser light source at 633 nm with a focal intensity of 10 mW. The light was focused onto the solid samples by a 100× objective lens (NA = 0.9) and then separated and filtered by an optical long pass filter at 633 nm. The filtered Raman signals are focused through a slit (115 μm clearance) for enhancing the spectral resolution and are then coupled into a Czerny-Turner spectrograph for wavelengths separation by an optical reflection grating (1800 groves mm^{-1}). The final detection of the Raman spectrum is performed by a CCD-based line scan camera (Horiba). The acquisition time was set to 30 s per spectrum and nine single spectra were averaged for further signal-to-noise improvement. All spectra were scaled using the ν(O-O) band. Calibration was performed on a piece of silicon wafer. Prior to Raman spectroscopy bright-field imaging was performed to identify a flat area of the crystal surface, where the lateral focal position of the Raman laser was set. Following the axial

focal position was optimized by observing the back reflex of the fundamental He-Ne-laser light.

Resonance Raman spectra in solution were acquired by a custom-built confocal Raman-microscopy setup utilizing the 647 nm laser line of an Ar-Kr gas laser source (Coherent, Innova 70C Spectrum), which was selected by an acousto-optic tunable filter (AA Optoelectronics, AOTFnc-VIS) and then further filtered by a laser cleanup filter (Semrock, HC Laser Clean-up Max Line 647.1/2.5). The beam size is magnified by a 2,5x telescope to fill the back focal plane of a 10x air objective lens (Olympus,UPlanFl, NA = 0.3), which is focusing the light into the sample. The focal laser intensity was set to 15 mW. The backscattered Raman signals are separated from the excitation laser path by a long pass beam splitter (Semrock, HC BS 649), which reflects the 647 nm laser light and transmits the Raman signals in epi-direction. The Raman scattered light is further filtered (Semrock, LP 647 RU Razor Edge) and coupled into a multimode optical fiber (Thorlabs, M96L02, 105µm core size) using a lens with 100 mm focal length (Thorlabs, AC254-100-B-ML). The fiber transfers the signals into a spectrometer (Princeton Instruments, Acton 2300i) acting also as a pinhole to gain better focal sectioning in the sample. The wavelengths separation is performed in the spectrometer by selecting a 600 groves /mm reflecting grating (blaze wavelength 500 nm). A spectroscopy CCD-camera (Andor, DU401-BR-DD) is detecting the final spectrum. The initial calibration was acquired using a toluene sample at room temperature by selecting four prominent Raman peaks in the range of 520 – 1003 cm^{-1}. Samples were prepared with a typical concentration of 20mM in MeCN and directly frozen onto an aluminum cold finger in thermal contact to liquid nitrogen reservoir. In order to avoid ice accretion dry nitrogen was flowed over the sample. All spectra were acquired over 10 seconds and 10 single spectra are averaged for noise reduction using the cosmic ray removal function of the camera acquisition software.

Further measurements were performed to obtain resonance Raman spectra of the oxidized complex $[(susan^{6\text{-}Me})\{Fe^{IV}(\mu\text{-}O)(\mu\text{-}1,2\text{-}O_2)Fe^{III}\}]^{3+}$ and the protonated complex

$[(susan^{6\text{-}Me})\{Fe^{III}(\mu\text{-}O)(\mu\text{-}1,2\text{-}OOH)Fe^{III}\}]^{3+}$ using a different setup. For these experiments 3-5 μl of a precooled sample (-60 °C, ~20 mM) were pipetted onto a quartz plate cooled with liquid N_2 under a dry N_2 atmosphere and were inserted into a pre-cooled THMS600 Linkam cryostat (80K). Measurements were conducted using a LabRam HR-800 (Jobin Yvon) confocal Raman spectrometer equipped with a Symphony II CCD camera (Horiba) cooled with liquid N_2. Kr laser (tuned to either 568 nm or 647 nm) or Ar laser (tuned to 514 nm) were used as excitation sources. The Raman shift was subsequently calibrated using an external standard (toluene) recorded before and after the measurement of the samples.

Computational Details

All calculations were performed using ORCA 4.2[14–16] with the ZORA scalar relativistic method.[17] Relativistically recontracted versions of the Karlsruhe def2-TZVP basis sets for iron and the coordinating atoms were used together with the auxiliary basis set def2/J.[18] For the C and H atoms the according SVP basis set[19,20] together with the auxiliary basis set def2/J was used.[18] The solvation model CPCM with a dielectric constant ε = 36.6 (CH_3CN)[21] along with D3BJ for dispersions correction were used.[22,23] The geometry optimizations were performed using the RIJCOSX approximation (RI approximation for non-hybrid functionals).[24]

Evaluation of functionals in geometry optimizations for $[(susan^{6\text{-}Me})\{Fe^{III}(\mu\text{-}O)(\mu\text{-}O_2)Fe^{III}\}]^{2+}$ In order to evaluate geometries and molecular properties within DFT calculations, we have first tested several functionals in the geometry optimizations of $[(susan^{6\text{-}Me})\{Fe^{III}(\mu\text{-}O)(\mu\text{-}O_2)Fe^{III}\}]^{2+}$. Geometry optimizations were employed starting at the molecular structure of $[(susan^{6\text{-}Me})\{Fe^{III}(\mu\text{-}O)(\mu\text{-}O_2)Fe^{III}\}]^{2+}$ obtained from single-crystal X-ray diffraction. These geometry optimizations for a diferric complex included broken-symmetry calculations. Broken-symmetry solutions of the diiron complexes were found

by first converging to hypothetical ferromagnetically coupled high-spin solutions and then converging to a broken symmetry state using the ORCA command “brokensym”. Geometry optimizations were then conducted on the energetically lower solutions. In all cases, the broken symmetry solutions were energetically favored and persisted throughout the geometry optimization processes. The results of these calculations are summarized in Figure S2 and Table S2 including the functionals employed.

Supplementary Fig. S2. Geometry optimized molecular structures of [(susan$^{6\text{-Me}}$){Fe^{III}(μ-O)(μ-1,2-O_2)Fe^{III}}]$^{2+}$ using different functionals.

BP86

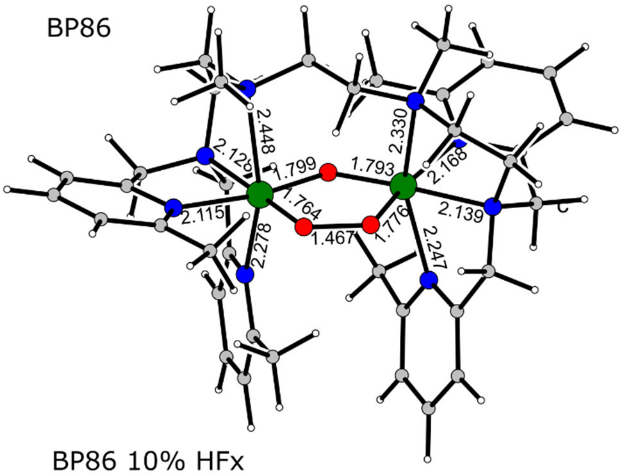

TPSS

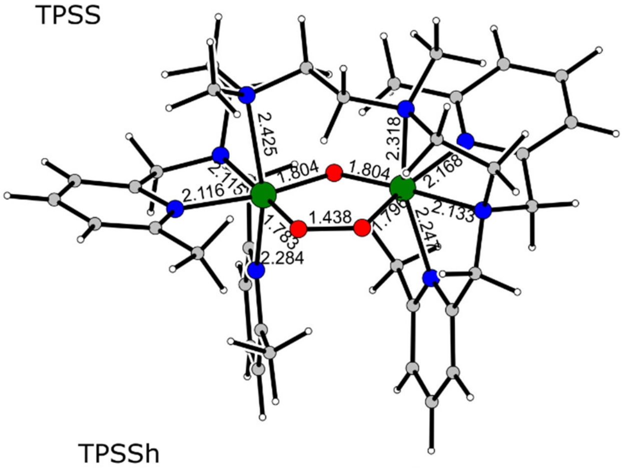

BP86 10% HFx

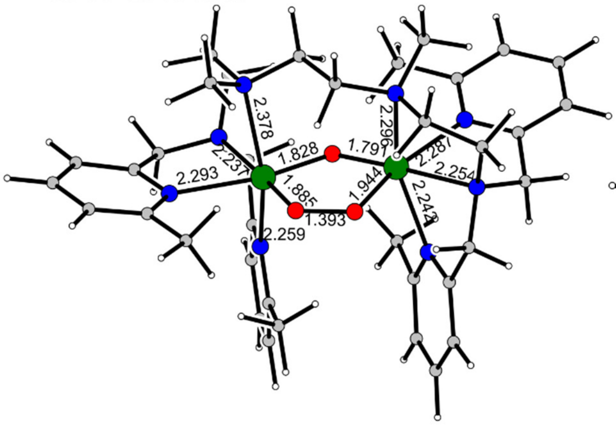

TPSSh

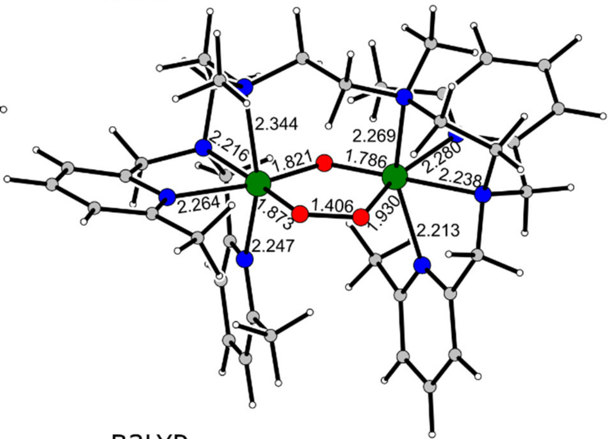

TPSSh 15% HFx

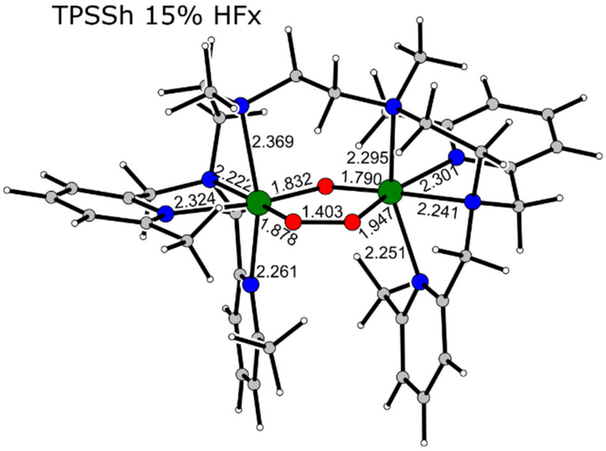

B3LYP

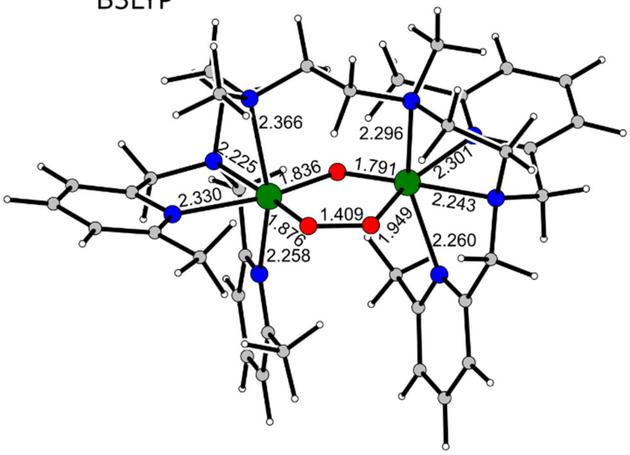

Supplementary Table 2. Selected bond lengths [Å] for $[(susan^{6\text{-}Me})\{Fe^{III}(\mu\text{-}O)(\mu\text{-}O_2)Fe^{III}\}]^{2+}$ in crystals of $[(susan^{6\text{-}Me})\{Fe^{III}(\mu\text{-}O)(\mu\text{-}O_2)Fe^{III}\}](ClO_4)_2{\bullet}0.85CH_3CN{\bullet}0.7H_2O$ or in optimized geometries with the functional given in the table (other computational details are given above)

	X-ray	BP86	TPSS	TPSSh	BP86 + 10%HFx	TPSSh + 15%HFx	B3LYP
Fe1-O1	1.875	1.764	1.783	1.873	1.885	1.878	1.876
Fe1-O3	1.824	1.799	1.804	1.821	1.828	1.832	1.836
Fe1-N1	2.344	2.448	2.425	2.344	2.378	2.369	2.366
Fe1-N2	2.184	2.128	2.116	2.216	2.237	2.222	2.225
Fe1-N3	2.237	2.278	2.284	2.247	2.259	2.261	2.258
Fe1-N4	2.313	2.115	2.115	2.264	2.293	2.324	2.330
Fe2-O2	1.928	1.776	1.796	1.930	1.944	1.947	1.949
Fe2-O3	1.790	1.793	1.804	1.786	1.791	1.790	1.791
Fe2-N41	2.288	2.330	2.318	2.269	2.296	2.295	2.296
Fe2-N42	2.214	2.139	2.168	2.238	2.254	2.241	2.243
Fe2-N43	2.214	2.247	2.247	2.213	2.242	2.251	2.260
Fe2-N44	2.283	2.168	2.133	2.280	2.287	2.301	2.301
O1-O2	1.432	1.467	1.438	1.406	1.393	1.403	1.409

While non-hybrid functionals (BP86[25,26] and TPSS[27]) results in too short $Fe\text{-}O^{peroxo}$ bonds, the inclusion of 10 % Hartree-Fock exchange improved the reproduction of the experimental molecular structure significantly. Among these functionals, the *meta*-hybrid GGA functional TPSSh[27,28] performed slightly better than BP86 with 10 % Hartree-Fock exchange added. A further increase of Hartree-Fock exchange led to further increase of Fe-O bond lengths (using either TPSSh with 15 % Hartree-Fock exchange or B3LYP[25,26] that includes 20 % Hartree-Fock exchange, Table S2). From a critical comparison of all these results to the experimental molecular structure, the TPSSh was selected for all further geometry optimizations. Mulliken spin populations of the TPSSh optimized structure of $[(susan^{6\text{-}Me})\{Fe^{III}(\mu\text{-}O)(\mu\text{-}O_2)Fe^{III}\}]^{2+}$ are provided in the upper row of Table S3.

Supplementary Table 3. Mulliken spin populations of the core atoms of the geometry optimized structures at the TPSSh/def2-TZVP level of theory

	Fe1	Fe2	O1	O2	O3
{Fe^{III}(μ-O)(μ-1,2-OO)Fe^{III}}	3.99	-3.99	0.22	-0.16	-0.04
{Fe^{III}(μ-O)(μ-1,2-OO)Fe^{IV}}	4.01	-3.01	0.14	-0.09	-0.18
{Fe^{IV}(μ-O)(μ-1,2-OO)Fe^{III}}	-2.95	4.01	-0.21	0.02	-0.05
{Fe^{III}(μ-O)(μ-1,2-HOO)Fe^{III}}	4.10	-4.05	0.02	-0.15	0.06
{Fe^{III}(μ-O)(μ-1,2-OOH)Fe^{III}}	4.09	-4.11	0.20	0.01	-0.13
{Fe^{III}(μ-OH)(μ-1,2-OO)Fe^{III}}	4.08	-4.11	0.25	-0.17	-0.03

DFT calculations on variously protonated forms of the protonated complex [(susan$^{6\text{-Me}}$){Fe^{III}(μ-O)(μ-O_2)Fe^{III}}]$^{2+}$ to evaluate the site of protonation

In order to evaluate the site of protonation, the molecular structure, and the properties of the protonated complex, a proton was added to the experimental molecular structure of [(susan$^{6\text{-Me}}$){Fe^{III}(μ-O)(μ-1,2-O_2)Fe^{III}}]$^{2+}$

1) at the peroxo oxygen atom O1 leading to the hypothetical
   μ-1,2-HOO complex [(susan$^{6\text{-Me}}$){Fe^{III}(μ-O)(μ-1,2-HOO)Fe^{III}}]$^{3+}$,
2) at the peroxo oxygen atom O2 leading to the hypothetical
   μ-1,2-OOH complex [(susan$^{6\text{-Me}}$){Fe^{III}(μ-O)(μ-1,2-OOH)Fe^{III}}]$^{3+}$, and
3) at the oxo oxygen atom O3 leading to the hypothetical
   μ-OH complex [(susan$^{6\text{-Me}}$){Fe^{III}(μ-OH)(μ-1,2-OO)Fe^{III}}]$^{3+}$.

Geometry optimizations were performed at the TPSSh/def2-TZVP level of theory (*vide supra*) and the results are shown in Figure S3. Mulliken spin populations are provided in Table S3. The final single-point energies of the optimized structure for the three different tautomers of the protonated species provided the μ-oxo-O3 protonated species lowest in energy while the μ-peroxo-O2 protonated species is 76 cm^{-1} (0.22 kcal mol^{-1}) higher in energy and the μ-peroxo-O1 protonated species 1200 cm^{-1} (3.43 kcal mol^{-1}) higher in energy. While the energy difference of 0.22 kcal mol^{-1} is significantly below the accuracy of the method (2-3 kcal mol^{-1}), the energy difference between the μ-peroxo-O2 protonated species and the μ-peroxo-O1 protonated species of 3.21 kcal mol^{-1} is above this

accuracy.

Supplementary Fig. 3. Experimental molecular structure of [(susan$^{6\text{-Me}}$){Fe^{III}(μ-O)(μ-1,2-O$_2$)Fe^{III}}]$^{2+}$ from single-crystal x-ray diffraction (top center). Geometry optimized molecular structures of the three different tautomers of the protonated complex: middle left protonation at the peroxo oxygen atom O1, middle right protonation at the peroxo oxygen O2, bottom protonated at the oxo oxygen atom O3.

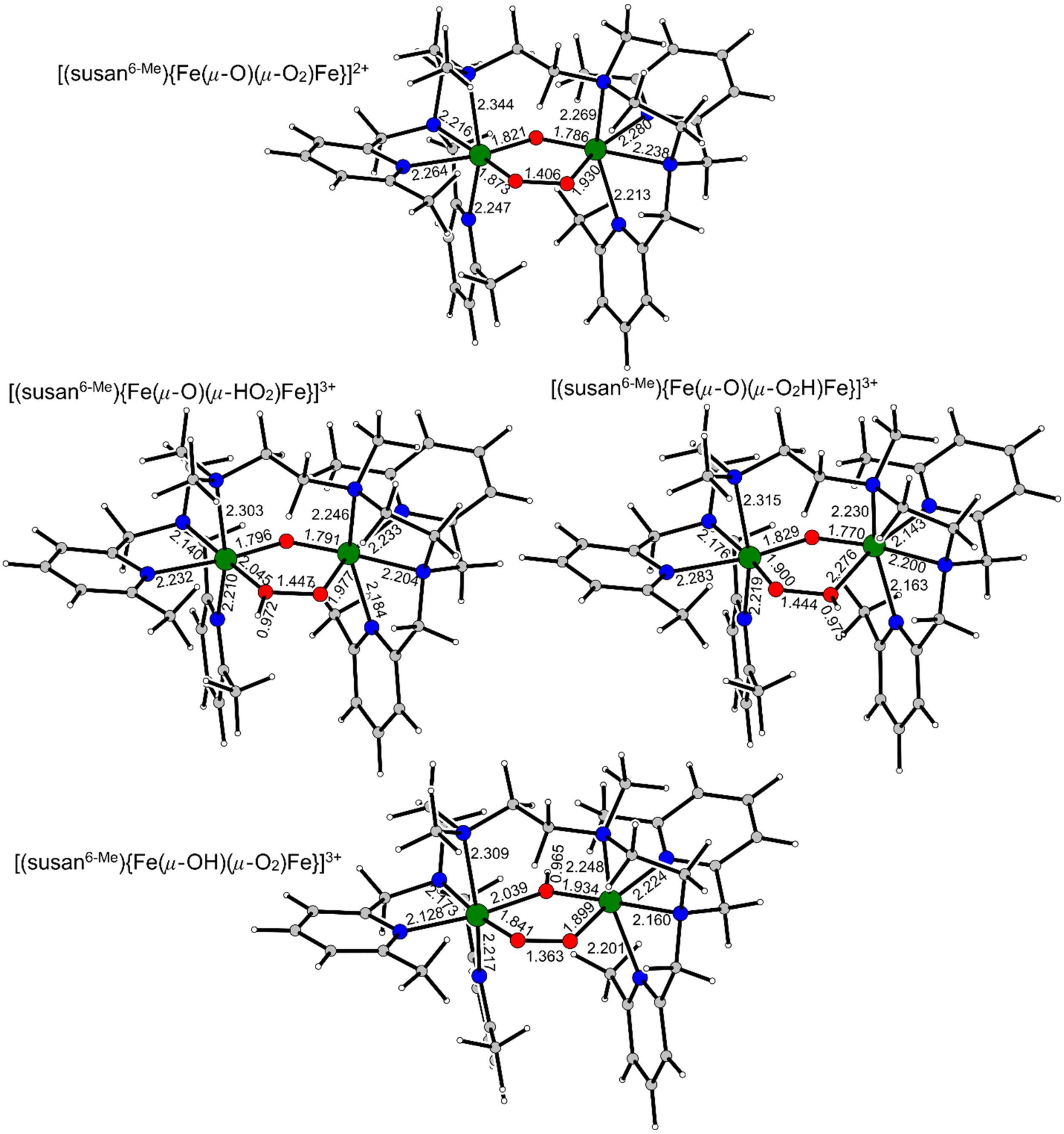

DFT calculations on the oxidized complex [(susan$^{6\text{-}Me}$){Fe(μ-O)(μ-O_2)Fe}]$^{3+}$ to better understand the experimental Mössbauer parameters

Calculations for the oxidized complex started from the experimental molecular structure of [(susan$^{6\text{-}Me}$){Fe^{III}(μ-O)(μ-1,2-O_2)Fe^{III}}]$^{2+}$. In the ORCA input, the total charge was increased to 3+ and the spin multiplicity of the hypothetical ferromagnetically coupled high-spin solution was set to 10. In order to optimize the geometry of either Fe1 being oxidized to Fe^{IV} (Fe^{IV}1Fe^{III}2 configuration) or Fe2 being oxidized to Fe^{IV} (Fe^{III}1Fe^{IV}2 configuration), the ORCA “Brokensym 5,4” command was applied for the order Fe1Fe2 and Fe2Fe1, respectively. Geometry optimizations were performed at the TPSSh/def2-TZVP level of theory (*vide supra*) and the molecular structures obtained are shown in Figure S4.

Supplementary Fig. 4. Experimental molecular structure of [(susan$^{6\text{-}Me}$){Fe^{III}(μ-O)(μ-1,2-O_2)Fe^{III}}]$^{2+}$ from single-crystal x-ray diffraction (top center). Geometry optimized molecular structures for the oxidized complex: bottom left Fe1 oxidized to Fe^{IV} (Fe^{IV}1Fe^{III}2 configuration) and bottom right Fe2 oxidized to Fe^{IV} (Fe^{III}1Fe^{IV}2 configuration).

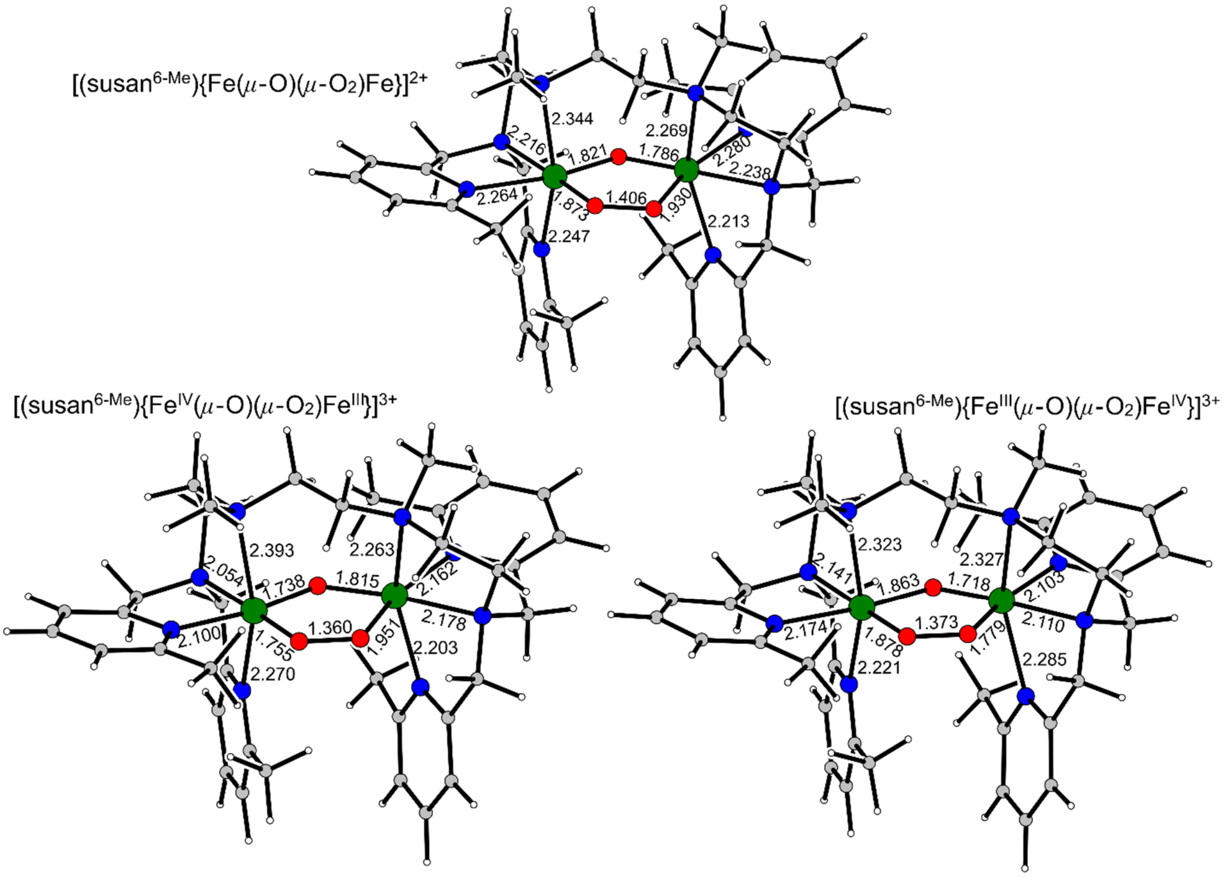

The Mulliken spin populations (Table S3) provide relatively clear values of ±4.0 for Fe^{III} h.s. ions in the diferric complexes. These values for Fe^{III} h.s. are only marginally influenced upon oxidation of the second iron ion. The latter exhibits Mulliken spin populations of -3.0 consistent with Fe^{IV} h.s. coupled antiferromagnetically to the majority spin Fe^{III} h.s. These Mulliken spin populations confirm that the geometry optimizations and SCF cycles converged to the $Fe^{IV}1Fe^{III}2$ and $Fe^{III}1Fe^{IV}2$ configurations. The final single-point energy of $Fe^{IV}1Fe^{III}2$ was found to be 820 cm^{-1} (2.34 kcal mol^{-1}) lower in energy, which is in the error range of these calculations. These results indicate a localized $Fe^{IV}Fe^{III}$ class II description. However, the energy difference between the two configurations only reflect the intrinsic difference between the two coordination sites. In this respect it favors an energetic preference for one configuration without significant thermal population up to room temperature, while the level of theory is not sufficient to assign the lower energy configuration to the $Fe^{IV}1Fe^{III}2$ configuration despite it was found to be lower by these DFT calculations.

Calculation of Mössbauer parameters. DFT calculations of the Mössbauer parameters (the isomer shift δ and the quadrupole splitting ΔE_Q) were performed on the broken symmetry solutions of the geometry optimized structures (*vide supra*) with an established protocol at the B3LYP CP(PPP)/def2-TZVP level of theory[29,30] with only minor modifications. Due to the relative large size of the complexes and expectation of only non-significant variations we changed the basis set of carbon and hydrogen atoms to def2-SVP - as it was reported previously.[31] The calculation of the isomer shift is then based on applying a linear correlation between experimentally observed isomer shifts and calculated total electron densities at the iron nuclei obtained for a series of complexes. Using this protocol with the published correlation resulted in a relatively poor reproduction of the isomer shifts of $[(susan^{6-Me})\{Fe^{III}(\mu\text{-}O)(\mu\text{-}O_2)Fe^{III}\}]^{2+}$ and $[(susan^{6-Me})\{Fe^{II}(\mu\text{-}OH)_2Fe^{II}\}]^{2+}$. Applying the same methodology not to the geometry optimized structures

but to the experimentally determined molecular structures provided no improvement.

In order to get more insight into these effects, we calculated δ and ΔE_Q for all other diiron complexes of the ligand susan$^{6\text{-}Me}$ yet obtained (two diferrous, one diferric, and one mixed-valence $Fe^{II}Fe^{III}$ complex) based on their crystallographically determined molecular structures. All calculated isomer shifts underestimate the experimental values roughly by 0.1 mm s^{-1}, while ΔE_Q were calculated within the known limited accuracy. For prediction of ΔE_Q we tested in some cases a different protocol[32] but observed only small effects. Therefore, ΔE_Q was calculated at the B3LYP/CP(PPP) level of theory.[18] However, to gain more accuracy in the computation of δ we decided to use a linear dependence between the calculated total electron densities at the iron nuclei ρ and the experimental isomer shifts, which is adapted to the diiron complexes of the ligand susan$^{6\text{-}Me}$. In order to complement the linear dependence to higher valent cases, we incorporated six additional mononuclear iron complexes (Table S4).

Supplementary Table 4. Experimental isomer shifts and calculated total electron densities at the iron nuclei at B3LYP CP(PPP)/def2-TZVP level of theory[29,30] for the complexes used in establishing a correlation more specific for the susan^{6-Me} complexes.

	δ^{exp} / mm s^{-1}	ρ_{Fe1} - 11810	δ^{calc}_{Fe1} / mm s^{-1}	ρ_{Fe2} - 11810	δ^{calc}_{Fe2} / mm s^{-1}	Lit.
[(susan^{6-Me}){Fe^{II}(μ-F)$_2$Fe^{II}]$^{2+}$	1.15	5.10	1.16	5.11	1.15	1
[(susan^{6-Me}){Fe^{II}F(μ-F)Fe^{III}F]$^{2+}$	1.13<br>0.47	5.16	1.13	 6.70	 0.51	1
[(susan^{6-Me}){Fe^{II}F(μ-F)Fe^{II}F]$^{+}$	1.20	5.08	1.16	5.09	1.16	33
[(susan^{6-Me}){Fe^{II}(μ-OH)$_2$Fe^{II}]$^{2+}$	1.13	5.16	1.13	5.18	1.12	
[(susan^{6-Me}){Fe^{III}(μ-O)(μ-O$_2$)Fe^{III}]$^{2+}$	0.53	6.58	0.56	6.65	0.53	
[(susan^{6-Me}){Fe^{III}F(μ-O)Fe^{III}F]$^{2+}$	0.47	6.67	0.52	6.70	0.51	34
[Fe^{II}(TPpivP)(OAc)]$^{-}$ [a]	1.05	5.40	1.04			31,35
[Fe^{III}Cl(η^4-Mac*)]$^{-}$ [b]	0.25	7.20	0.31			31,36
[Fe^{IV}Cl(η^4-Mac*)]$^{-}$ [b]	-0.04	7.87	0.03			31,36
[Fe^{IV}(O)(TMP)]$^{+}$ [c)]	0.08	7.86	0.04			31,35
[Fe(CO)$_5$]	0.00	8.21	-0.10			30
[FeO$_4$]$^{2-}$	-0.87	2.17	-0.89			29

a H$_2$TPpivP = *meso*-tetrakis(α,α,α,α-*o*-pivalamidophenyl)porphyrin[35]

b MAC* = 1,4,8,11-tetraaza-13,13-diethyl-2,2,5,5,7,7,10,10-octamethyl-3,6,9,12,14-pentaoxocyclotetradecane[36]

c TMP = Tetramesitylphorphyrinato[35]

The calibration parameters α and β in the equation $\delta = \alpha(\rho - C) + \beta$ with C being fixed were fitted to experimental isomer shifts and computed electron densities ρ. This resulted in the following linear correlation: $\delta = -0.405 * (\rho - 11810) + 3.223$. The resulting predictions of the isomer shifts of the iron complexes of susan^{6-Me} showed excellent agreements with the experimental data with an error range of $\Delta\delta < 0.1$ mm s^{-1} (Fig. S5).

Supplementary Fig. 5. Linear dependence of the experimental isomer shifts δ with the computed electron densities ρ. Blue points represent diiron complexes of the ligand susan$^{6\text{-Me}}$ while the black points are iron complexes adapted from a benchmark test[31] and where used to complement the linear dependence to higher valent cases. This resulted in the linear correlation shown as the red line. For comparison, the blue line represent the published correlation[29,30] that were initially used to calculation the isomer shifts.

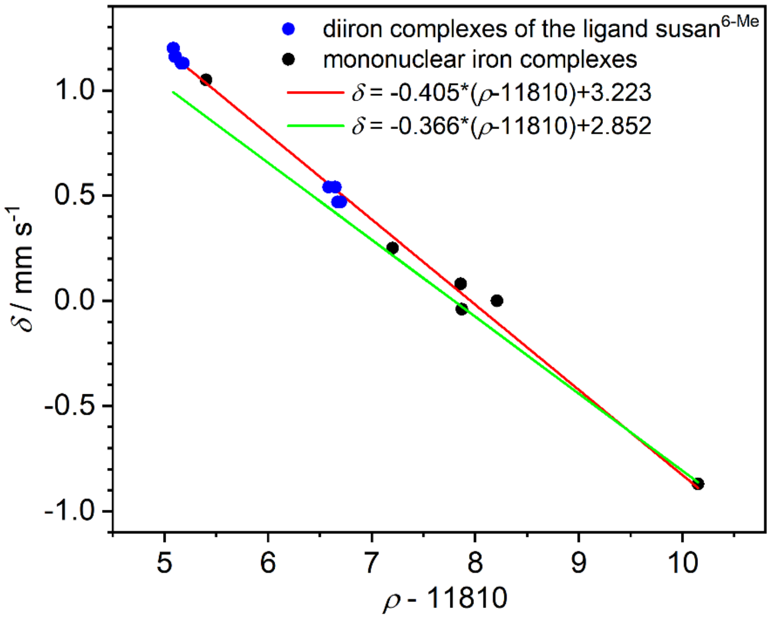

Using this methodology for the TPPSh optimized structure of [(susan$^{6\text{-Me}}$){Fe^{III}(μ-1,2-O)(μ-O$_2$)Fe^{III}}]$^{2+}$ Mössbauer parameters were calculated as δ_1 = 0.53, ΔE_{Q1} = -1.45; δ_2 = 0.58, ΔE_{Q2} = -1.28 (experimental δ_1 = δ_2 = 0.53, $|\Delta E_{Q1}|$ = $|\Delta E_{Q2}|$ = 1.68) demonstrating the overall performance of this protocol and its limitations. The Mössbauer parameters calculated for different oxidized and protonated complexes are provided in Table 1 of the main manuscript.

As the stated above, the Mulliken spin populations (Table S3) calculated with the TPSSh/def2-TZVP level of theory for the geometry optimizations provided not only clear values of ±4.0 for Fe^{III} h.s. ions in the diferric complexes but also clear values of -3.0 for Fe^{IV} h.s. in the oxidized complex confirming localized class II mixed valence configurations Fe^{IV}1Fe^{III}2 or Fe^{III}1Fe^{IV}2. In contradiction to this localized description, the experimental Mössbauer isomer shifts clearly show that both Fe^{III} ions are significantly affected by the one-electron oxidation. In this respect, it is remarkable that the DFT

calculations reproduce the significant decrease of the isomer shift of both iron ions. However, these Mössbauer parameters were calculated using a well-established protocol based the B3LYP CP(PPP)/def2-TZVP level of theory.[29,30] Interestingly, these calculations provided Mulliken spin populations (Table S5) that change only minorly to those of the TPSSh/def2-TZVP calculations thus also indicating a localized mixed valence class II $Fe^{IV}Fe^{III}$ description. This at first glance conflicting results - localized from Mulliken spin populations *vs* a description from isomer shifts in which both iron atoms are effected by oxidation - reflects the difference between spin densities and total electron densities at the nuclei. Moreover, these results indicate a relaxation of the electron density of the whole molecule to compensate for the creation of one electron hole.

Supplementary Table 5. Mulliken spin populations of the core atoms of the geometry optimized structures at the B3LYP/CP(PPP)/def2-TZVP level of theory

	Fe1	Fe2	O1	O2	O3
$\{Fe^{III}(\mu\text{-O})(\mu\text{-1,2-OO})Fe^{III}\}$	4.06	-4.07	0.24	-0.19	-0.04
$\{Fe^{III}(\mu\text{-O})(\mu\text{-1,2-OO})Fe^{IV}\}$	4.11	-3.17	0.15	-0.08	-0.18
$\{Fe^{IV}(\mu\text{-O})(\mu\text{-1,2-OO})Fe^{III}\}$	-3.04	4.13	-0.26	-0.02	-0.03
$\{Fe^{III}(\mu\text{-O})(\mu\text{-1,2-HOO})Fe^{III}\}$	4.16	-4.14	0.02	-0.13	0.08
$\{Fe^{III}(\mu\text{-O})(\mu\text{-1,2-OOH})Fe^{III}\}$	4.16	-4.16	0.18	0.00	-0.14
$\{Fe^{III}(\mu\text{-OH})(\mu\text{-1,2-OO})Fe^{III}\}$	4.12	-4.17	0.28	-0.19	-0.03

Supplementary Fig. 6. Mössbauer spectrum of [(susan$^{6\text{-Me}}$){Fe^{II}(μ-OH)$_2$$Fe^{II}$}]($ClO_4$)$_2$ at 80 K.

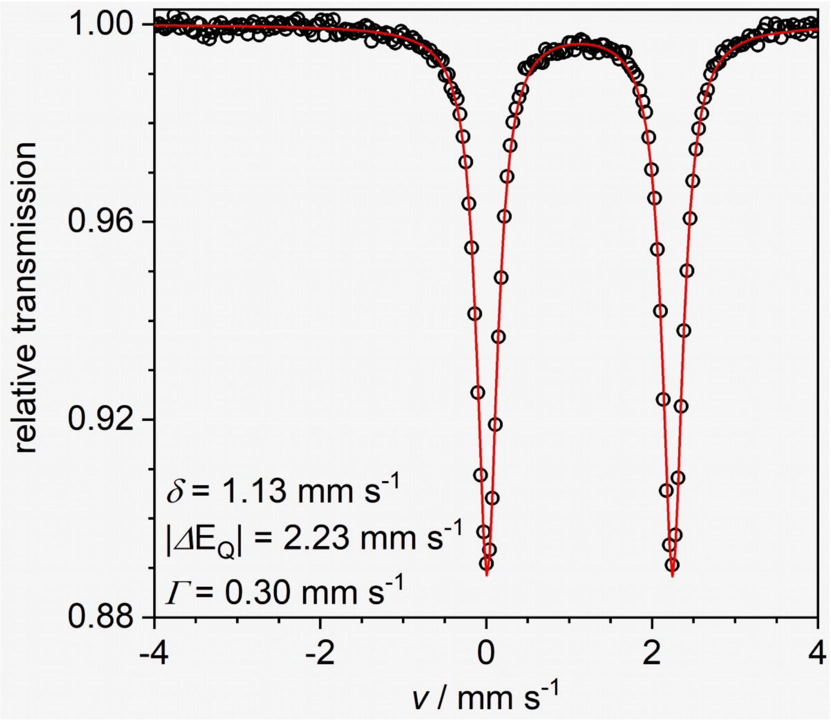

Supplementary Fig. 7. Mössbauer spectra of [(susan$^{6\text{-}Me}$){Fe^{III}(μ-O)(μ-1,2-O_2)Fe^{III}}]-(ClO_4)$_2$ at 200 K and 80 K as solids and at 80 K on a frozen solution in CH_3CN.

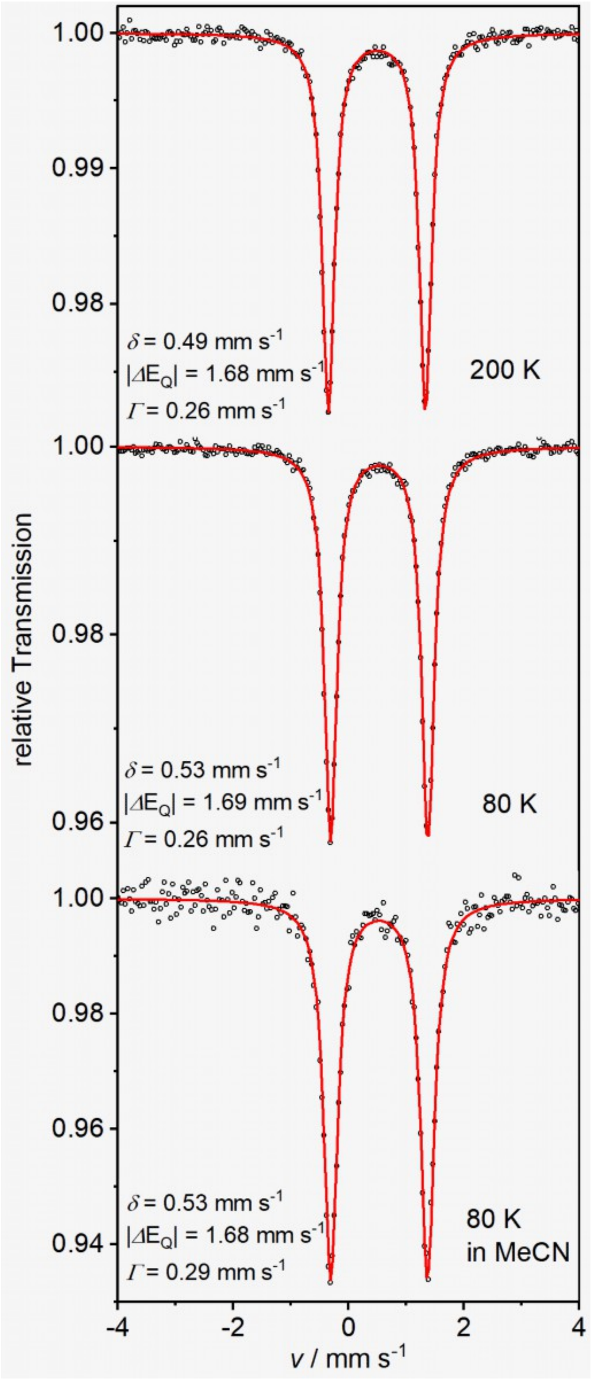

Supplementary Fig. 8. ESI-MS of the four isotopomers of [(susan$^{6\text{-}Me}$){Fe^{III}(μ-O)(μ-1,2-O_2)Fe^{III}}]$(ClO_4)_2$ as indicated it the figure.

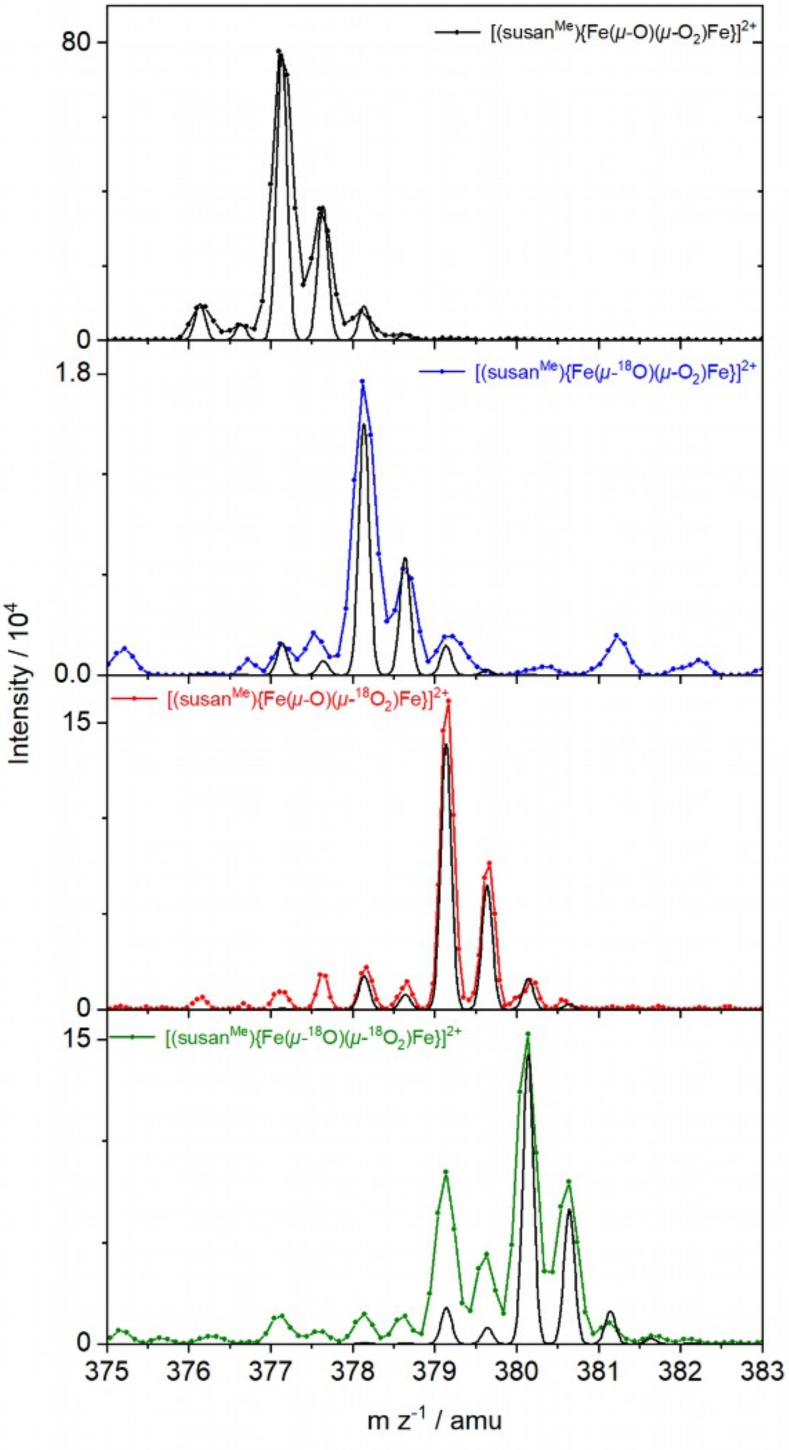

Supplementary Fig. 9. Resonance Raman spectrum of [(susan$^{6\text{-Me}}$){Fe^{III}(μ-O)(μ-1,2-O_2)Fe^{III}}]$^{2+}$ in CH_3CN (20 mM): Bands with an asterisk are from the solvent.

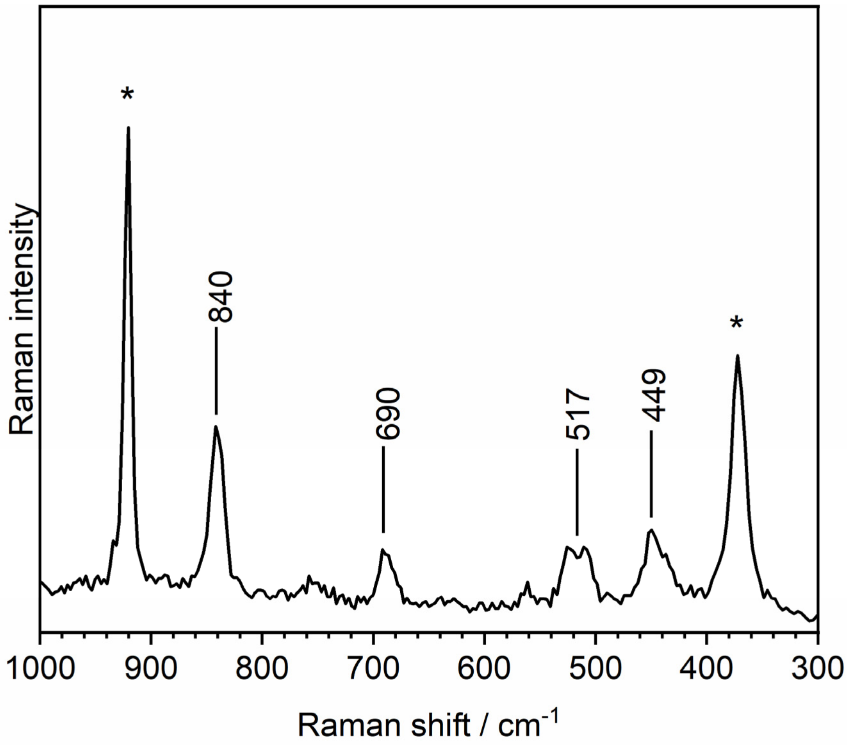

Supplementary Fig. 10. Stabilities in CH_3CN solution at -40 °C. Time traces for selected energies in the UV-Vis spectra of parent $[(susan^{6\text{-}Me})\{Fe^{III}(\mu\text{-}O)(\mu\text{-}1,2\text{-}O_2)Fe^{III}\}]^{2+}$ (top), chemically oxidized $[(susan^{6\text{-}Me})\{Fe^{IV}(\mu\text{-}O)(\mu\text{-}1,2\text{-}O_2)Fe^{III}\}]^{3+}$ (middle), and protonated $[(susan^{6\text{-}Me})\{Fe(\mu\text{-}O)(\mu\text{-}OOH)Fe\}]^{3+}$ (bottom). The solid lines are fits with one exponential decay function providing estimates for the half-lives of 90 min and 11 min for the oxidized and protonated species, respectively.

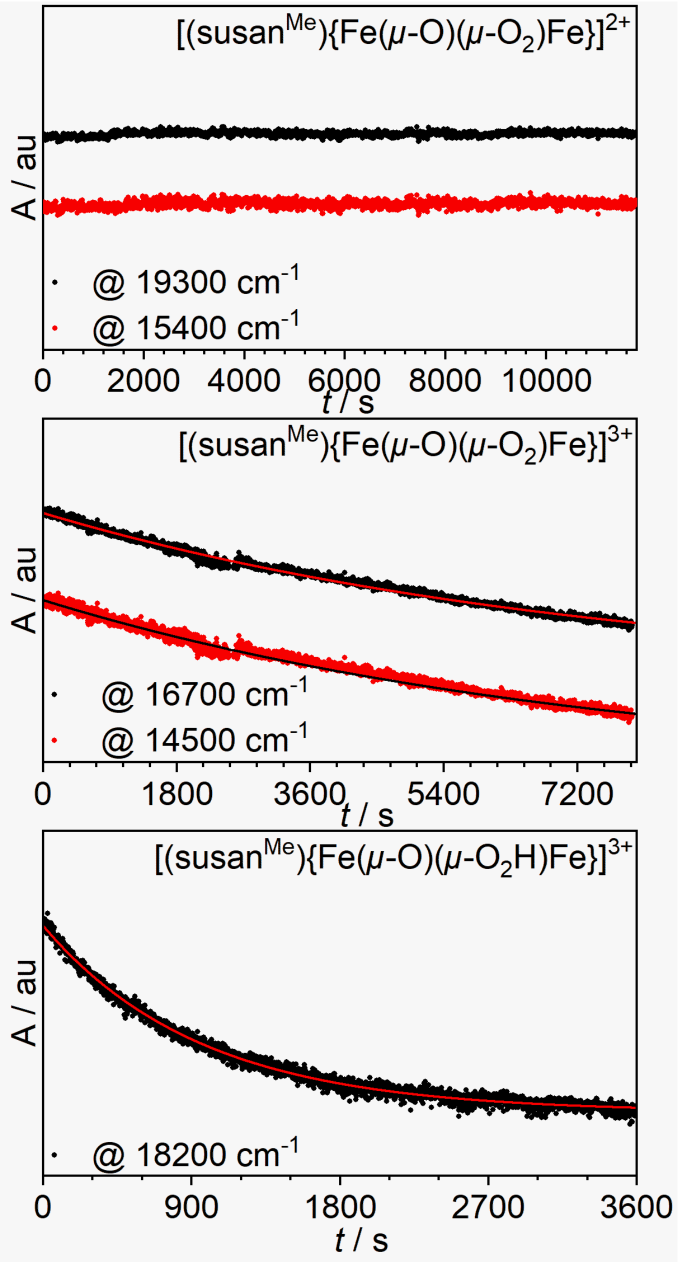

Supplementary Fig. 11. Gaussian analysis of the UV-Vis spectra of [(susan$^{6\text{-}Me}$){Fe^{III}(μ-O)(μ-O_2)Fe^{III}}]$^{2+}$ and the electrochemically generated oxidized species [(susan$^{6\text{-}Me}$){Fe^{IV}(μ-O)(μ-O_2)Fe^{III}}]$^{3+}$ both in CH_3CN at -40 °C.

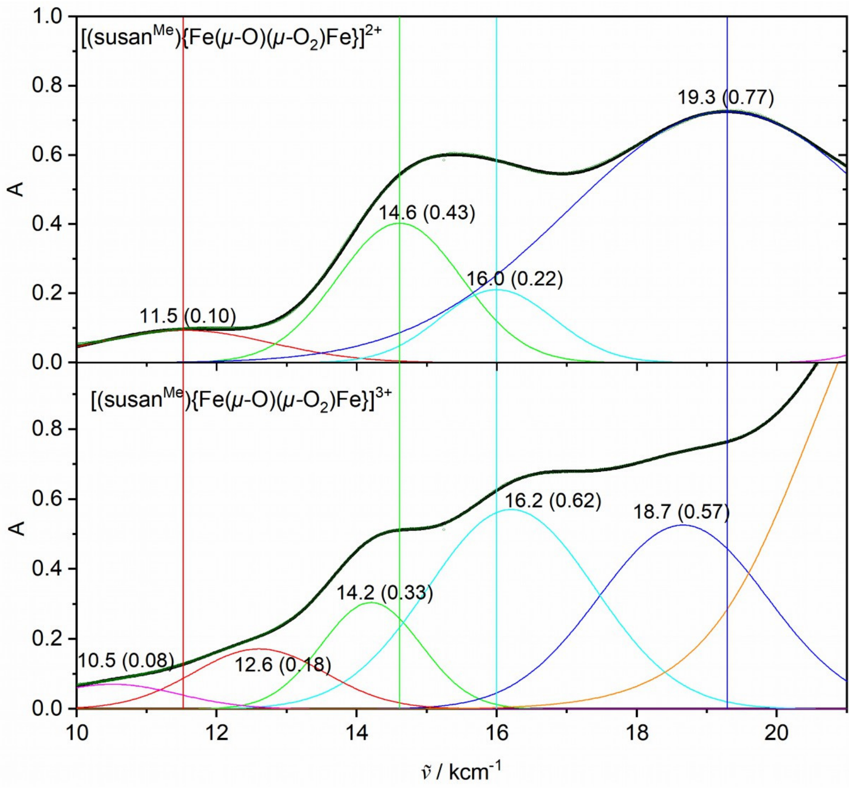

Supplementary Fig. 12. a) *Q vs t* dependence for the potentiostatic coulometry at 0.68 V *vs* Fc^+/Fc and b) time traces for selected energies of the corresponding spectroelectrochemistry. c) *Q vs t* dependence for the potentiostatic coulometry at 0.16 V *vs* Fc^+/Fc and d) time traces for selected energies of the corresponding spectroelectrochemistry.

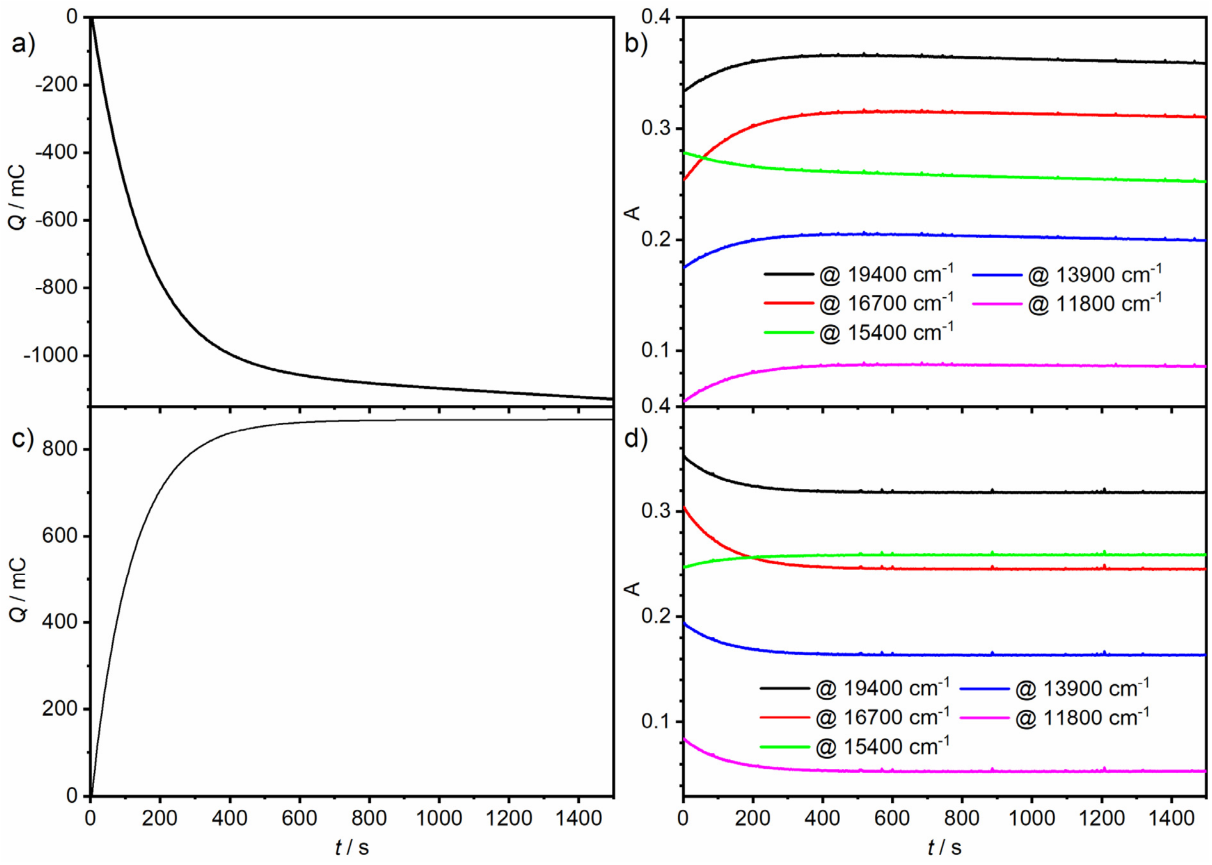

Supplementary Fig. 13. EPR spectrum (top) of chemically oxidized [(susan$^{6\text{-Me}}$){Fe(μ-O)(μ-O_2)Fe}]$^{3+}$ in ca. 0.4 mM CH_3CN solution at 10 K (top, 9.63312 GHz, 80 μW power, 0.75 mT modulation), and its simulation (red trace) with two subspectra (green and blue), and the numerically integrated EPR spectrum (bottom) with simulations of both species. Note the small contribution of the isotropic S = ½ impurity species in the absorption pattern. The simulation is obtained with two S = ½ subspectra with g values **g**(1) = (2.2724, 2.1523, 2.0212), 99.3% rel. intensity, green trace, and a minor isotropic species with **g**(2) = (1.9919, 2.0, 2.0) of 0.7 %, blue trace. The line shapes were Gaussian and the line width (full width half maximum) were w(1) = (21.0, 13.0, 2.5) mT and w(2) = 2.5 mT isotropic.

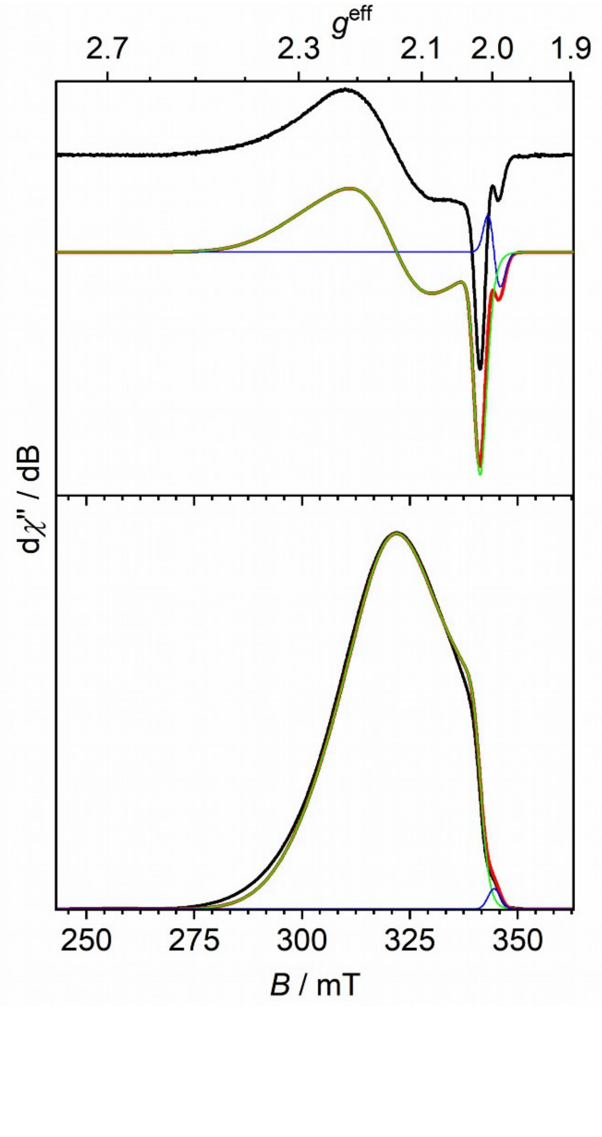

Supplementary Fig. 14. Zero-field Mössbauer spectra of chemically oxidized [(susan^{6-Me}){Fe^{III}(μ-O)(μ-O_2)Fe^{IV}}]$^{3+}$ in frozen CH_3CN solution. Mössbauer spectra at 180 K (top) and 80 K (bottom) on the left for nested models (1) of subspectra and right for stacked models (2). The parameters obtained from fits with Lorentzian doublets in mm s^{-1} are given on the plots. Because of the seemingly different broadening of the center and the wings of the spectrum at 80 K (and below, see Fig. S18) we favor the nested model (1) (lower left panel) over the stacked model (2) (lower right panel). Correspondingly, stacking of doublets leads to distinctly lower quality of the fit. This conclusion is also supported by the applied field Mössbauer spectra of Figure S15.

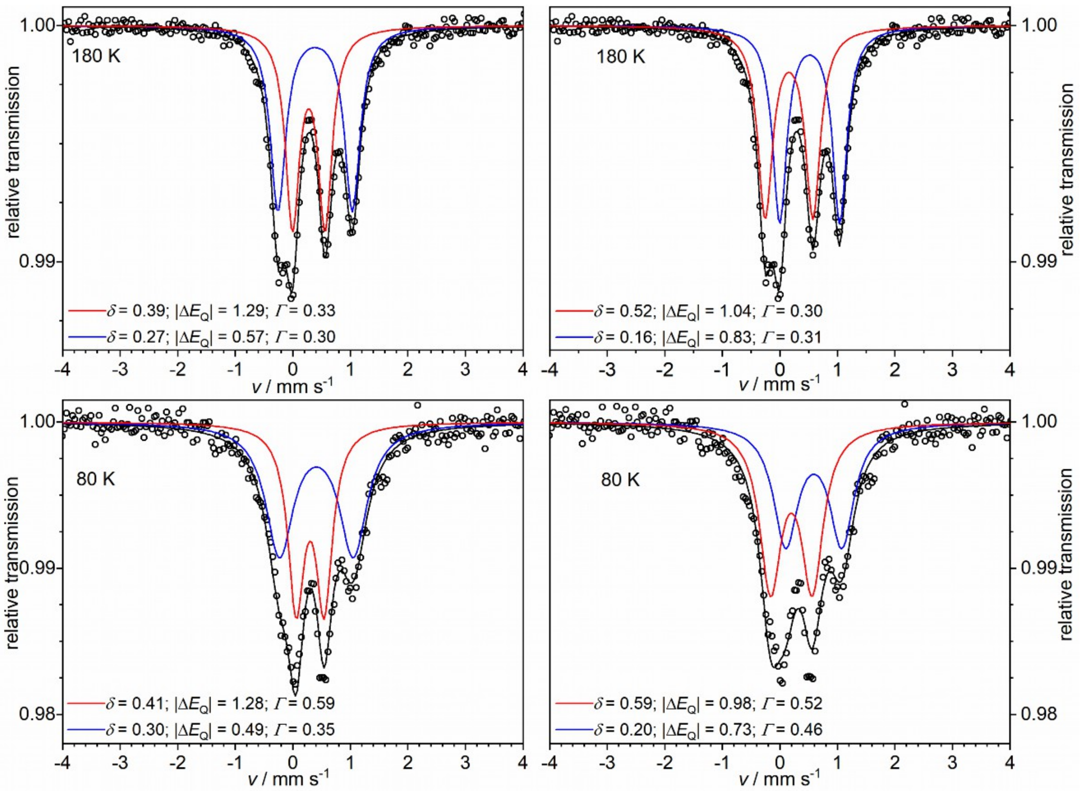

Since the isomer shift δ is a key parameter for oxidation state assignments, the values can be used for an interpretation of the two models in terms of localized/delocalized valences. The larger difference in isomer shifts for the stacked model (2), $\Delta\delta$ = 0.36 mm s^{-1} *vs* $\Delta\delta$ = 0.18 mms^{-1} found for the nested model (1), suggests more localized Fe^{III} and Fe^{IV} sites for model (2) whereas model (1) would be rather consistent with a more not-localized (either delocalized class III or localized class II with fast hopping) $Fe^{III}Fe^{IV}$ mixed valence species. The different quadrupole splitting found for the nested model (1) may be due to different local symmetries. Details of localized and not-localized model considerations are given in Figure S17.

Supplementary Fig. 15. Magnetic Mössbauer spectra of the mixed-valence complex $[(\text{susan}^{6\text{-Me}})\{\text{Fe}^{\text{III}}(\mu\text{-O})(\mu\text{-O}_2)\text{Fe}^{\text{IV}}\}]^{3+}$ obtained by chemical oxidation in frozen CH_3CN solution measured at 1.7 K with fields of 4 T (top row) and 1 T (bottom row) applied perpendicular to the γ rays.

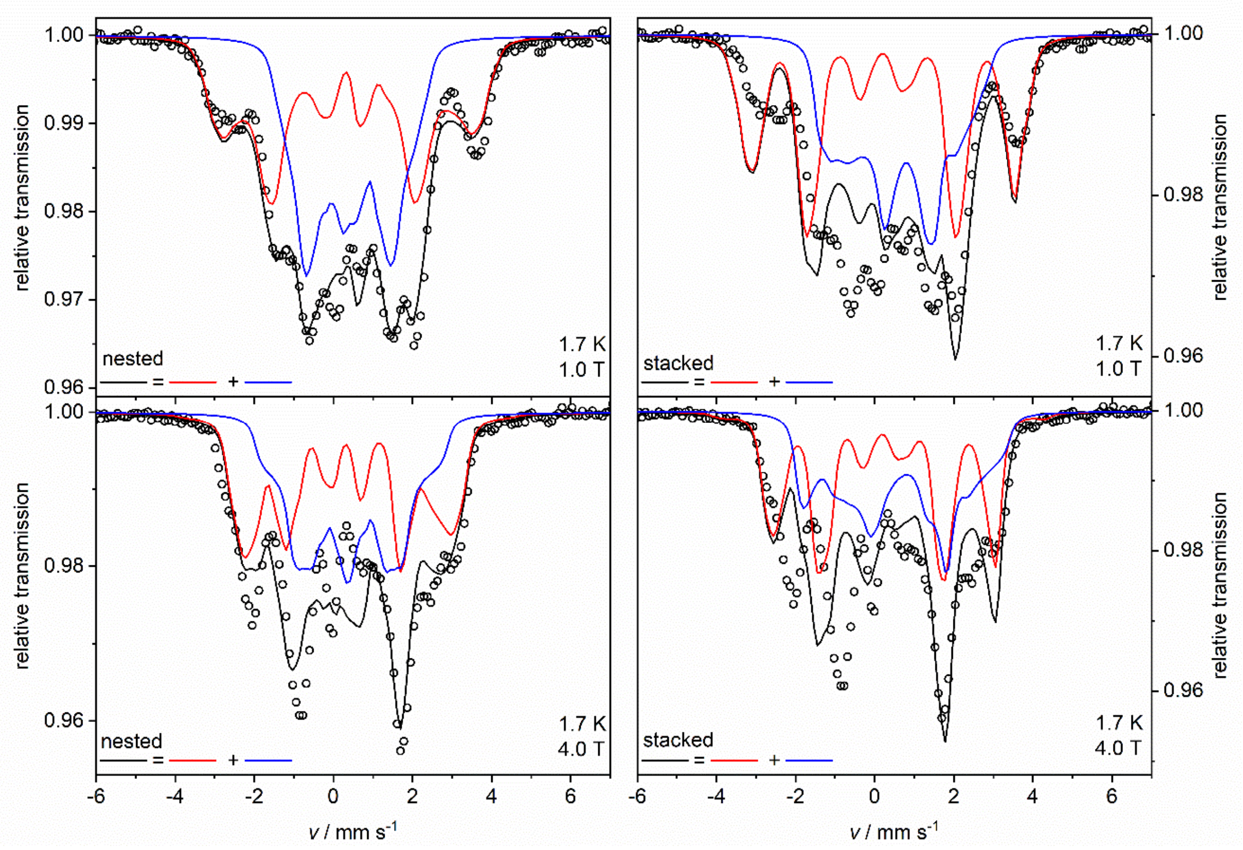

Only at base temperature (1.7 K) the data could be reasonably well analyzed by using the usual spin-Hamiltonian formalism for the cluster spin ground state S_t = 1/2, since even at 5 K the spectra showed significant broadening due to the onset of intermediate paramagnetic relaxation (Fig. S18). The Zeeman *g* values **g** = (2.02, 2.14, 2.27) were taken from EPR, and two subspectra of equal intensities were adopted with either the nested model (1) of isomer shifts and quadrupole splitting (left panel), or the stacked model (2) (right panel). Isomer shifts and quadrupole splittings were taken from the zero-field spectra shown in Figure S14 and slightly adapted for the lower temperatures. The signs of ΔE_Q and the asymmetry parameters η were optimized, as well as the hyperfine coupling tensors for both iron sites, $A/g_N\beta_N$, given in Tesla. For the sake of less ambiguity all tensor axes have been taken to coincide. The parameters used are provided in Supplementary Table 6.

Supplementary Table 6. Fit pararmeters for the magnetic Mössbauer spectra of the mixed-valence complex $[(susan^{6-Me})\{Fe^{III}(\mu\text{-}O)(\mu\text{-}O_2)Fe^{IV}\}]^{3+}$.

	nested site1	nested site 2	stacked site 1	stacked site 2
δ / mm s^{-1}	0.31	0.42	0.21	0.60
ΔE_Q / mm s^{-1}	0.50	-1.32	-0.75	-1.00
η	0.4	0.8	0.71	0.07
$A_{xx}/g_N\beta_N$ / T	-43.3	-5.1	-42.	+23.1
$A_{yy}/g_N\beta_N$ / T	-43.3	-4.3	-42.	+2.6
$A_{zz}/g_N\beta_N$ / T	-16.2	+19.8	-41.	+17.9
$A'_{xx}/g_N\beta_N$ / T [a)]	-18.6	+3.8	-18.	-17.3
$A'_{yy}/g_N\beta_N$ / T [a)]	-18.6	+3.2	-18.	-2.0
$A'_{zz}/g_N\beta_N$ / T [a)]	-6.9	-14.9	-17.6	-13.4

[a)] Values with respect to local spin S_1 = 5/2, S_2 = 2, obtained by using the spin projection expressions for antiferromagnetic coupling, $A'(1) = 3/7\ A(1)$, and $A'(2) = -3/4\ A(2)$

In both models, nested and stacked, the average *A* values for site 1 with respect to the cluster spin are negative, as expected for the majority spin S_1 = 5/2, if site 1 is high-spin Fe^{III}. (For iron the intrinsically negative Fermi contact contribution to the internal field is usually dominantly strong and determines the sign of the average *A* values, which is the trace of the **A** tensor.) In contrast, the average *A* values for site 2 are positive, as expected for an antiferromagnetically coupled minority spin S_2 = 2, here formally representing high-spin Fe^{IV}. The field dependence of the outer lines of the two experimental spectra, moving inward for a stronger applied field, reveals negative *A* values for the corresponding subspectrum. Hence, this component should be assigned to the majority spin, *i.e.* high spin Fe^{III} (simulated by the red traces). The fact that the corresponding fit for the low isomer shift of 0.21 mm s^{-1} for site 1 in the stacked model shows systematic deviations in this distinct region of the complex spectra, whereas the higher isomer shift for the nested model copes much better, is another rational argument for the nested model, as already argued from the temperature-dependent line broadening of zero-field spectra in Figure S14.

Supplementary Fig. 16. ^{57}Fe Mössbauer spectra at 80 K of a) [(susan$^{6\text{-Me}}$){Fe^{III}(μ-O)(μ-O$_2$)Fe^{IV}}]$^{3+}$ oxidized with (thia)ClO$_4$ in CH$_3$CN at -40 °C and b) after adding a drop of NEt$_3$ resulting in the re-reduction to [(susan$^{6\text{-Me}}$){Fe^{III}(μ-O)$_2$(μ-O$_2$)Fe^{III}}]$^{2+}$.

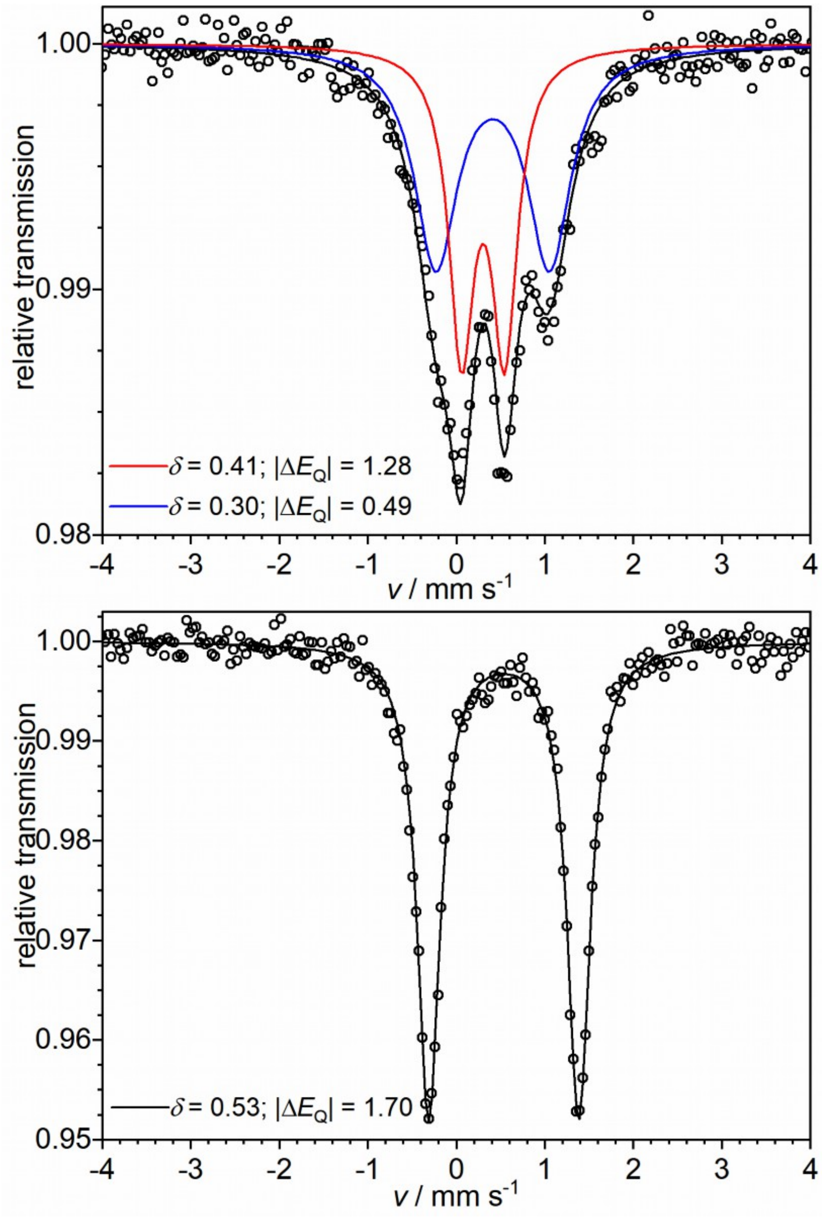

Supplementary Fig. 17. a) Mössbauer spectra for a class II mixed valence system[37] of equivalent iron sites A = B. b) Mössbauer spectra for a class II mixed valence systems with two inequivalent iron sites A ≠ B.

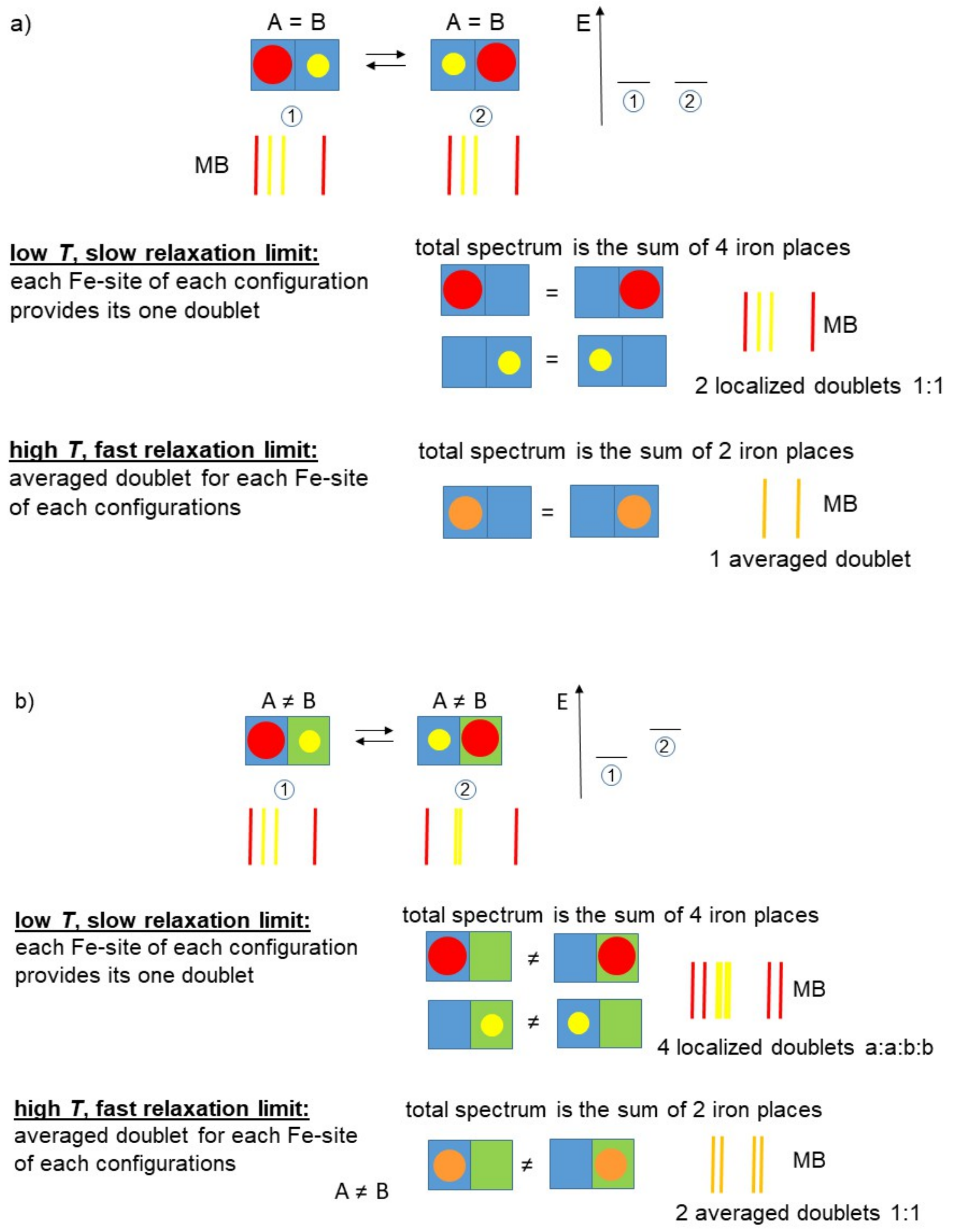

In a class II mixed valence system with equivalent sites A and B, the excess electron can be on each site. The site with the excess electron (reduced site) is symbolized by the larger red sphere in contrast to the oxidized site symbolized by the smaller yellow sphere. The two possible electron configurations are labeled by ① and ②. The electron configurations ① with the excess electron at site A and ② with the excess electron at site B have the same energy. For both localized configurations, each site provides an own

Mössbauer doublet indicated by the Mössbauer stick spectra with a red doublet for the reduced site and a yellow doublet for the oxidized site.
In the slow relaxation limit at low temperature, each site of each configuration provides its own Mössbauer doublet. Due to symmetry, the spectra of the reduced site on A and on B are the same. Thus, the overall spectrum is the sum of two localized doublets.
In the fast relaxation limit at high temperature, the electron hopping is faster than the Mössbauer time scale of 10^{-7} s, so that for each site an averaged Mössbauer spectrum is observed. Due to symmetry, the two sites A and B are identical so that the overall spectrum is one doublet with averaged Mössbauer parameters.
Such a temperature-dependent electron hopping was observed in a symmetrical class II system of Fe^{II} low spin and Fe^{III} low spin with two localized doublets at 5 K and one averaged doublet at 353 K.[38]

In a class II mixed valence system with two inequivalent sites A and B (symbolized by blue and green coordination sites), the excess electron can be on each site. The two possible electron configurations ① with the excess electron at site A and ② with the excess electron at site B do have different energies. For both localized configurations, each site provides an own Mössbauer doublet indicated by the Mössbauer stick spectra with a red doublet for the reduced site and a yellow doublet for the oxidized site.
In the slow relaxation limit at low temperature, each site of each configuration provides its own Mössbauer doublet. Due to asymmetry, all four spectra are different. Due to the low temperature, the configuration with the lower energy is more populated (here ①). Thus, the overall spectrum is the sum of four localized doublets with the two doublets of ① with the higher ratio a and the doublets of the energetically higher ② with the smaller ratio b.
In the fast relaxation limit at high temperature, the electron hopping is faster than the Mössbauer time scale of 10^{-7} s, so that for each site an averaged Mössbauer spectrum is observed. Due to asymmetry, the two sites A and B are not identical so that the overall spectrum consists of two doublets with averaged Mössbauer parameters. Such an asymmetrical class II system might be the case for $[(\text{susan}^{6\text{-Me}})\{Fe^{III}(\mu\text{-O})(\mu\text{-}O_2)Fe^{IV}\}]^{3+}$. At 180 K, the system is in the fast hopping limit and two different averaged Mössbauer doublets are observed. At lower temperatures, the Mössbauer spectra broaden (Figure S10) indicating at least one temperature-dependent process that is fast at 180 K relative to the Mössbauer timescale of 10^{-7} s. This can be ascribed to a slower electron hopping or to a combination with slower electronic relaxation.

Supplementary Fig. 18. Temperature-dependent zero-field Mössbauer spectra of chemically oxidized [(susan$^{6\text{-Me}}$){Fe^{III}(μ-O)(μ-O_2)Fe^{IV}}]$^{3+}$ in frozen CH_3CN solution showing a severe broadening at lower temperatures, due to a slow-down of spin relaxation, or of 'valence-hopping', or the combined effect of both.

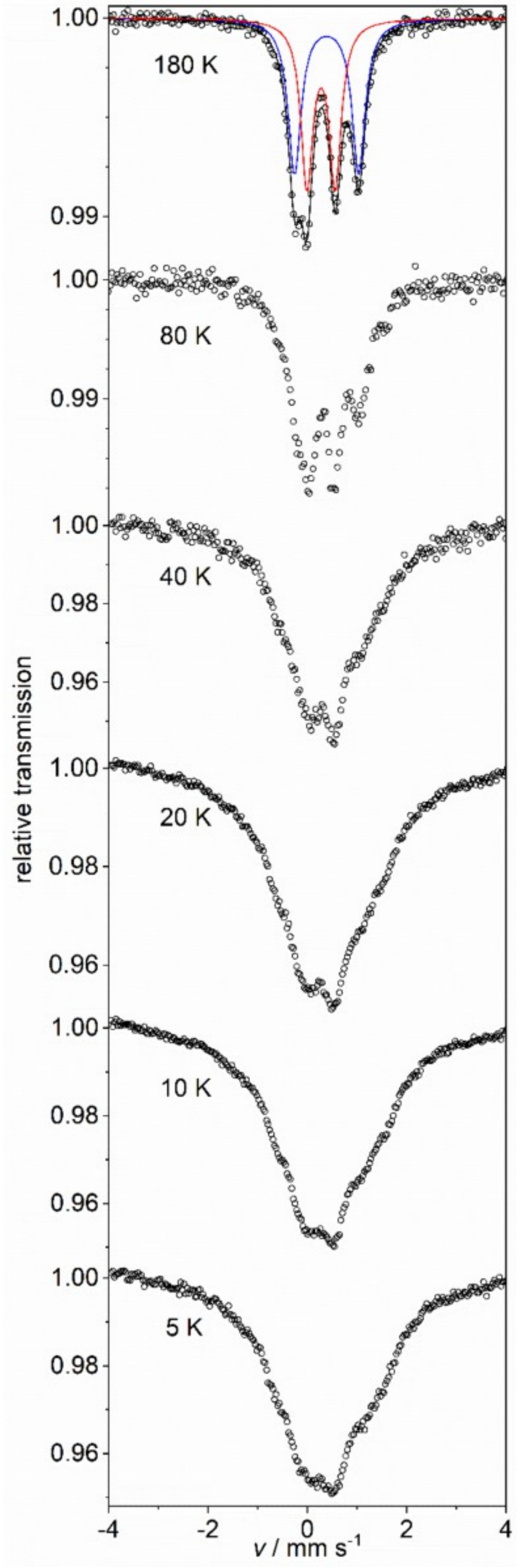

Supplementary Fig. 19. Reaction of [(susan$^{6\text{-Me}}$){Fe^{III}(μ-O)(μ-O$_2$)Fe^{III}}]$^{2+}$ with 2-phenylpropanal at -5 °C in CH_3CN. Without addition of 2-phenylpropanal, the prominent absorption features at 15400 and 19300 cm^{-1} show no significant change. Addition of 50 equivalents of 2-phenylpropanal at 360 s initiates a slow decay of these band indicating a slow but significant nucleophilic reactivity of [(susan$^{6\text{-Me}}$){Fe^{III}(μ-O)(μ-1,2-O$_2$)Fe^{III}}]$^{2+}$.

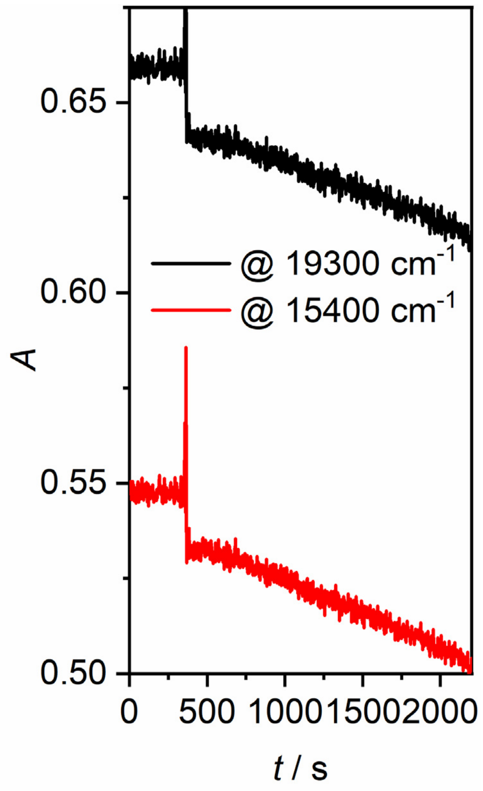

Supplementary Fig. 20. Reaction of [(susan$^{6\text{-Me}}$){Fe^{III}(μ-O)(μ-O$_2$)Fe^{III}}]$^{2+}$ (0.96 mM) with DHA (100 eq) at -40 °C in CH_3CN. Despite the change of absorption due to dilution and temperature equilibration, the spectrum of [(susan$^{6\text{-Me}}$){Fe^{III}(μ-O)(μ-O$_2$)Fe^{III}}]$^{2+}$ persists.

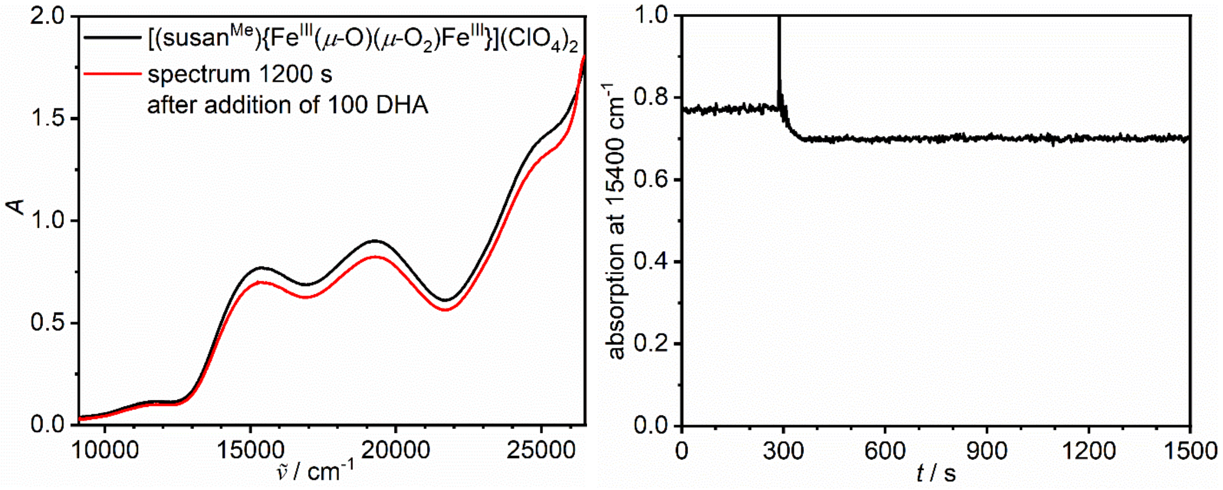

Supplementary Fig. 21. Reactivity of [(susan$^{6\text{-Me}}$){Fe^{III}(μ-O)(μ-O$_2$)Fe^{III}}]$^{2+}$ (0.95 mM) with PPh_3 (7 eq) at -40 °C in CH_3CN. Despite the change of absorption due to dilution, the spectrum of [(susan$^{6\text{-Me}}$){Fe^{III}(μ-O)(μ-O$_2$)Fe^{III}}]$^{2+}$ persists.

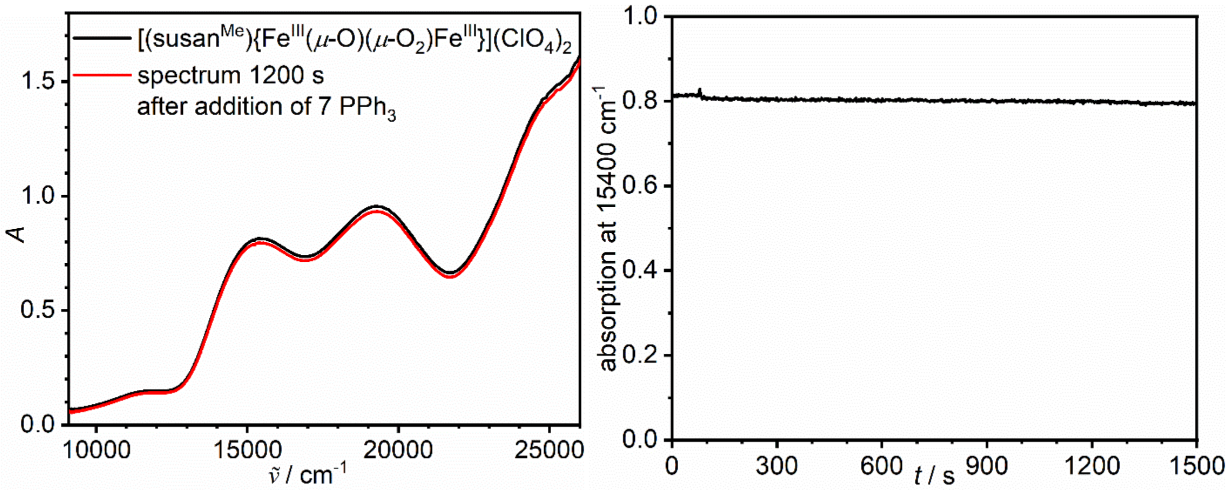

Supplementary Fig. 22. Reaction of [(susan$^{6\text{-Me}}$){Fe^{IV}(μ-O)(μ-O_2)Fe^{III}}]$^{3+}$ with DHA: a solution of [(susan$^{6\text{-Me}}$){Fe^{III}(μ-O)(μ-O_2)Fe^{III}}]$^{2+}$ (0.44 mM) with DHA (100 eq) at -60 °C in CH_3CN/CH_2Cl_2 (1:1) was reacted with a solution of (thia)(ClO_4) (1.0 eq). The spectrum of [(susan$^{6\text{-Me}}$){Fe^{IV}(μ-O)(μ-O_2)Fe^{III}}]$^{3+}$ developed quickly. Despite a slight decrease in absorption due to temperature equilibration in the first 100 s, the spectrum of [(susan$^{6\text{-Me}}$){Fe^{IV}(μ-O)(μ-O_2)Fe^{III}}]$^{3+}$ persists.

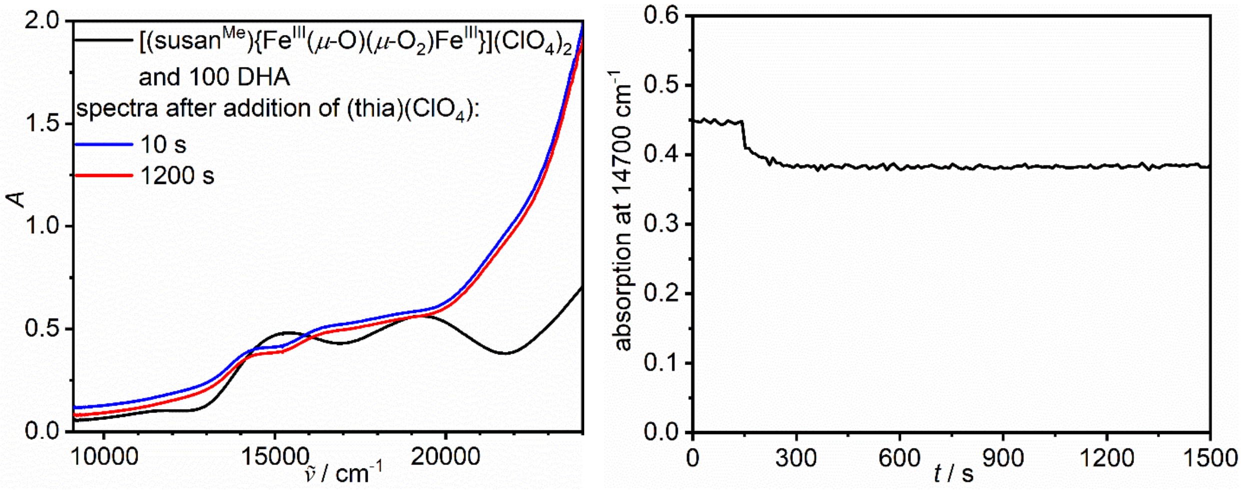

Supplementary Fig. 23. Reaction of [(susan$^{6\text{-Me}}$){Fe^{IV}(μ-O)(μ-O_2)Fe^{III}}]$^{3+}$ with PPh_3: a solution of [(susan$^{6\text{-Me}}$){Fe^{III}(μ-O)(μ-O_2)Fe^{III}}]$^{2+}$ (0.66 mM) at -60 °C in CH_3CN/CH_2Cl_2 (1:1) was reacted with a solution of (thia)(ClO_4) (1.0 eq) resulting in the spectrum of [(susan$^{6\text{-Me}}$){Fe^{IV}(μ-O)(μ-O_2)Fe^{III}}]$^{3+}$ (red line). The spike in the time trace originates from the absorption of thianthrenium ($thia^+$). Addition of a solution of 20 eq. PPh_3 resulted in restoring the spectrum of parent [(susan$^{6\text{-Me}}$){Fe^{III}(μ-O)(μ-O_2)Fe^{III}}]$^{2+}$ (blue line).

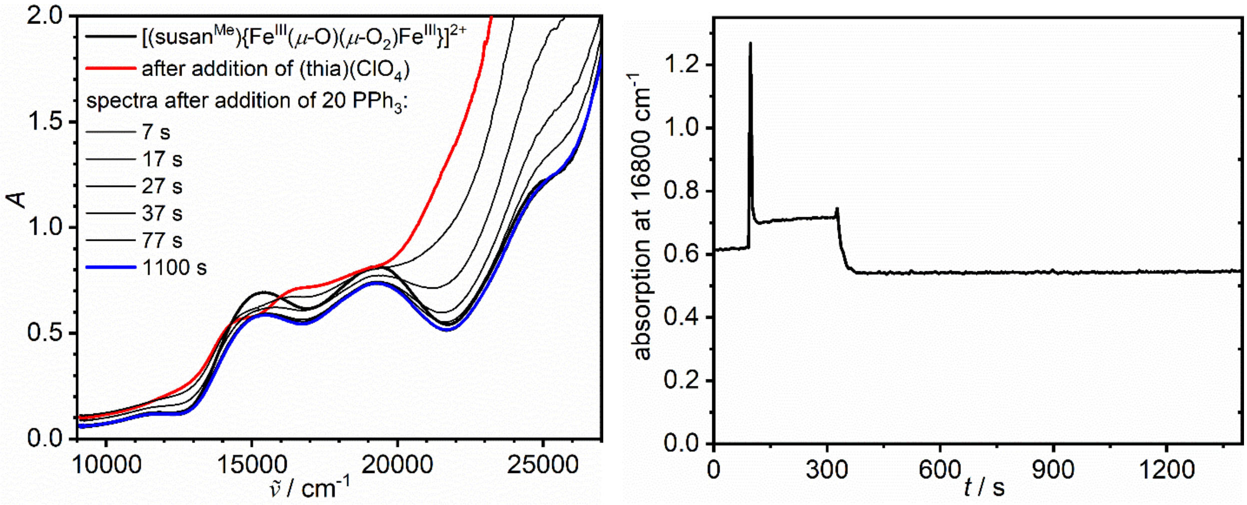

Supplementary Fig. 24. Reaction of [(susan$^{6\text{-Me}}$){Fe^{III}(μ-O)(μ-OOH)Fe^{III}}]$^{3+}$ with DHA: a solution of [(susan$^{6\text{-Me}}$){Fe^{III}(μ-O)(μ-O_2)Fe^{III}}]$^{2+}$ (0.50 mM) with DHA (100 eq) at -60 °C in CH_3CN/CH_2Cl_2 (1:2) was reacted with a solution of $HClO_4$ (1.5 eq). The spectrum of [(susan$^{6\text{-Me}}$){Fe^{III}(μ-O)(μ-OOH)Fe^{III}}]$^{3+}$ (red line) developed quickly and then showed a slow decay reaction that was also observed in a blank reaction (0.52 mM, bottom figures) without DHA demonstrating that this decay is not due to reaction with DHA.

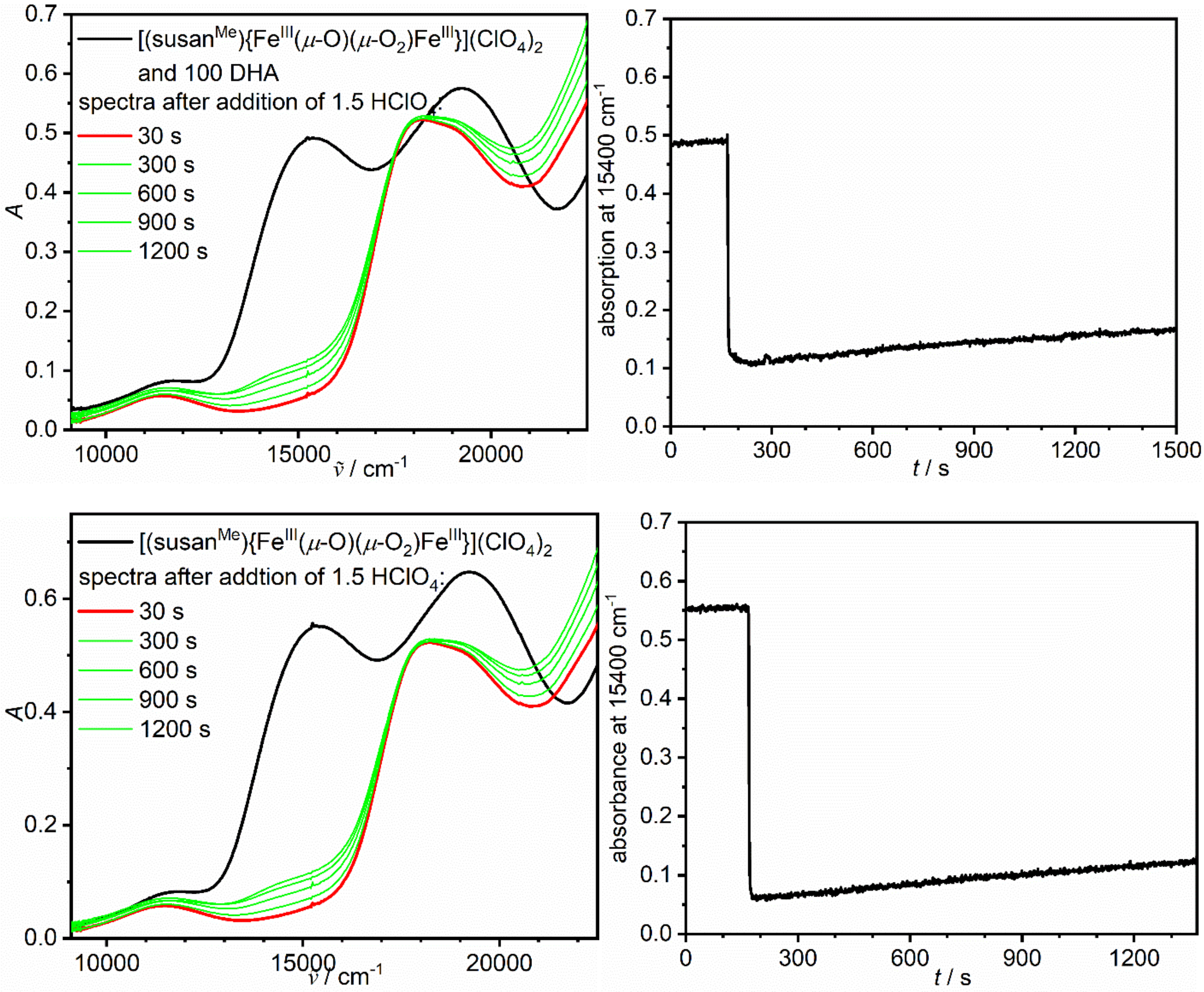

Supplementary Fig. 25. Reaction of [(susan$^{6\text{-}Me}$){Fe^{III}(μ-O)(μ-OOH)Fe^{III}}]$^{3+}$ with PPh_3: a solution of [(susan$^{6\text{-}Me}$){Fe^{III}(μ-O)(μ-O_2)Fe^{III}}]$^{2+}$ (1.1 mM) at -60 °C in CH_3CN/CH_2Cl_2 (1:2) was reacted with a solution of $HClO_4$ (1.0 eq) resulting in the spectrum of [(susan$^{6\text{-}Me}$){Fe^{III}(μ-O)(μ-OOH)Fe^{III}}]$^{3+}$ (red line). Addition of a solution of 7 eq PPh_3 resulted in the partial restoring of the spectrum of parent [(susan$^{6\text{-}Me}$){Fe^{III}(μ-O)(μ-O_2)Fe^{III}}]$^{2+}$ (blue line).

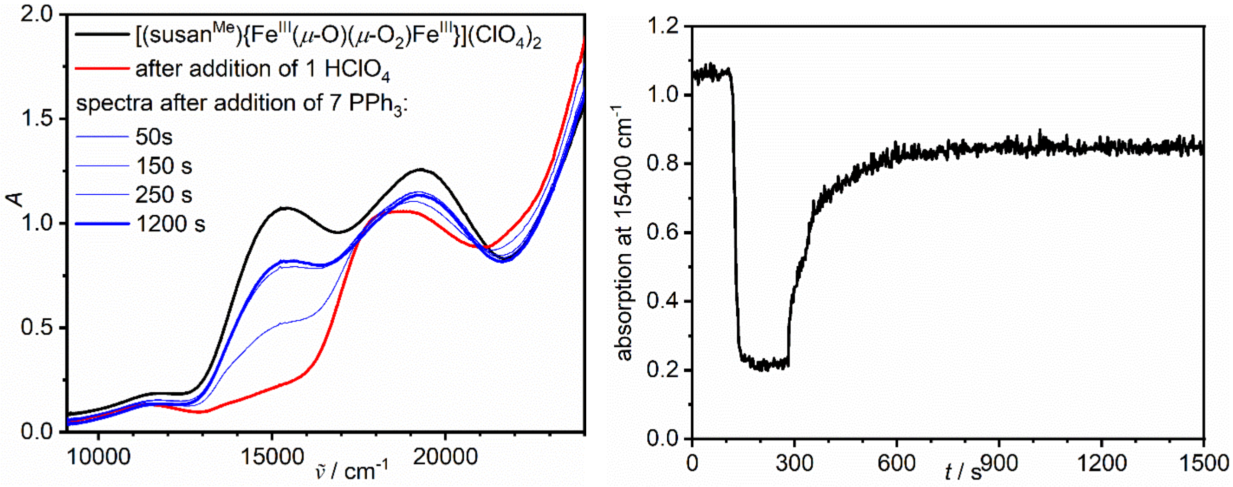

Supplementary Fig. 26. Boltzman fit of the acid-dependent formation of $[(susan^{6-Me})\{Fe^{III}(\mu\text{-}O)(\mu\text{-}OOH)Fe^{III}\}]^{3+}$ from protonation of $[(susan^{6-Me})\{Fe^{III}(\mu\text{-}O)(\mu\text{-}O_2)Fe^{III}\}]^{2+}$ at -40 °C in CH_3CN followed by the change in absorbance at 15400 cm^{-1}.

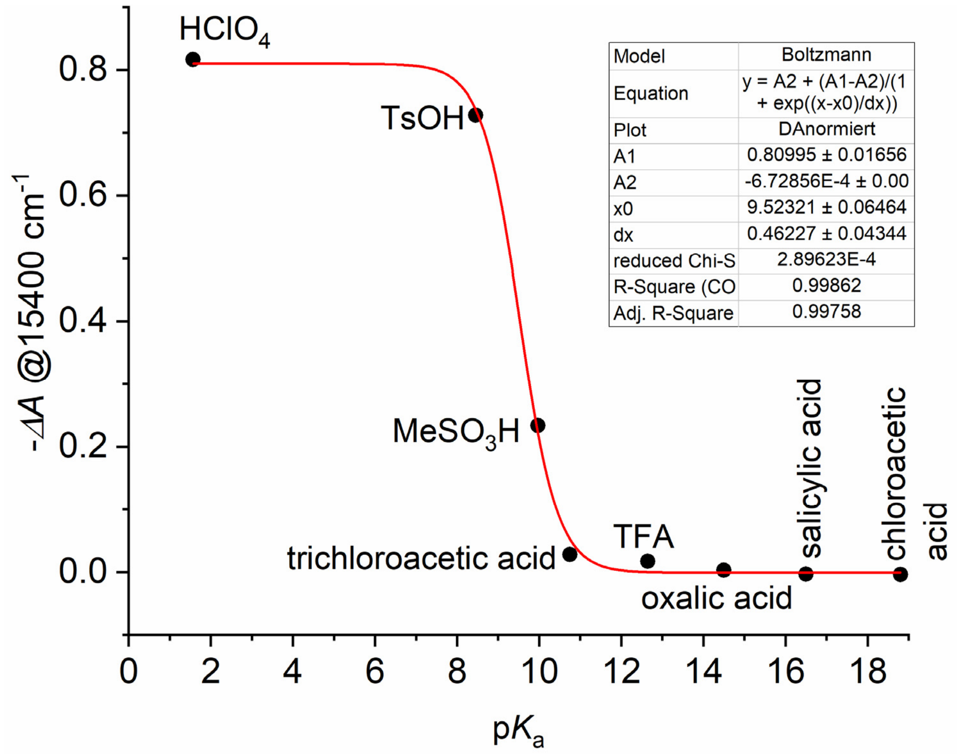

Supplementary Fig. 27. Reaction of [(susan$^{6\text{-Me}}$){Fe^{III}(μ-O)(μ-O$_2$)Fe^{III}}]$^{2+}$ (0.60 mM) with TEMPOH (100 eq) at -40 °C in CH_3CN. Despite the change of absorption due to dilution and temperature equilibration, the spectrum of [(susan$^{6\text{-Me}}$){Fe^{III}(μ-O)(μ-O$_2$)Fe^{III}}]$^{2+}$ persists.

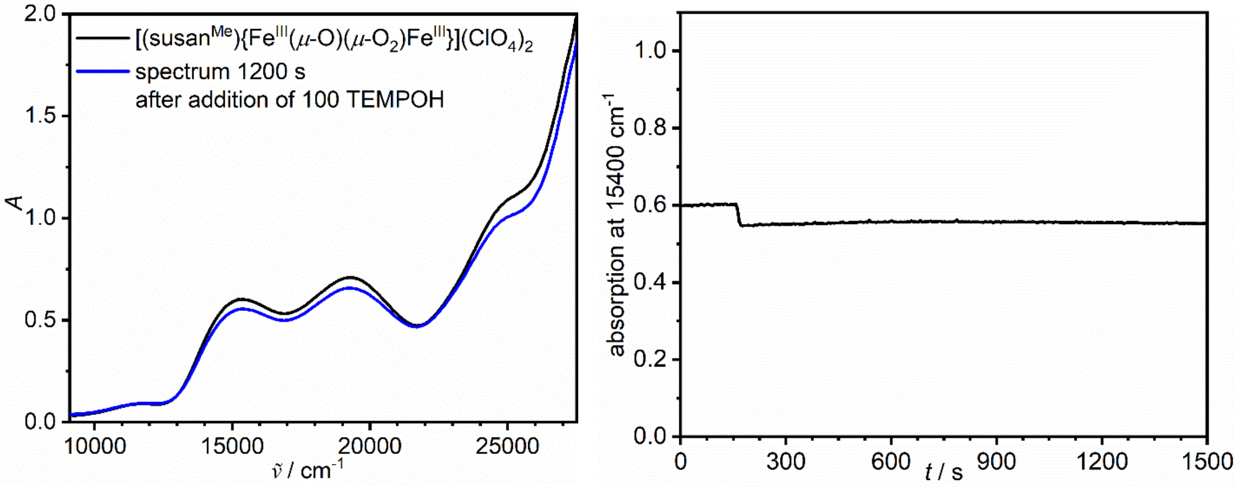

Supplementary Fig. 28. Reaction of [(susan$^{6\text{-Me}}$){Fe^{III}(μ-O)(μ-OOH)Fe^{III}}]$^{3+}$ with TEMPOH: a solution of [(susan$^{6\text{-Me}}$){Fe^{III}(μ-O)(μ-O$_2$)Fe^{III}}]$^{2+}$ (1.0 mM) at -60 °C in CH_3CN/CH_2Cl_2 (1:2) was reacted with a solution of $HClO_4$ (1.5 eq) resulting in the spectrum of [(susan$^{6\text{-Me}}$){Fe^{III}(μ-O)(μ-OOH)Fe^{III}}]$^{3+}$ (red line). Addition of a solution of 100 eq TEMPOH resulted in the restoring of the spectrum of parent [(susan$^{6\text{-Me}}$){Fe^{III}(μ-O)(μ-O$_2$)Fe^{III}}]$^{2+}$ (blue line) despite the change of absorption due to dilution and temperature equilibration.

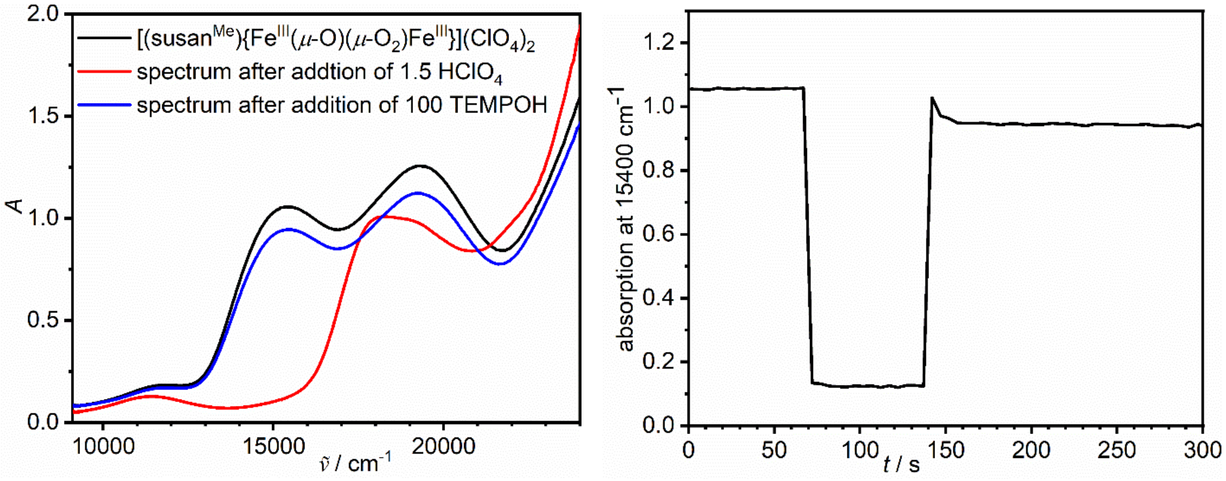

Supplementary Fig. 29. Reaction of [(susan$^{6\text{-Me}}$){Fe^{IV}(μ-O)(μ-O_2)Fe^{III}}]$^{3+}$ with TEMPOH: a solution of [(susan$^{6\text{-Me}}$){Fe^{III}(μ-O)(μ-O_2)Fe^{III}}]$^{2+}$ (0.70 mM) at -60 °C in CH_3CN/CH_2Cl_2 (1:1) was reacted with a solution of (thia)(ClO_4) (1.0 eq) resulting in the spectrum of [(susan$^{6\text{-Me}}$){Fe^{IV}(μ-O)(μ-O_2)Fe^{III}}]$^{3+}$ (red line). Addition of a solution of 100 eq. TEMPOH resulted in restoring the spectrum of parent [(susan$^{6\text{-Me}}$){Fe^{III}(μ-O)(μ-O_2)Fe^{III}}]$^{2+}$ (blue line) despite the change of absorption due to dilution and temperature equilibration.

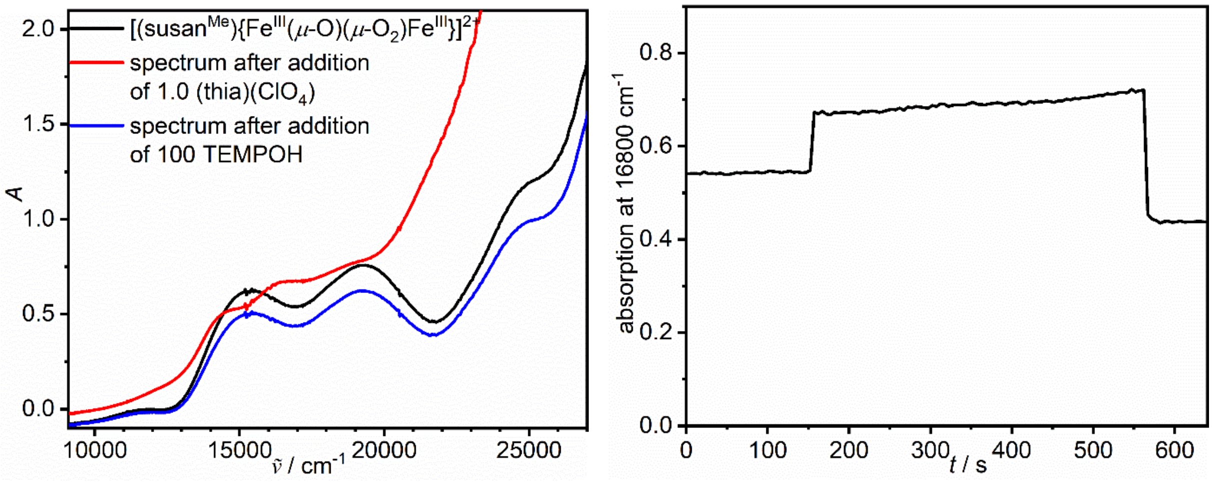

Supplementary Fig. 30. Solvent-dependence of the acidity: addition of one equivalent $HClO_4$ to a solution of $[(susan^{6\text{-}Me})\{Fe^{III}(\mu\text{-}O)(\mu\text{-}O_2)Fe^{III}\}]^{2+}$ (0.84 mM) at -40 °C in CH_3CN and addition of one equivalent $[HPPh_3]BF_4$ to a solution of $[(susan^{6\text{-}Me})\{Fe^{III}(\mu\text{-}O)(\mu\text{-}O_2)Fe^{III}\}]^{2+}$ (0.85 mM) at -40 °C in CH_3CN.

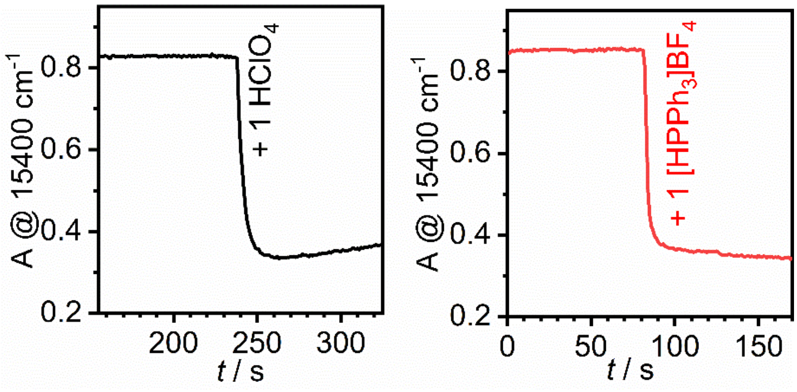

The protonated $[(susan^{6\text{-}Me})\{Fe^{III}(\mu\text{-}1,2\text{-}OOH)(\mu\text{-}O)_2Fe^{III}\}]^{3+}$ was deprotonated by addition of PPh_3 in CH_3CN:CH_2Cl_2 (1:2) at -60 °C (Supplementary Fig. 25) despite its apparently higher pK_a value (9.5 *vs* 7.62). However, these pK_a values were determined in CH_3CN. It was found that pK_a values are strongly solvent dependent.[5–8,39–43] Hence, the inverted acidity observed in CH_3CN:CH_2Cl_2 (1:2) is attributed to a different solvent dependence of the pK_a values of these two acids. The addition of either one equivalent of the acid $HClO_4$ or one equivalent of $[HPPh_3](BF_4)$ under identical conditions in CH_3CN at -40 °C lead to a direct decrease of the μ-1,2-peroxo→Fe^{III} LMCT at 15400 cm^{-1}. Hence, both acids $HClO_4$ and $[HPPh_3](BF_4)$ are able to protonate the parent complex $[(susan^{6\text{-}Me})\{Fe^{III}(\mu\text{-}1,2\text{-}O_2)(\mu\text{-}O)_2Fe^{III}\}]^{2+}$ to $[(susan^{6\text{-}Me})\{Fe^{III}(\mu\text{-}1,2\text{-}OOH)(\mu\text{-}O)_2Fe^{III}\}]^{3+}$. These experiments demonstrate the correct higher acidity of $[HPPh_3]^+$ than of $[(susan^{6\text{-}Me})\{Fe^{III}(\mu\text{-}1,2\text{-}OOH)(\mu\text{-}O)_2Fe^{III}\}]^{3+}$ in pure CH_3CN. The same applies to the reaction with TEMPOH (Supplementary Fig. 28) for which a pK_a value was only determined in water.[44]

Supplementary Table 7. Selected interatomic distances [Å] and angles [°] for [(susan$^{6\text{-Me}}$){Fe(μ-OH)$_2$Fe}](ClO_4)$_2$·MeOH.

Fe1-O3	1.9548(11)	C49-C50	1.390(2)
Fe1-O4	2.2236(11)	C50-C56	1.499(2)
Fe1-N1	2.3703(13)	C50-N43	1.352(2)
Fe1-N2	2.2114(13)	N43-C46	1.346(2)
Fe1-N3	2.2547(13)	N42-C65	1.485(2)
Fe1-N4	2.2714(13)	C65-C66	1.505(2)
Fe2-O3	2.1550(12)	C66-C67	1.394(2)
Fe2-O4	1.9611(11)	C67-C68	1.385(3)
Fe2-N41	2.4389(13)	C68-C69	1.376(3)
Fe2-N42	2.2096(13)	C69-C70	1.396(2)
Fe2-N43	2.2450(13)	C70-C76	1.495(2)
Fe2-N44	2.2585(13)	C70-N44	1.351(2)
Fe1···Fe2	3.2150(3)	N44-C66	1.352(2)
N1-C4	1.482(2)		
N1-C2	1.4882(19)	Fe1-O3-Fe2	102.83(5)
C2-C3	1.520(2)	Fe1-O4-Fe2	100.21(5)
C3-N2	1.4788(19)	O3-Fe1-O4	77.65(5)
N2-C5	1.4797(19)	O3-Fe1-N1	100.05(5)
C5-C6	1.509(2)	O3-Fe1-N2	165.07(5)
C6-C7	1.385(2)	O3-Fe1-N3	104.73(5)
C7-C8	1.383(2)	O3-Fe1-N4	117.16(5)
C8-C9	1.382(3)	O4-Fe1-N1	93.76(4)
C9-C10	1.391(2)	O4-Fe1-N2	87.45(4)
C10-C16	1.499(2)	O4-Fe1-N3	84.14(4)
C10-N3	1.350(2)	O4-Fe1-N4	165.13(5)
N3-C6	1.351(2)	N1-Fe1-N2	79.68(5)
N2-C25	1.4827(19)	N1-Fe1-N3	154.03(5)
C25-C26	1.504(2)	N1-Fe1-N4	82.76(4)
C26-C27	1.390(2)	N2-Fe1-N3	74.37(5)
C27-C28	1.384(2)	N2-Fe1-N4	77.71(5)
C28-C29	1.385(3)	N3-Fe1-N4	92.65(5)
C29-C30	1.393(2)	O3-Fe2-O4	79.21(5)
C30-C36	1.496(2)	O3-Fe2-N41	91.71(4)
C30-N4	1.355(2)	O3-Fe2-N42	91.60(5)
N4-C26	1.347(2)	O3-Fe2-N43	90.27(5)
N1-C1	1.4872(19)	O3-Fe2-N44	169.34(5)
C1-C41	1.534(2)	O4-Fe2-N41	96.91(5)
C41-N41	1.4898(19)	O4-Fe2-N42	169.48(5)
N41-C44	1.477(2)	O4-Fe2-N43	109.63(5)
N41-C42	1.483(2)	O4-Fe2-N44	109.83(5)
C42-C43	1.514(3)	N41-Fe2-N42	78.11(5)
N42-C43	1.486(2)	N41-Fe2-N43	153.27(5)
N42-C45	1.475(2)	N41-Fe2-N44	81.77(4)
C45-C46	1.513(2)	N42-Fe2-N43	75.19(5)
C46-C47	1.387(2)	N42-Fe2-N44	78.85(5)
C47-C48	1.381(3)	N43-Fe2-N44	91.83(5)
C48-C49	1.386(3)		

Supplementary Table 8. Selected interatomic distances [Å] and angles [°] for [(susan$^{6\text{-Me}}$){Fe(μ-O)(μ-1,2-O_2)Fe}]$(ClO_4)_2$·0.85CH_3CN·0.7H_2O

Fe1-O1	1.8754(11)	C49-C50	1.387(3)
Fe1-O3	1.8237(11)	C50-C56	1.498(2)
Fe1-N1	2.3443(14)	C50-N43	1.348(2)
Fe1-N2	2.1835(14)	N43-C46	1.351(2)
Fe1-N3	2.2373(14)	N42-C65	1.483(2)
Fe1-N4	2.3134(15)	C65-C66	1.501(2)
Fe2-O2	1.9283(12)	C66-C67	1.385(2)
Fe2-O3	1.7901(11)	C67-C68	1.387(3)
Fe2-N41	2.2879(14)	C68-C69	1.377(3)
Fe2-N42	2.2141(14)	C69-C70	1.396(2)
Fe2-N43	2.2096(14)	C70-C76	1.493(2)
Fe2-N44	2.2833(14)	C70-N44	1.354(2)
Fe1···Fe2	3.1729(3)	N44-C66	1.356(2)
O1-O2	1.4318(16)		
N1-C4	1.479(2)	Fe1-O3-Fe2	122.80(6)
N1-C2	1.492(2)	O1-Fe1-N1	98.05(5)
C2-C3	1.514(2)	O1-Fe1-N2	175.24(5)
C3-N2	1.485(2)	O1-Fe1-N3	107.18(5)
N2-C5	1.479(2)	O1-Fe1-N4	98.27(5)
C5-C6	1.503(2)	O1-Fe1-O3	92.03(5)
C6-C7	1.385(2)	O3-Fe1-N1	91.12(5)
C7-C8	1.383(3)	O3-Fe1-N2	92.10(5)
C8-C9	1.379(3)	O3-Fe1-N3	92.45(5)
C9-C10	1.395(2)	O3-Fe1-N4	168.83(5)
C10-C16	1.497(3)	N2-Fe1-N1	79.50(5)
C10-N3	1.351(2)	N2-Fe1-N3	75.00(5)
N3-C6	1.352(2)	N2-Fe1-N4	77.45(5)
N2-C25	1.480(2)	N3-Fe1-N1	154.36(5)
C25-C26	1.502(3)	N3-Fe1-N4	88.66(5)
C26-C27	1.386(2)	N4-Fe1-N1	83.18(5)
C27-C28	1.392(3)	O2-Fe2-O3	90.49(5)
C28-C29	1.373(3)	O2-Fe2-N41	87.35(5)
C29-C30	1.398(3)	O2-Fe2-N42	89.87(5)
C30-C36	1.492(3)	O2-Fe2-N43	85.10(5)
C30-N4	1.359(2)	O2-Fe2-N44	166.03(5)
N4-C26	1.355(2)	O3-Fe2-N41	101.87(5)
N1-C1	1.493(2)	O3-Fe2-N42	178.80(5)
C1-C41	1.527(2)	O3-Fe2-N43	106.05(5)
C41-N41	1.491(2)	O3-Fe2-N44	103.35(5)
N41-C44	1.484(2)	N41-Fe2-N42	79.29(5)
N41-C42	1.492(2)	N41-Fe2-N43	151.10(5)
C42-C43	1.513(2)	N41-Fe2-N44	87.88(5)
C43-N42	1.488(2)	N42-Fe2-N43	72.85(5)
N42-C45	1.479(2)	N42-Fe2-N44	76.33(5)
C45-C46	1.510(2)	N43-Fe2-N44	92.82(5)
C46-C47	1.382(2)	Fe1-O1-O2	116.73(8)
C47-C48	1.388(3)	Fe2-O2-O1	117.50(8)
C48-C49	1.384(3)	Fe1-O1-O2-Fe2	7.13(12)

Supplementary Table 9. Selected interatomic distances [Å] and angles [°] for [(susan$^{6\text{-Me,ox}}$){$Fe^{III}(OH)_{0.2}$(μ-O)$Fe^{III}(OH)_{0.65}$}]$(ClO_4)_2$ [$C_{36}H_{48.1}N_8Fe_2O_{3.8}$]$(ClO_4)_2$

Fe1-O3	1.751(4)	C65-C66	1.512(8)
Fe1-O51*	1.998(5)	C66-C67#	1.366(18)
Fe1-O53**	1.91(3)	C67-C68#	1.37(2)
Fe1-N1	2.309(5)	C68-C69#	1.39(3)
Fe1-N2	2.231(5)	C69-C70#	1.39(3)
Fe1-N3	2.256(5)	C70-C76#	1.50(2)
Fe1-N4	2.151(5)	C70-N44#	1.38(2)
Fe2-O3	1.810(4)	C66-C67B##	1.43(3)
Fe2-O54#	1.905(7)	C67B-C68B##	1.35(4)
Fe2-O55##	1.984(13)	C68B-C69B##	1.42(4)
Fe2-N41	2.268(5)	C69B-C70B##	1.36(5)
Fe2-N42	2.244(5)	C70B-C55##	1.49(4)
Fe2-N43	2.261(5)	C55-O55##	1.38(3)
Fe2-N44	2.242(5)	C70B-N44##	1.33(4)
Fe1 ··· Fe2	3.5264(12)	N44-C66	1.334(8)
N1-C4	1.490(8)		
N1-C2	1.483(8)	Fe1-O3-Fe2	163.9(3)
C2-C3	1.514(10)	O3-Fe1-O51*	103.3(2)
C3-N2	1.478(8)	O3-Fe1-O53**	77.7(11)
N2-C5	1.489(7)	O3-Fe1-N1	94.42(19)
C5-C6	1.508(9)	O3-Fe1-N2	105.4(2)
C6-C7	1.414(9)	O3-Fe1-N3	88.91(19)
C7-C8	1.369(11)	O3-Fe1-N4	177.4(2)
C8-C9	1.385(12)	O51-Fe1-N1*	95.08(19)
C9-C10	1.380(9)	O51-Fe1-N2*	151.0(2)
C10-C16	1.495(10)	O51-Fe1-N3*	110.3(2)
C10-N3	1.355(8)	O51-Fe1-N4*	75.4(2)
N3-C6	1.344(8)	O53-Fe1-N1**	100.0(8)
N2-C25	1.503(7)	O53-Fe1-N2**	176.8(11)
C25-C26	1.514(8)	O53-Fe1-N3**	106.9(8)
C26-C27	1.399(8)	O53-Fe1-N4**	100.9(11)
C27-C28	1.387(10)	N1-Fe1-N2	79.03(17)
C28-C29	1.377(10)	N1-Fe1-N3	152.94(18)
C29-C30	1.374(9)	N1-Fe1-N4	88.03(18)
C30-N4	1.337(8)	N2-Fe1-N3	74.21(17)
N4-C26	1.331(8)	N2-Fe1-N4	76.05(18)
C30-C36**	1.46(5)	N3-Fe1-N4	89.37(17)
C30-C51*	1.541(10)	O3-Fe2-O54#	90.7(3)
C51-O51*	1.295(11)	O3-Fe2-O55##	117.0(5)
C51-O52*	1.236(11)	O3-Fe2-N41	91.8(2)
N1-C1	1.495(8)	O3-Fe2-N42	93.1(2)
C1-C41	1.535(9)	O3-Fe2-N43	88.20(18)
C41-N41	1.490(8)	O3-Fe2-N44	169.0(2)
N41-C44	1.480(8)	O54-Fe2-N41#	99.1(3)
N41-C42	1.486(9)	O54-Fe2-N42#	176.0(3)
C42-C43	1.518(10)	O54-Fe2-N43#	107.7(3)
C43-N42	1.485(9)	O54-Fe2-N44#	100.0(3)
N42-C45	1.471(8)	O55-Fe2-N41##	100.0(5)
C45-C46	1.507(10)	O55-Fe2-N42##	149.8(5)
C46-C47	1.393(10)	O55-Fe2-N43##	103.9(5)
C47-C48	1.377(13)	O55-Fe2-N44##	73.5(5)

C48-C49	1.344(14)	N41-Fe2-N42	79.51(19)
C49-C50	1.413(12)	N41-Fe2-N43	153.2(2)
C50-C56	1.500(13)	N41-Fe2-N44	89.24(19)
C50-N43	1.337(9)	N42-Fe2-N43	73.7(2)
N43-C46	1.346(9)	N42-Fe2-N44	76.28(18)
N42-C65	1.487(8)	N43-Fe2-N44	85.97(18)

* C51, O51, O52 are part of an 80% occupied majority at Fe1.

** C36, O53 represents the corresponding minority (20%) at Fe1.

C67, C68, C69, C70, C76, O54 are part of an 65% occupied majority at Fe2.

C67B, C68B, C69B, C70B, C55, O55 represents the corresponding minority (35%) at Fe1

Supplementary Table 10. Cartesian coordinates of TPSSh/def-2TZVP optimized structures

[(susan^{6-Me}){Fe^{III}(μ-O)(μ-1,2-O_2)Fe^{III}}]$^{2+}$:

Fe	1.48141127208994	3.93947445952962	11.86044347936161
Fe	2.18802730531374	6.07942766621876	9.66293022196163
O	0.16197457769711	5.21199619710279	11.47569160587574
O	0.51456450285503	6.25995554837451	10.60729538508803
O	2.63120638286073	4.65134918588664	10.64055036058696
N	0.82929066928865	2.25451362242775	10.36678769369309
N	2.94391623822796	2.35880544312651	12.38280574663005
N	2.70290064048354	4.80772468074875	13.53481993937438
N	0.30154017024232	2.71015956668201	13.35049290175088
N	1.01967487326382	5.07785920003744	7.99540048371123
N	1.61988368760784	7.87047589152924	8.44727169061477
N	2.81036659510794	7.87339609820870	10.80031289785160
N	3.95908966582934	6.32907716532708	8.24844096641257
C	1.15219470899845	2.75392799500280	9.00265923404247
H	1.02917189961001	1.92498318891483	8.28859889749609
H	2.20272294692220	3.05136077194192	9.00817929123729
C	1.69259069296075	1.09058794850504	10.69001039196129
H	1.79344662516833	0.43034594348303	9.81612211254167
H	1.19141773161141	0.50920197529182	11.47157805690346
C	3.06679900358259	1.52250448123829	11.17033089763924
H	3.68873498926343	0.63701069387032	11.36648176553667
H	3.56951360312443	2.12755454642917	10.40934475570027
C	-0.58596607851583	1.82979162994326	10.41801352302392
H	-0.80390050511094	1.39770070410394	11.39659399354050
H	-0.79106485886491	1.07294367606114	9.64614653548149
H	-1.24364575983057	2.68667881978016	10.25741312965665
C	4.18804286110915	3.08169809659017	12.69162813114795
H	4.55654305005441	3.52130787702782	11.75728781444856
H	4.96266804901137	2.41045573959771	13.08741262541109
C	3.89803586968475	4.19395737176457	13.66680928582404
C	4.83753938527748	4.58783189505115	14.61938407306162
H	5.77911977274658	4.05188909801881	14.70599323645215
C	4.53812628727204	5.67666931966054	15.44050094841285
H	5.24698362623140	6.01388167205550	16.19339257377336
C	3.31587429413695	6.32658869389613	15.27713201251352
H	3.05387153623770	7.18337233683953	15.89208106104966
C	2.40377000859607	5.87007797896514	14.31356405710597
C	1.07986284826341	6.54793396062200	14.12320694612012
H	0.94905050997900	6.85267676751834	13.07991345088774
H	1.00010016368863	7.42228487917121	14.77372761424084
H	0.26710960135967	5.85478061653603	14.36600415334960
C	2.48573557466823	1.60454101946377	13.56464415485335
H	2.94031936934309	2.06107355576464	14.45351432753409
H	2.84353619710292	0.56719659074114	13.52778675288572
C	0.99299338008023	1.63161242798038	13.77907577195925
C	0.39436851072823	0.58218626832714	14.48135487668998
H	0.99266571763425	-0.27380786446469	14.78284119258319

C	-0.96253601616166	0.66488886015685	14.78553000600641
H	-1.46069741313994	-0.13364503432638	15.33076050614924
C	-1.66725551380588	1.79651976086654	14.37817126557736
H	-2.72422827959444	1.90524782977028	14.60592243306681
C	-1.02053371151933	2.80540246790959	13.65174438561946
C	-1.80189690344641	4.00113535540053	13.19367761509671
H	-1.81501383410699	4.06642744222257	12.10144667932352
H	-1.34579581092309	4.92735825003594	13.55383697691987
H	-2.82806771602778	3.93958317666817	13.56430655193614
C	0.27259938539767	3.94403863368240	8.60036715415621
H	-0.50208409160925	3.61690406265845	7.89038735558795
H	-0.22497457883555	4.34670481090119	9.48618299570672
C	0.03063893884680	6.13456824186188	7.65350588977699
H	-0.62735760872742	6.24567526565201	8.52058319972204
H	-0.57806526943242	5.82297951340862	6.79121380974705
C	0.71105808142909	7.45883293422055	7.34659157383605
H	1.31485039594030	7.37317420639142	6.43744908863622
H	-0.04442353595758	8.23152542811864	7.15524439801298
C	1.75504129600164	4.63500304144860	6.79297305430485
H	2.25688468977366	5.48160663731314	6.32020618564019
H	1.06404727713378	4.18082906418039	6.06745733413708
H	2.51518960210309	3.90052258802961	7.06850353252146
C	0.99815032419280	8.76368954968240	9.44522278056318
H	0.02775098382754	8.34245544521356	9.72946602175402
H	0.82899276109122	9.76825419231158	9.03054474772904
C	1.89042394082966	8.85081643218650	10.66725137079401
C	1.79095419884865	9.89037223118937	11.59133051192416
H	1.04183638378341	10.66739190906977	11.46139784680610
C	2.67460685424336	9.90355407953129	12.67481410061228
H	2.61185020786130	10.68988591831833	13.42398477623248
C	3.65707736625411	8.91709735331863	12.76949510097808
H	4.38009524182454	8.92394110920776	13.58110009654538
C	3.71539455405089	7.90756374024769	11.79873691452681
C	4.79305546559806	6.86544273422756	11.78504926413253
H	4.36545591997795	5.89138144616640	11.52745950696344
H	5.30065142233849	6.80718923551570	12.75077872231206
H	5.53531157207056	7.11991501274241	11.01720462307179
C	2.85483715887703	8.47207162963874	7.90441251728365
H	3.33846881123164	9.05173230422529	8.69850711953203
H	2.62549359454583	9.16587323166230	7.08582007284512
C	3.82785849754477	7.41543054625161	7.45208513267649
C	4.58670897152023	7.59306943625160	6.29563780146606
H	4.43128092264881	8.47335419602972	5.67736276519996
C	5.53019845397933	6.62066012846412	5.95466656212729
H	6.12653220795919	6.71929585889157	5.05037539592210
C	5.69438123566256	5.52579609428907	6.79850193795269
H	6.42813940933459	4.75481701360216	6.57664556195193
C	4.90110532731755	5.40379543551090	7.95090055616527
C	5.12402135798683	4.25801464676043	8.89231809087277
H	5.51280529020098	3.38612568346424	8.35843332606995
H	4.20310399990719	4.00805032980151	9.42468953422491
H	5.86722998815879	4.55060513876147	9.64703423129899

[(susan$^{6\text{-Me}}$){Fe^{III}(μ-O)(μ-1,2-O_2)Fe^{IV}}]$^{3+}$ (*Fe^{III}1 Fe^{IV}2 configuration*):

Fe	1.56201437903845	3.92327941252568	11.81980009130228
Fe	2.19169997478331	6.04009791005903	9.57961345266707
O	0.27392273199231	5.22244865179890	11.39545662037131
O	0.64072271558197	6.14317812908091	10.44472981330491
O	2.66041008667840	4.68133708527374	10.52052306139949
N	0.81244543764072	2.26723670066663	10.37332900454762
N	2.97279556360771	2.38303651611601	12.28951595497141
N	2.80774461238419	4.79941109971198	13.43610232560529
N	0.38097176858098	2.85976756603494	13.30287738041132
N	0.95174477574707	4.99332057783068	7.91108798459287
N	1.58506814637236	7.75491340141890	8.50953510341059
N	2.76830839632158	7.86802206238600	10.82273296467977
N	3.84946929773578	6.23962675091652	8.30045375491816
C	1.07951862083954	2.69632308989991	8.96678423895940
H	0.92359670026332	1.82899508094009	8.31182454816177
H	2.13203854994162	2.97637835353612	8.91020577545058
C	1.65751159602600	1.07806010550932	10.68925394043495
H	1.70264483406097	0.40357162039608	9.82463090738064
H	1.17132008368358	0.52800164125709	11.50071043835855
C	3.05293574490921	1.49469818825818	11.10212503931147
H	3.66730507818534	0.61317075788993	11.32353614629669
H	3.54740436299294	2.05984819523260	10.30782391632829
C	-0.61358023127533	1.87139773338611	10.49987689597907
H	-0.79203508287732	1.46679747038999	11.49668513040486
H	-0.85472547240352	1.09910558030092	9.75750484856629
H	-1.26228140464422	2.73413018147955	10.34191871249406
C	4.25428121677761	3.06985791877354	12.55688213493050
H	4.61878823701712	3.47865853299261	11.60927813239499
H	5.00843067667742	2.37105318351805	12.93767031724746
C	4.00838379619012	4.18620864009540	13.53318061232985
C	4.96835465638774	4.56850313898703	14.46505139014216
H	5.91386160044425	4.03589620761540	14.51725247481943
C	4.68072984143880	5.63681416490960	15.31709997668572
H	5.40615764996300	5.96557814662031	16.05722902081739
C	3.44507191023896	6.27056279450839	15.20641941229090
H	3.18559857297898	7.10239215284389	15.85519230773226
C	2.51157694139864	5.83124963006927	14.25721548080553
C	1.16509517347947	6.47854009492727	14.15300462423246
H	0.93899394996043	6.76944643827674	13.12379517127690
H	1.11715376989328	7.36243513107321	14.79267770968273
H	0.39206504421982	5.77624441756481	14.48524472274993
C	2.52301763000137	1.67078954345602	13.51234038307344
H	3.03295166441897	2.12126765569332	14.37234741169250
H	2.83388251501894	0.62076870700056	13.47506900015827
C	1.04536322123657	1.77416240183885	13.76876760794912
C	0.43014802318160	0.79268106543857	14.54372710915167
H	1.00514571197662	-0.06889456188539	14.87171930728334
C	-0.90988738512425	0.94895866276202	14.88939298019565
H	-1.41925659950045	0.20364736586604	15.49532864395281
C	-1.58186490554296	2.08378329108981	14.44145058644276

H	-2.62481331876666	2.24851474332307	14.69652928165854
C	-0.92941496609556	3.02471653532722	13.63645519138578
C	-1.70236275816097	4.20214373657286	13.12375719795898
H	-1.17841283906335	5.14267448691664	13.30287444484557
H	-2.67698577384010	4.23847411853197	13.61557144518756
H	-1.86346422034552	4.12310594313998	12.04350344229905
C	0.19676788990773	3.86619657957357	8.52080986841755
H	-0.55247134778427	3.51791032625052	7.79684052189460
H	-0.34152359449571	4.28095786415872	9.37452462582281
C	-0.01458050249004	6.07896288214868	7.58990989389790
H	-0.70067230204743	6.17318851182622	8.43549309225540
H	-0.60310906462972	5.81648613683204	6.70015496148484
C	0.71309824238281	7.38453995283740	7.34592399049504
H	1.36327930166625	7.30213500012245	6.47113530806261
H	0.00056491236182	8.19287265151902	7.15086007517205
C	1.67892574448710	4.55300610379141	6.70011497919404
H	2.19583866841593	5.39755075915929	6.24169905535764
H	0.97631424753973	4.12548670501166	5.97204063728741
H	2.42202117056548	3.79812413820956	6.96395109200107
C	0.90204686344267	8.63965759891287	9.49159607087324
H	-0.06955532976689	8.20249495870974	9.74083461459469
H	0.72713824382071	9.62480961100191	9.04126831360719
C	1.76847428834970	8.76441185614548	10.72579337696379
C	1.57884373989312	9.75263967882908	11.68829620784259
H	0.76219415506348	10.46329195155790	11.59238099294477
C	2.46707184816058	9.80016621452048	12.76768924045282
H	2.33951981269108	10.54725530709047	13.54760067738505
C	3.53430990680483	8.90208305937947	12.82111921712242
H	4.26093597309021	8.94556671223446	13.62805509022742
C	3.67273881017250	7.93624082816710	11.81568463839697
C	4.83508277359464	6.99230993508495	11.75034097510413
H	4.49901090152336	5.99364266516653	11.45772023788751
H	5.35704299939013	6.93737570376676	12.70800764985503
H	5.54680680190282	7.34145197047945	10.99117632570973
C	2.81761216440585	8.42369642685006	8.00775192612230
H	3.24270556671424	9.03355482264280	8.80980687717433
H	2.56616571714993	9.09443856880478	7.17991982842250
C	3.83311828404858	7.40547812079743	7.60061902153053
C	4.75033408076149	7.68789199145640	6.59291207801229
H	4.67967264813708	8.62956063661422	6.05635034563214
C	5.73834800331488	6.75063134611112	6.29741380394381
H	6.46652603791081	6.93821788657990	5.51226748756647
C	5.76883798934907	5.56792494926212	7.03092460495674
H	6.52449799651119	4.81163597059405	6.83855099295572
C	4.81768538904684	5.32357357397931	8.02844932066736
C	4.89437369311827	4.04900153274947	8.80578286335172
H	5.69190127667736	3.41828431692090	8.40889788753426
H	3.95371702367345	3.49679074731562	8.75609633701478
H	5.10415867254107	4.26205529669058	9.85782932312028

[(susan$^{6\text{-Me}}$){Fe^{IV}(μ-O)(μ-1,2-O$_2$)Fe^{III}}]$^{3+}$ (*Fe^{IV}1 Fe^{III}2 configuration*):

Fe	2.23257530550517	5.99095585917128	9.59263359729660

Fe	1.54371037403582	3.91988579636205	11.85905259897588
O	0.34960527282299	5.15859304486861	11.51049739895678
O	0.54781882927501	6.11961252061176	10.56867888589303
O	2.60901636805543	4.56064066440302	10.64453028352371
N	0.80823559323117	2.19951194384635	10.36680124210419
N	2.93626542398399	2.48421510975399	12.32627511697924
N	2.75497746268750	4.85767102802609	13.53469781110058
N	0.35011840458334	2.88301724233939	13.24165137453966
N	0.97098078956578	5.00059698909202	7.99606107194601
N	1.57408895364412	7.76297744671865	8.51094887049823
N	2.79892480283667	7.74483014803401	10.79963756906288
N	3.88367716420774	6.27043273592859	8.22569733741011
C	1.08823216709738	2.66528203446081	8.98181860284432
H	0.93497176864892	1.82376895121277	8.29211305921251
H	2.14261200672236	2.94318989718565	8.94185471786076
C	1.71565400443887	1.07438858102491	10.71477957004932
H	1.82678904253020	0.38905297603029	9.86368057149189
H	1.24923103644702	0.50525452836558	11.52490352457608
C	3.07486429285529	1.58349620480187	11.14432729542538
H	3.73942581324609	0.74885525166571	11.39644287921045
H	3.54837055218206	2.17136263464363	10.35455624797245
C	-0.59333642555653	1.73280643902400	10.46267160353217
H	-0.78194658488535	1.34637360840301	11.46567667496176
H	-0.77617786949606	0.92961758068102	9.73557556715522
H	-1.28120209195607	2.55595153345416	10.26143805297755
C	4.21978804529129	3.17314343953855	12.61867909855520
H	4.59221708363763	3.58953776821635	11.67759525496563
H	4.95807175673593	2.45571092185300	12.99427853912599
C	3.96753136258859	4.27350276194578	13.61167589185268
C	4.93024921675885	4.67485676261252	14.53425448832404
H	5.89055785142017	4.16832979752970	14.57542044228609
C	4.62296345666888	5.73394939607702	15.39181415421099
H	5.34879070941943	6.07946238911636	16.12397779789051
C	3.36955822877383	6.33739744268498	15.30014933344737
H	3.09834703288649	7.16065144363221	15.95525975039832
C	2.43747433941128	5.87611121390835	14.35991284627591
C	1.07430419986287	6.48804461480912	14.24835616154828
H	0.90910657247464	6.89671895482200	13.24646763504419
H	0.95279479674181	7.29010950927156	14.97988408951358
H	0.30607894960613	5.72998104285248	14.43350729801589
C	2.49396086780314	1.75258248043222	13.54849908146793
H	2.97887580502203	2.21854298406675	14.41422089595326
H	2.84165339123852	0.71510075919675	13.50771307553494
C	1.01572081930348	1.82224141940506	13.76717382349605
C	0.39637679666880	0.85605253967112	14.55550802149326
H	0.97752442897828	0.01800952736550	14.92953917788986
C	-0.95620117314019	1.00008820271801	14.85325804292217
H	-1.47313868006194	0.26835673057391	15.46896973808935
C	-1.63125426509744	2.10515899978147	14.34169477980227
H	-2.68525631252298	2.25757285073079	14.55499281800050
C	-0.97626624531715	3.03450424913411	13.52435349778481
C	-1.77436398271633	4.17406711631346	12.96300340245376
H	-1.35029871749519	5.14296484582591	13.23717456700407
H	-2.79307286361895	4.11477788655283	13.35230032350940

H	-1.81635282788639	4.13243453978551	11.87074170429939
C	0.21319505987167	3.85619215775859	8.58136027145693
H	-0.54511800603548	3.54199385046601	7.85201801316365
H	-0.31298541905838	4.24882808943956	9.45311388216499
C	-0.02270694756590	6.07010905962621	7.67747416964662
H	-0.67987183614544	6.17118035436431	8.54525722406616
H	-0.63332113703676	5.76855760339786	6.81586055096243
C	0.67173063447270	7.38342779453462	7.38498001291241
H	1.28881676032746	7.30173867908581	6.48591503899815
H	-0.06467507180107	8.17439988720356	7.20379273979603
C	1.67805757843509	4.57033503113674	6.76448153119645
H	2.18138117821123	5.42005043771863	6.30202396724093
H	0.95936171149841	4.14662000904721	6.05088249448220
H	2.42788184468419	3.81513868165808	7.00708851117862
C	0.92678622150356	8.62096467924832	9.53367757512641
H	-0.04264248336156	8.18783054759273	9.79679017253259
H	0.75369826029732	9.62947483693470	9.13562173575317
C	1.83137220602279	8.68471192412604	10.74353940586833
C	1.72022763690892	9.67245374403411	11.71790597217518
H	0.93065778220039	10.41640718229324	11.65372225726593
C	2.65148407545577	9.68416781320488	12.76103894130535
H	2.58328165643907	10.43207507299898	13.54757008528600
C	3.69100738668922	8.75319934531846	12.76232799767069
H	4.45738517226427	8.77173438003506	13.53224835884264
C	3.75266273392177	7.78736845815435	11.75048289004542
C	4.89116697037732	6.82208378360008	11.62657341813401
H	4.52397221702943	5.82881341129742	11.35371058959797
H	5.46009619867300	6.75599260905466	12.55617765143838
H	5.56877388141665	7.15952859733577	10.83173231753652
C	2.78968795287301	8.43544838952935	7.98473963555371
H	3.22861787230781	9.04515869675347	8.78074493069325
H	2.52175872906456	9.11283955434752	7.16654001420669
C	3.82052294109164	7.43753322401916	7.54216345696778
C	4.70955544398048	7.74487919549805	6.51448469174928
H	4.61104558858070	8.68721384105997	5.98299017882296
C	5.70909539786482	6.82734959680397	6.19162660509990
H	6.41531152885154	7.03637597137786	5.39174127291160
C	5.78216689490341	5.63573358723967	6.91134235425197
H	6.54638443384613	4.89578526349049	6.69055423508293
C	4.85553360988335	5.37215251589406	7.92553023264736
C	4.93559343981906	4.09731510644924	8.70685072802917
H	5.67796405719725	3.42804036447774	8.26769263928849
H	3.97087821055685	3.58398156251381	8.72413723773049
H	5.22431453333905	4.30245176934626	9.74337675440619

[(susan$^{6\text{-Me}}$){Fe^{III}(μ-O)(μ-1,2-HOO)Fe^{III}}]$^{3+}$ (*μ-peroxo-O1 protonated*):

Fe	1.51327930175504	3.88639814499874	11.89281267284453
Fe	2.18103252432191	6.09593651201505	9.61634201538392
O	0.09126705350549	5.27534949872677	11.41168478396838
O	0.43022244001071	6.34674617244605	10.50078019957589
O	2.54449229360997	4.68501743603601	10.65751997773233
N	0.81501102986751	2.29257801936375	10.38348142235625

N	2.94385106919306	2.36434713158791	12.35840184720488
N	2.71870791760086	4.77935350420219	13.51531278821629
N	0.31986803731679	2.70162649035859	13.36069045233554
N	1.03418370877567	5.08247501594470	7.97229108721870
N	1.62669749790293	7.85745741491105	8.41361935030742
N	2.77640143527436	7.85987974233441	10.75795575852990
N	3.93433109447976	6.30513815159948	8.24913567077597
C	1.13670500847017	2.76480019657243	9.00072425578885
H	1.00178626082665	1.91778816618573	8.31500141969576
H	2.19096441544184	3.04395394405252	8.99099764059793
C	1.65631902950263	1.09891526448990	10.69817097967399
H	1.72155190013676	0.44129856756491	9.82179015874523
H	1.15137486291277	0.53431854596919	11.48780224249365
C	3.04338103341164	1.50587166281871	11.14967972791961
H	3.65195599837917	0.61770077415381	11.36093928858900
H	3.55326423553124	2.08916514135497	10.37820529488767
C	-0.61089844634489	1.88006965031859	10.43555662360408
H	-0.83203669845072	1.45646052719780	11.41588747360664
H	-0.81021627200421	1.11939144971877	9.66922086193302
H	-1.26048129930887	2.73902146344815	10.25885378105135
C	4.20715532221628	3.07908714325919	12.64188442927733
H	4.56970417494073	3.50554584293538	11.70046136961414
H	4.97305307258116	2.39524953813973	13.02684872271871
C	3.92870318407076	4.18694557358801	13.62021185342537
C	4.87877159472103	4.59778869521332	14.55091465677196
H	5.83153308813802	4.07967978469611	14.61557404778420
C	4.57547680749990	5.67844001547294	15.38123871101939
H	5.29378038060758	6.02826531747667	16.11865089547188
C	3.33607882588644	6.30198663411874	15.24857365681082
H	3.06766740416938	7.14913527099242	15.87340188714628
C	2.41289051007082	5.83090050422302	14.30625084972360
C	1.07302586434145	6.48168588451010	14.14570980664340
H	0.96531266536268	6.90850041794349	13.14274351322156
H	0.94534377354990	7.28188809840935	14.87784187970180
H	0.27473949099472	5.74841188039491	14.29969251197620
C	2.51376594626092	1.61639001610072	13.56452263783559
H	2.98335031767112	2.08719877863877	14.43670896263255
H	2.88244818803111	0.58521051162772	13.52263579845541
C	1.02865849551478	1.63534090257587	13.79858604939819
C	0.45226286184409	0.59343595350990	14.52594313475351
H	1.06612138444596	-0.24916582977753	14.83221035379762
C	-0.90004820741027	0.66362892591751	14.84962385462010
H	-1.38004657147344	-0.13041315751852	15.41636512492270
C	-1.62385121438344	1.77813142307695	14.42966825661909
H	-2.67902813882455	1.87895394552711	14.66703350128993
C	-1.00010005863307	2.77835716526900	13.67764372928261
C	-1.81253851232310	3.94475771307603	13.19996124996376
H	-1.37372078940226	4.89210763420433	13.53102220204393
H	-2.82234526596737	3.89237989946201	13.61239153126268
H	-1.90481576717372	3.93849651372656	12.10786504401677
C	0.26638934350851	3.94758002857127	8.56134892193631
H	-0.47408261375872	3.61322730664885	7.82230298068380
H	-0.27923237418247	4.35862979420759	9.41117373683267
C	0.04675858785848	6.13599616085308	7.58480311961965

H	-0.64220017706351	6.25391287688456	8.42474679182369
H	-0.52593114225552	5.80700224464414	6.70689886604167
C	0.74080478879050	7.45155684103379	7.28493266992019
H	1.36931369073664	7.35932191598222	6.39463656181936
H	-0.00079070611704	8.23123128336799	7.07643353475493
C	1.78829022135968	4.62156273770527	6.78069500257974
H	2.29141705479758	5.46302308143593	6.30301399962181
H	1.10019019462116	4.15942095762751	6.06038738663702
H	2.54314746042069	3.88993717395220	7.07458577323613
C	0.98881675370234	8.77102108210502	9.39044928331648
H	0.00082120957726	8.37717127939167	9.64852531656058
H	0.85519018408152	9.77304522607057	8.96058648856505
C	1.86409331951177	8.84579008947629	10.62284833617682
C	1.77441676841307	9.88415842898397	11.54601534897819
H	1.03294854686165	10.66785846978231	11.41489001729811
C	2.66092572109980	9.89193210517311	12.62730678034492
H	2.60543110377465	10.67884852187019	13.37597349143506
C	3.64144053178905	8.90302033358849	12.71608060945144
H	4.37292554417205	8.91096579116237	13.51971304449295
C	3.69135772361505	7.89316114736769	11.74743504558351
C	4.77897448760801	6.86332016323909	11.71685895643173
H	4.37043035364373	5.88072758365849	11.46434018309678
H	5.30027357066427	6.81082004386651	12.67499995799714
H	5.50953945969677	7.13162150670662	10.94279357964573
C	2.87314698998015	8.46747922347559	7.89267290840300
H	3.33143184295725	9.06213721863386	8.68939106926766
H	2.65007971287394	9.14747799175405	7.06272632188243
C	3.85946376245538	7.41456170587052	7.47498456480432
C	4.68832924497893	7.61591484224371	6.37432454892316
H	4.57609759852350	8.51430046444045	5.77353758748801
C	5.64443113568346	6.64675431104962	6.06421508553635
H	6.29731701324884	6.76700544774269	5.20292857796445
C	5.74184871165342	5.52339518705540	6.87992014085818
H	6.47607109996660	4.74793694073869	6.67766336077998
C	4.87788630111215	5.37325134279544	7.97381258982460
C	5.01975793320151	4.18113337389093	8.87009685565457
H	5.39655845684812	3.32043666447690	8.31100846593299
H	4.06992352573192	3.93341238955449	9.34597366197153
H	5.74054053459883	4.40529389494427	9.66785700977391
H	-0.74964773018836	5.57687524081298	11.79550346880750

[(susan$^{6\text{-Me}}$){Fe^{III}(μ-O)(μ-1,2-OOH)Fe^{III}}]$^{3+}$ (*μ-peroxo-O2 protonated*):

Fe	1.48319342952942	3.95653389475063	11.87685131587710
Fe	2.31381162595293	6.08032750861384	9.51765718199119
O	0.03514041980601	5.15087605608076	11.58543114747739
O	0.41429410692649	6.27105171866177	10.75692801803538
O	2.56721323918629	4.71254478011366	10.61227072695204
N	0.79581691861608	2.30025572856580	10.41300088929924
N	2.93359485899269	2.40741538628998	12.35791699964406
N	2.70702916320392	4.83414610760842	13.50629842043008
N	0.31876934432166	2.72052029846733	13.40203961493813
N	1.01380848619951	5.08669338102156	8.00284586223446

N	1.66424527096556	7.83864723521759	8.36631844699066
N	2.78135207883283	7.81660064513564	10.72064555797735
N	3.97248964641712	6.29130484611720	8.17655618482237
C	1.09196021749404	2.77203435284740	9.02971855125353
H	0.92661356863110	1.93663541683857	8.33582970323905
H	2.15084098126029	3.03157774886613	8.99928023835640
C	1.64993296429392	1.11637888086173	10.71714402057920
H	1.71598471228529	0.45855679229319	9.84042338809002
H	1.15987493199136	0.54601309675574	11.51177380497366
C	3.03563122676156	1.54337500393351	11.15661171782953
H	3.65793664610211	0.66329225763234	11.36429328589004
H	3.53059789590610	2.12932534043783	10.37715494104707
C	-0.62850747190327	1.89182528429943	10.49237810592557
H	-0.82945763110600	1.46363573130956	11.47495007210497
H	-0.84841500375685	1.13677916626286	9.72557787193489
H	-1.27601902024690	2.75692749887439	10.33943062331583
C	4.19194915779733	3.13202868803586	12.62973064667722
H	4.53933283541571	3.56320324083648	11.68460698351516
H	4.97142964525223	2.45807764064156	13.00621981305001
C	3.91307742047795	4.23490321772088	13.61469944634246
C	4.85675063412277	4.62643979571063	14.56054207714771
H	5.80739108764824	4.10416878150338	14.62462360499550
C	4.54809813814807	5.69167713359191	15.40897155996617
H	5.26028238643755	6.02630243889943	16.15940107339233
C	3.30966998030135	6.31711346227590	15.27925952096608
H	3.03576481494851	7.14993192236220	15.92097287389139
C	2.39375202659955	5.86791876394550	14.31789272382613
C	1.05323504808192	6.52050179473447	14.17041372437439
H	0.91428434717710	6.90613540162880	13.15590276415567
H	0.95078297499382	7.34343515991142	14.88143832102908
H	0.25722033761054	5.79373813307889	14.36041804496080
C	2.52229209608718	1.65826364608069	13.56755143938743
H	2.98245441963603	2.14479601109131	14.43627896291145
H	2.91225514503270	0.63420318596064	13.53396689378891
C	1.03754512758773	1.64938168178038	13.80602130142431
C	0.47745310282155	0.57975037766605	14.50588794821266
H	1.10115377019949	-0.26581286785023	14.78344827093245
C	-0.87375377648500	0.62915869646668	14.84017200294417
H	-1.34449891977692	-0.18603591626842	15.38462951934929
C	-1.60650649026982	1.75226595067637	14.46311542674273
H	-2.65975017312670	1.83889501753928	14.71550015661022
C	-0.99830286553561	2.78355223413400	13.73580554159923
C	-1.82825420964258	3.95822361465921	13.31173857095629
H	-1.36626665481034	4.90199517531191	13.61043340435663
H	-2.82007214035166	3.88821214031853	13.76473976763183
H	-1.94166848624982	3.98914889109107	12.22354369225043
C	0.23569565223843	3.97688500383004	8.63024160534982
H	-0.54547229375166	3.66831007728029	7.92310781895331
H	-0.25505317494412	4.39477139005570	9.51104219683889
C	0.04597321764090	6.14878676802445	7.59650918553695
H	-0.64115313265877	6.30815548856718	8.43219752339983
H	-0.55092089517932	5.80540034174104	6.74122754728057
C	0.77215394928217	7.43253216586878	7.24524337940254
H	1.39900814025243	7.29081515437310	6.36084257362380

H	0.05538223189412	8.22745132967329	7.01020842068559
C	1.73779215526963	4.58267078962825	6.80557104097772
H	2.24450349409411	5.40263630216500	6.29614908013072
H	1.02516828169076	4.11581347713306	6.11351910508723
H	2.48528472662033	3.84488547064990	7.10136532114351
C	1.01260063912957	8.73913956881725	9.34161260839387
H	-0.00027610477613	8.37508822962944	9.54344135459968
H	0.90262193212282	9.75144836840418	8.92979556700515
C	1.84784757682436	8.78441462947132	10.60154171688473
C	1.70403318507487	9.78214387545997	11.56073086004071
H	0.94148945970836	10.54715428612016	11.44209380288898
C	2.56682195945553	9.77420728176014	12.66041277738642
H	2.47042819244496	10.52836478018676	13.43789788800561
C	3.57678367205122	8.81365594534444	12.73131910843520
H	4.29262709492078	8.81494036190572	13.54858731399627
C	3.67632759592816	7.84003115657803	11.73136589989537
C	4.79425366707903	6.84516324263748	11.68686304654852
H	4.41442243456957	5.85506162513416	11.41876314335721
H	5.31301293217898	6.79172592578021	12.64615418215848
H	5.51879671958496	7.14904885305506	10.92027746489502
C	2.90711453502122	8.46458965558317	7.85129101059392
H	3.34999360383167	9.06755544989391	8.65103999581178
H	2.68090616433486	9.14051151785567	7.01916081030992
C	3.91594239418692	7.42860894308286	7.43971180993491
C	4.79174545210888	7.66920220797524	6.38523935498458
H	4.70168130526001	8.58666648464630	5.81048316559561
C	5.76672162875367	6.71554135071733	6.08723250567732
H	6.45853659557382	6.87141884749183	5.26296456112629
C	5.83279051932232	5.55888128728389	6.86043530770502
H	6.57713894116557	4.79349860045942	6.65851244119068
C	4.92379403373087	5.36326159042488	7.90682875594741
C	5.01110462478017	4.13634882446431	8.76036056031906
H	5.51829023783480	3.33065636089534	8.22446140368921
H	4.02166927301659	3.80237426566573	9.07783286288090
H	5.58910233067289	4.35801066318704	9.66686319200220
H	-0.44694833513394	6.70463448970327	10.62766796072433

[(susan$^{6\text{-Me}}$){Fe^{III}(μ-OH)(μ-1,2-OO)Fe^{III}}]$^{3+}$ (*μ-oxo-O3 protonated*):

Fe	1.42884544976954	3.81613093677939	12.01452810509171
Fe	2.22496815707718	6.17014263317884	9.57970858502284
O	0.33273876816983	5.19747902532292	11.48580684650713
O	0.65964959053528	6.22699070671852	10.65419793341724
O	2.71133180537206	4.64288624764175	10.66221472426652
N	0.77480929845829	2.26394514059247	10.43495247820323
N	2.90382515148941	2.26916496212742	12.40354920728831
N	2.75175009890888	4.68612518203721	13.56687436254255
N	0.28443731283736	2.68321203602966	13.40634507045634
N	1.02283907918153	5.07908619582676	8.02536643623165
N	1.61608092442705	7.86998957463173	8.39507515487892
N	2.77980617999636	7.95962567149357	10.73474289372921
N	3.96657999253961	6.36583917329374	8.21008469092168
C	1.10469766019173	2.75390169616009	9.06114076311377

H	0.95506935086646	1.92275221359787	8.35924203313963
H	2.16391200715048	3.01135044624042	9.04945044823292
C	1.58557418949548	1.04629105877196	10.73299552014711
H	1.63872500823760	0.39836800661001	9.84852024607178
H	1.06389873937472	0.48305049627794	11.51283842113721
C	2.98221566835494	1.40418427449205	11.19865935013434
H	3.55560908752338	0.49310692340374	11.41155400127817
H	3.52382790700405	1.95733874760134	10.42460860005151
C	-0.66085152966278	1.88207402567565	10.47784389323334
H	-0.89261081320561	1.44970414485001	11.45213430001772
H	-0.87618145161062	1.13657027628870	9.70100409288182
H	-1.29032305244168	2.75833047896490	10.31528820895028
C	4.19331632989341	2.91303400366947	12.72117703007499
H	4.66362731072682	3.24841185118213	11.78891274395407
H	4.89361495440743	2.20151078651468	13.17628912082678
C	3.96236204559022	4.08988489275807	13.63490354916557
C	4.97454592119466	4.55025366069561	14.47351291762453
H	5.92737502036240	4.02927619183325	14.50800109037423
C	4.73312449790855	5.68417683628356	15.24978035299686
H	5.50259514496554	6.07712714321354	15.90972627594000
C	3.48573025692465	6.30041388259876	15.16980746172063
H	3.25982303164676	7.18055645077508	15.76519314400132
C	2.49761301080041	5.77713630301676	14.32606583913017
C	1.13934488562912	6.40581729375327	14.25455914475652
H	0.91975204473521	6.76025697878003	13.24291568159548
H	1.07409424210793	7.24846012155298	14.94645372866748
H	0.37269382040583	5.67411767660795	14.53082639351612
C	2.43598844514411	1.52805379822853	13.60725421815861
H	2.91153492220322	1.98440128428190	14.48391977298059
H	2.77660601337454	0.48720369284174	13.56603435292899
C	0.95099984372457	1.58911737127585	13.83974526756749
C	0.32505567500371	0.57698067381852	14.56487489216567
H	0.89457567276528	-0.29464175875619	14.87526473560464
C	-1.02440107396928	0.71755436083739	14.88455427356878
H	-1.54218406491267	-0.05231966369009	15.45149632793508
C	-1.69826945154432	1.86409055383364	14.46520940379010
H	-2.74761820455809	2.01131252633044	14.70422365529713
C	-1.03190496681396	2.83590835329983	13.71209996243479
C	-1.76829106891383	4.04543725168306	13.21958157582551
H	-1.26087443754269	4.96808529220618	13.51230536521075
H	-2.78009293768434	4.05380385528544	13.63089293421628
H	-1.83721744821428	4.04799017082246	12.12683718433508
C	0.25376972752636	3.95965282270551	8.64666560714983
H	-0.51725925639242	3.64149609887245	7.93230556626192
H	-0.25383435598127	4.37994122590863	9.51583514427544
C	0.02963430054185	6.13125016422889	7.64840628860338
H	-0.62640672876657	6.27911472239215	8.51135184368036
H	-0.58399854525042	5.78455723289526	6.80614473638981
C	0.72434667506282	7.42843335145491	7.28449126182521
H	1.34751395362281	7.29922917705379	6.39556336264939
H	-0.01344544425590	8.20522130804726	7.05438864381829
C	1.74364599206657	4.59752612220648	6.82088388085046
H	2.25172338954903	5.42631377947788	6.32691019947507
H	1.03509633690710	4.14041498195224	6.11770223028136

H	2.49215531299707	3.85547895923865	7.10448909237663
C	0.94279198001093	8.76032517107323	9.37185798147629
H	-0.01827602588951	8.31082138341866	9.64365044212649
H	0.74576771385909	9.74551594671074	8.92960247888339
C	1.81928208354797	8.89600601429152	10.59649804546135
C	1.66796100022911	9.93012890301696	11.51689888728787
H	0.88732356230689	10.67342499517865	11.37893808800253
C	2.54377177529865	9.98406558131198	12.60448552327297
H	2.44456351533033	10.76949440576931	13.35003890389513
C	3.56726698733626	9.04016347647763	12.70601944772934
H	4.28962750692256	9.08321387652243	13.51678053405350
C	3.67422594202342	8.03205875504723	11.74125974238131
C	4.78251584947473	7.02349965521926	11.75672282474119
H	4.37775839168214	6.01799683863271	11.90477206655166
H	5.49271576876001	7.23518598284957	12.55860332271216
H	5.31595756176144	7.03754154904785	10.79945188273154
C	2.84224747957498	8.51046122869963	7.85992423358205
H	3.28057561692032	9.13701229874651	8.64310904709538
H	2.59303347732732	9.16496301719761	7.01761164726617
C	3.86808861888949	7.48673120665189	7.45538182872169
C	4.71744878404125	7.73875541007759	6.37890082253405
H	4.58734661383666	8.64504118846282	5.79404592648742
C	5.71791930493728	6.81634908508715	6.07644962726575
H	6.39229496007919	6.98265750103998	5.23999766109011
C	5.83444289273416	5.67620244968558	6.86949476727875
H	6.60374889975025	4.93458474471668	6.67301076034539
C	4.94581572156053	5.47042040486848	7.92966884043443
C	5.08702593103756	4.24123940018147	8.77987077451805
H	5.90720045051348	3.61813559420015	8.41802611796887
H	4.17742193080680	3.63032805814895	8.74440104557891
H	5.32309531889530	4.51617421475946	9.81680389310840
H	3.57355501341229	4.24133960632979	10.50061021446563

Supplementary References

1. Dammers, S. *et al.* A mixed-valence fluorido-bridged Fe^{II}-Fe^{III} complex. *Inorg. Chem.* **56,** 1779–1782 (2017).
2. Murata, Y. & Shine, H. J. Ion radicals. XVIII. Reactions of thianthrenium perchlorate and thianthrenium trichlorodiiodide. *J. Org. Chem.* **34,** 3368–3372 (1969).
3. Hausoul, P. J. C. *et al.* Facile access to key reactive intermediates in the Pd/PR3-catalyzed telomerization of 1,3-butadiene. *Angew. Chem. Int. Ed.* **49,** 7972–7975 (2010).
4. Crossland, P. M., Guo, Y. & Que, L. Spontaneous Formation of an Fe/Mn Diamond Core: Models for the Fe/Mn Sites in Class 1c Ribonucleotide Reductases. *Inorg. Chem.* **60,** 8710–8721 (2021).
5. Kütt, A. *et al.* A comprehensive self-consistent spectrophotometric acidity scale of neutral Brønsted acids in acetonitrile. *J. Org. Chem.* **71,** 2829–2838 (2006).
6. Eckert, F. *et al.* Prediction of acidity in acetonitrile solution with COSMO-RS. *J. Comput. Chem.* **30,** 799–810 (2009).
7. Kütt, A. *et al.* Equilibrium acidities of superacids. *J. Org. Chem.* **76,** 391–395 (2011).
8. Kütt, A. *et al.* Strengths of Acids in Acetonitrile. *Eur. J. Org. Chem.* **2021,** 1407–1419 (2021).
9. *SADABS* (Bruker AXS Inc., Madison, Wisconsin, USA, 2016).
10. Sheldrick, G. M. SADABS 2008. *SADABS,* 2008.
11. Sheldrick, G. M. SHELXT - integrated space-group and crystal-structure determination. *Acta Cryst. A* **71,** 3–8 (2015).
12. Sheldrick, G. M. Crystal structure refinement with SHELXL. *Acta Cryst. C* **71,** 3–8 (2015).
13. Dolomanov, O. V., Bourhis, L. J., Gildea, R. J., Howard, J. A. K. & Puschmann, H. OLEX2. A complete structure solution, refinement and analysis program. *J Appl Crystallogr* **42,** 339–341 (2009).
14. Neese, F. The ORCA program system. *WIREs Comput Mol Sci* **2,** 73–78 (2012).
15. Neese, F. Software update: the ORCA program system, version 4.0. *WIREs Comput Mol Sci* **8** (2018).

16. Neese, F., Wennmohs, F., Becker, U. & Riplinger, C. The ORCA quantum chemistry program package. *The Journal of Chemical Physics* **152,** 224108 (2020).

17. van Wüllen, C. On the use of common effective core potentials in density functional calculations. I. Test calculations on transition-metal carbonyls. *Int. J. Quantum Chem.* **58,** 147–152 (1996).

18. Weigend, F. Accurate Coulomb-fitting basis sets for H to Rn. *Phys Chem Chem Phys* **8,** 1057–1065 (2006).

19. Weigend, F. & Ahlrichs, R. Balanced basis sets of split valence, triple zeta valence and quadruple zeta valence quality for H to Rn: Design and assessment of accuracy. *Phys Chem Chem Phys* **7,** 3297–3305 (2005).

20. Pantazis, D. A., Chen, X.-Y., Landis, C. R. & Neese, F. All-Electron Scalar Relativistic Basis Sets for Third-Row Transition Metal Atoms. *J. Chem. Theory Comput.* **4,** 908–919 (2008).

21. Barone, V. & Cossi, M. Quantum Calculation of Molecular Energies and Energy Gradients in Solution by a Conductor Solvent Model. *J. Phys. Chem. A* **102,** 1995–2001 (1998).

22. Grimme, S., Ehrlich, S. & Goerigk, L. Effect of the damping function in dispersion corrected density functional theory. *J. Comput. Chem.* **32,** 1456–1465 (2011).

23. Grimme, S., Antony, J., Ehrlich, S. & Krieg, H. A consistent and accurate ab initio parametrization of density functional dispersion correction (DFT-D) for the 94 elements H-Pu. *The Journal of Chemical Physics* **132,** 154104 (2010).

24. Neese, F., Wennmohs, F. & Hansen, A. Efficient and accurate local approximations to coupled-electron pair approaches: An attempt to revive the pair natural orbital method. *The Journal of Chemical Physics* **130,** 114108 (2009).

25. Becke, A. D. Density-functional thermochemistry. III. The role of exact exchange. *J. Chem. Phys.* **98,** 5648 (1993).

26. Lee, Yang & Parr. Development of the Colle-Salvetti correlation-energy formula into a functional of the electron density. *Physical review. B, Condensed matter* **37,** 785–789 (1988).

27. Tao, J., Perdew, J. P., Staroverov, V. N. & Scuseria, G. E. Climbing the density functional ladder: nonempirical meta-generalized gradient approximation designed for molecules and solids. *Phys. Rev. Lett.* **91,** 146401 (2003).

28. Staroverov, V. N., Scuseria, G. E., Tao, J. & Perdew, J. P. Comparative assessment of a new nonempirical density functional: Molecules and hydrogen-bonded complexes. *The Journal of Chemical Physics* **119,** 12129–12137 (2003).
29. Neese, F. Prediction and interpretation of the 57Fe isomer shift in Mossbauer spectra by density functional theory. *Inorg. Chim. Acta* **337,** 181–192 (2002).
30. Römelt, M., Ye, S. & Neese, F. Calibration of modern density functional theory methods for the prediction of 57Fe Mössbauer isomer shifts: meta-GGA and double-hybrid functionals. *Inorg. Chem.* **48,** 784–785 (2009).
31. Sinnecker, S., Slep, L. D., Bill, E. & Neese, F. Performance of nonrelativistic and quasi-relativistic hybrid DFT for the prediction of electric and magnetic hyperfine parameters in 57Fe Mössbauer spectra. *Inorg. Chem.* **44,** 2245–2254 (2005).
32. Pápai, M. & Vankó, G. On Predicting Mössbauer Parameters of Iron-Containing Molecules with Density-Functional Theory. *J. Chem. Theory Comput.* **9,** 5004–5020 (2013).
33. Zimmermann, T. P. Synthese und Reaktivität biomimetischer, dinuklearer Eisenkomplexe. PhD thesis. Universität Bielefeld, 2019.
34. Zimmermann, T. P., Dammers, S., Stammler, A., Bögge, H. & Glaser, T. Reactivity Differences for the Oxidation of $Fe^{II}Fe^{II}$ to $Fe^{III}(\mu\text{-}O)Fe^{III}$ Complexes Caused by Pyridyl versus 6-Methyl-Pyridyl Ligands. *Eur. J. Inorg. Chem.* **48,** 5229–5237 (2018).
35. Bominaar, E. L. *et al.* Structural, Moessbauer, and EPR investigations on two oxidation states of a five-coordinate, high-spin synthetic heme. Quantitative interpretation of zero-field parameters and large quadrupole splitting. *Inorg. Chem.* **31,** 1845–1854 (1992).
36. Kostka, K. L. *et al.* High-valent transition metal chemistry. Moessbauer and EPR studies of high-spin (S = 2) iron(IV) and intermediate-spin (S = 3/2) iron(III) complexes with a macrocyclic tetraamido-N ligand. *J. Am. Chem. Soc.* **115,** 6746–6757 (1993).
37. Robin, M. B. & Day, P. Mixed-valence chemistry: a survey and classification. *Adv. Inorg. Chem. Radiochem.* **10,** 247–422 (1967).
38. Glaser, T. *et al.* Electronic structure of linear thiophenolate-bridged heterotrinuclear complexes $[LFeMFeL]^{n+}$ (M = Cr, Co, Fe; n = 1−3). Localized vs Delocalized Models. *J. Am. Chem. Soc.* **121,** 2193–2208 (1999).

39. Matthews, W. S. *et al.* Equilibrium acidities of carbon acids. VI. Establishment of an absolute scale of acidities in dimethyl sulfoxide solution. *J. Am. Chem. Soc.* **97,** 7006–7014 (1975).

40. Bordwell, F. G. Equilibrium acidities in dimethyl sulfoxide solution. *Acc. Chem. Res.* **21,** 456–463 (1988).

41. Kaljurand, I. *et al.* Extension of the self-consistent spectrophotometric basicity scale in acetonitrile to a full span of 28 pKa units: unification of different basicity scales. *J. Org. Chem.* **70,** 1019–1028 (2005).

42. Haav, K., Saame, J., Kütt, A. & Leito, I. Basicity of Phosphanes and Diphosphanes in Acetonitrile. *Eur. J. Org. Chem.* **2012,** 2167–2172 (2012).

43. Tshepelevitsh, S. *et al.* On the Basicity of Organic Bases in Different Media. *Eur. J. Org. Chem.* **2019,** 6735–6748 (2019).

44. Gerken, J. B., Pang, Y. Q., Lauber, M. B. & Stahl, S. S. Structural Effects on the pH-Dependent Redox Properties of Organic Nitroxyls: Pourbaix Diagrams for TEMPO, ABNO, and Three TEMPO Analogs. *J. Org. Chem.* **83,** 7323–7330 (2018).
